# Mechanically Robust Biodegradable Stents With Theragenerative Vascular Responses via Combined 3D Printing and Janus Nanoengineering

**DOI:** 10.1002/advs.202523965

**Published:** 2026-03-31

**Authors:** Jong Hwa Seo, Dong‐Sung Won, Hyun Lee, Seojoon Bang, Hyeong Seok Kang, Ju Yeong Gwon, Chan Ho Moon, Ji Won Kim, Yubeen Park, Minho Kang, Dong Yun Lee, Donghyun Lim, Kisuk Yang, Gi Doo Cha, Soo‐Hong Lee, Tae‐Sik Jang, Seokbeom Kim, Jun‐Kyu Park, Jung‐Hoon Park, Hyun‐Do Jung

**Affiliations:** ^1^ Division of Materials Science and Engineering Hanyang University Seoul Republic of Korea; ^2^ Department of Convergence Medicine Asan Medical Center University of Ulsan College of Medicine Seoul Republic of Korea; ^3^ Biomedical Engineering Research Center Asan Institute for Life Sciences Asan Medical Center Seoul Republic of Korea; ^4^ Research Institute of Intelligent Manufacturing & Materials Technology Korea Institute of Industrial Technology Incheon Republic of Korea; ^5^ Department of Bioengineering Hanyang University Seoul Republic of Korea; ^6^ Department of Biotechnology The Catholic University of Korea Bucheon Republic of Korea; ^7^ Department of Biomedical‐Chemical Engineering The Catholic University of Korea Bucheon Gyeonggi‐do Republic of Korea; ^8^ Division of Bioengineering College of Life Sciences and Bioengineering Incheon National University Incheon Republic of Korea; ^9^ Department of Systems Biotechnology Chung‐Ang University Anseong‐si Gyeonggi‐do Republic of Korea; ^10^ Department of Biomedical Engineering Dongguk University Seoul Republic of Korea; ^11^ School of Biomedical Convergence Engineering Pusan National University Yangsan Republic of Korea; ^12^ Plancklab Inc. Seoul Republic of Korea; ^13^ IMT Inc. Gwangju Republic of Korea

**Keywords:** 3D printing, biodegradable vascular stents, Janus nanoengineering, silica nanoparticles, tantalum ions

## Abstract

Peripheral artery disease (PAD), characterized by progressive occlusion of peripheral arteries, is a major global health concern associated with high risks of ischemic complications and limb dysfunction. Endovascular stenting remains a primary therapeutic approach; however, the development of biodegradable vascular stents that offer both sufficient mechanical resilience and antithrombotic, anti‐restenotic surfaces remains challenging, especially in highly deformable peripheral vessels. Herein, a 3D‐printed biodegradable drug‐eluting stent (DES) based on biofunctional silica–polycaprolactone nanocomposites and Janus surface nanoengineering is presented. Sol–gel‐derived silica incorporation and extrusion‐based 3D printing yield stents with tuned radial strength, elliptical struts that reduce flow disturbance, and enhanced support for endothelial regeneration. Janus nanoengineering is achieved through tantalum (Ta) plasma immersion ion implantation. The resultant nano‐Ta‐enriched luminal surface promotes human umbilical vein endothelial cell adhesion and proliferation. Meanwhile, the abluminal layer, comprising sirolimus/poly‐*L*‐lactic acid and nano‐Ta, suppresses vascular smooth muscle cell proliferation, reduces platelet thrombosis, and minimizes the initial burst release of therapeutic agents. Comprehensive in vitro hemocompatibility and cytocompatibility studies, combined with in vivo evaluation in a PAD model, demonstrate improved patency, reduced neointimal hyperplasia, and favorable tissue responses. This 3D‐printed, Janus‐engineered DES represents a promising theragenerative platform for vascular tissue engineering.

## Graphical abstract

This work introduces a theragenerative 3D‐printed biodegradable stent with Janus nanoarchitecture for spatially controlled vascular healing. A luminal tantalum ion–implanted surface accelerates endothelialization and hemocompatibility, while an abluminal sirolimus/poly‐L‐lactic acid–tantalum layer provides sustained drug release and suppresses smooth muscle hyperplasia. The platform restores luminal patency and reduces neointimal formation, offering a promising strategy for regenerative vascular intervention.

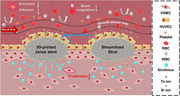

## 1 Introduction

Peripheral artery disease (PAD) is a progressive occlusive condition of the peripheral vasculature that significantly contributes to global morbidity and mortality, with its prevalence steadily rising worldwide. In 2021, an estimated 113 million adults globally were reported to have PAD, with this number projected to exceed 363 million by 2050 [1, 2]. PAD is primarily caused by atherosclerotic narrowing of the lower extremity arteries arising from a complex combination of risk factors, including hypertension, diabetes, dyslipidemia, smoking, and chronic endothelial dysfunction. However, its underlying pathogenesis remains multifactorial and not yet fully defined, often resulting in impaired perfusion, intermittent claudication, pain at rest, and critical limb ischemia that can lead to irreversible tissue loss [3, 4, 5]. As atherosclerotic plaque accumulates, peripheral arteries gradually harden and narrow, often necessitating revascularization to restore blood flow. Consequently, pharmacological treatment, such as antiplatelet agents or vasodilators, is essential. However, these treatments are generally effective only in the early stages of disease, particularly to alleviate claudication. If pharmacological treatment is insufficient or ischemic symptoms progress, endovascular interventions, including balloon angioplasty and stent implantation, may be required to mechanically reopen the stenotic vessel [6, 7]. These procedures involve advancing an expandable balloon catheter into the occluded region to compress plaque against the arterial wall, followed by deployment of a self‐ or balloon‐expandable stent to maintain vessel patency after balloon removal [3]. While this approach supports vascular remodeling and has become a key strategy in modern PAD management, the complex mechanical environment of peripheral arteries—characterized by frequent bending, torsion, stretching, and external compression—poses significant challenges to stent durability and long‐term patency [8]. Accordingly, interest in biodegradable vascular stents (BVSs) continues to grow, particularly in below‐the‐knee lesions where ease of procedure and device delivery are crucial [9, 10]. Recently, Abbott's Esprit BTK absorbable scaffold system demonstrated promising therapeutic outcomes specifically for PAD, demonstrating the expanding relevance and transformative potential of the BVS platform in treating PAD [11].

Currently, drug‐eluting stents (DESs) are widely used as an alternative clinical treatment for PAD [12, 13, 14]. Generally, stents coated with anti‐proliferative or immunomodulatory drugs provide localized therapy at the vascular injury site by limiting VSMC hyperplasia and reducing neointimal formation. Notably, the incidence of in‐stent restenosis (ISR) has been substantially reduced through DESs incorporating widely employed therapeutics, including paclitaxel and sirolimus [15, 16, 17, 18]. Nonetheless, metal‐based DESs are non‐degradable and can remain in the blood vessel semi‐permanently, potentially causing complications such as chronic vasomotion irritation, inflammatory responses, or long‐term endothelial dysfunction [19, 20]. Moreover, metal ions released from metallic stents into the bloodstream can trigger allergic reactions and hemotoxic effects, such as thrombus formation and inflammatory responses. In addition, corroded BMSs or DESs during long‐term implantation may accelerate atherosclerosis [21, 22]. Consequently, biodegradable polymers offer a promising alternative as stent materials. These polymers provide mechanical support to the vessel wall during the healing process (approximately one year) and are eventually fully absorbed, eliminating the need for surgical removal once healing is complete [23].

A range of biodegradable polymers approved by the US Food and Drug Administration (FDA), including poly(lactic‐*co‐*glycolic acid), poly‐*L*‐lactic acid (PLLA), and poly‐ε‐caprolactone (PCL), has long served as structural matrices or coating layers in polymeric BVSs and DESs, owing to their favorable biodegradation profiles, chemical safety, and excellent biocompatibility with multiple therapeutic agents [24, 25, 26, 27]. However, these synthetic biodegradable polymers exhibit relatively poor hemocompatibility, which can lead to delayed re‐endothelialization and an increased risk of long‐term thrombosis [28]. Moreover, the commercially available PLLA‐based BVS Absorb, fabricated by Abbott, exhibited poor anticoagulant activity, high rates of late‐stage thrombosis, and inadequate radial support to the vessel wall and was subsequently withdrawn from the market [29]. This clinical failure has been attributed to a combination of insufficient surface modification and suboptimal stent design. Although PLLA provided the stent with the required biodegradability, the pristine PLLA surface lacks intrinsic biological properties, including favorable vascular tissue responses.

In our previous studies, surface nanoengineering via Ta ion embedding or implantation—achieved through target‐ion induced plasma sputtering or sputtering‐based plasma immersion ion implantation (S‐PIII), respectively—markedly enhanced cellular responses both in vitro and in vivo [30, 31, 32, 33, 34, 35]. These findings highlight its promising utility in biomedical fields and vascular interface engineering. In particular, S‐PIII has emerged as an efficient and versatile approach for polymer surface functionalization through high‐flux implantation of metallic (e.g., Ta) ions. In this process, ions accelerated within a dense plasma environment are homogeneously implanted into the topmost polymer surface, enabling precise control of its interfacial properties [35]. The S‐PIII‐modified layers exhibit superior interfacial adhesion, favorable surface wettability, and high biocompatibility. Moreover, the implanted layer acts as a structural barrier, effectively mitigating the early burst release of the loaded drug. In terms of stent design, biodegradable polymeric stents require thicker struts than non‐degradable BMSs to maintain sufficient mechanical strength. However, such struts can increase thrombogenicity by activating platelets and reducing anticoagulant factors due to greater local blood flow disturbances within the vessel lumen [36, 37]. Moreover, conventional stent manufacturing methods, such as braiding and laser cutting, have inherent limitations. Braiding can reduce mechanical performance through simple design, resulting in poor radial strength, while laser cutting can compromise fatigue resistance due to thermal damage [38, 39, 40]. The size range of stents fabricated by these techniques is also limited, which makes patient‐specific customization difficult. Given the variability in peripheral artery pathologies, including lesion types, patient age, gender, and physiological condition, there is a strong need for stent materials that can be tailored to more effectively treat PAD.

The reversible melting and cooling behavior of biodegradable thermoplastic polymers allows them to be processed through material extrusion‐based 3D printing. This approach enables patient‐specific designs for biomedical applications, such as wound dressing, orthopedic devices, drug delivery systems, and vascular implants [41, 42]. Additionally, compared to conventional stents with laser‐cut rectangular struts, 3D‐printed stents with circular struts have a smaller cross‐section and reduced flow disturbances, thereby providing a more favorable environment for normal endothelial function [37]. Therefore, in recent years, various stents fabricated using extrusion‐type 3D printing systems equipped with coordinated extruder–mandrel assemblies operating along the *x‐Ɵ‐z* axis have been investigated to accommodate patient‐specific vascular lesions and their dimensions [43, 44, 45]. In particular, combining 3D printing with magnetic resonance angiography systems could enable the fabrication of complex, patient‐specific peripheral stents. However, beyond patient customization, optimizing the mechanical and functional performances of 3D‐printed polymer‐based stents, whether currently in use or under investigation, remains challenging.

The development of polymer‐based functional nanocomposites combines the intrinsic characteristics of inorganic nanoparticles (NPs) with the flexibility of polymer matrices, achieving synergistic performance unattainable by the polymer alone [46]. A broad spectrum of inorganic NPs has been utilized to facilitate diverse polymer functionalization systems for reinforcement, in which the NPs are uniformly dispersed and incorporated via physicochemical interactions within the polymeric framework [47, 48, 49, 50]. Recently, silica (silicon oxide, SiO_2_) NPs, recognized as typical bioactive nanotherapeutics based on metal oxides, have demonstrated capabilities in drug loading, promoting osteogenesis, ensuring biocompatibility, and enhancing angiogenesis [51, 52, 53, 54]. In vascular tissue applications, silica NPs have garnered significant attention. The silicon ions released from functionalized polymers positively influence endothelial cells during the initial adhesion, migration, and proliferation stages of PAD healing. These effects are mediated through the activation of hypoxia‐inducible factor 1*α* and the vascular endothelial growth factor signaling pathway [46, 55, 56, 57]. Furthermore, the incorporation of silica into polymer matrices, including PCL, PLLA, and polyurethane, provides effective mechanical reinforcement, leading to improved structural integrity and mechanical properties suitable for clinical deployment [58, 59].

**FIGURE 1:**
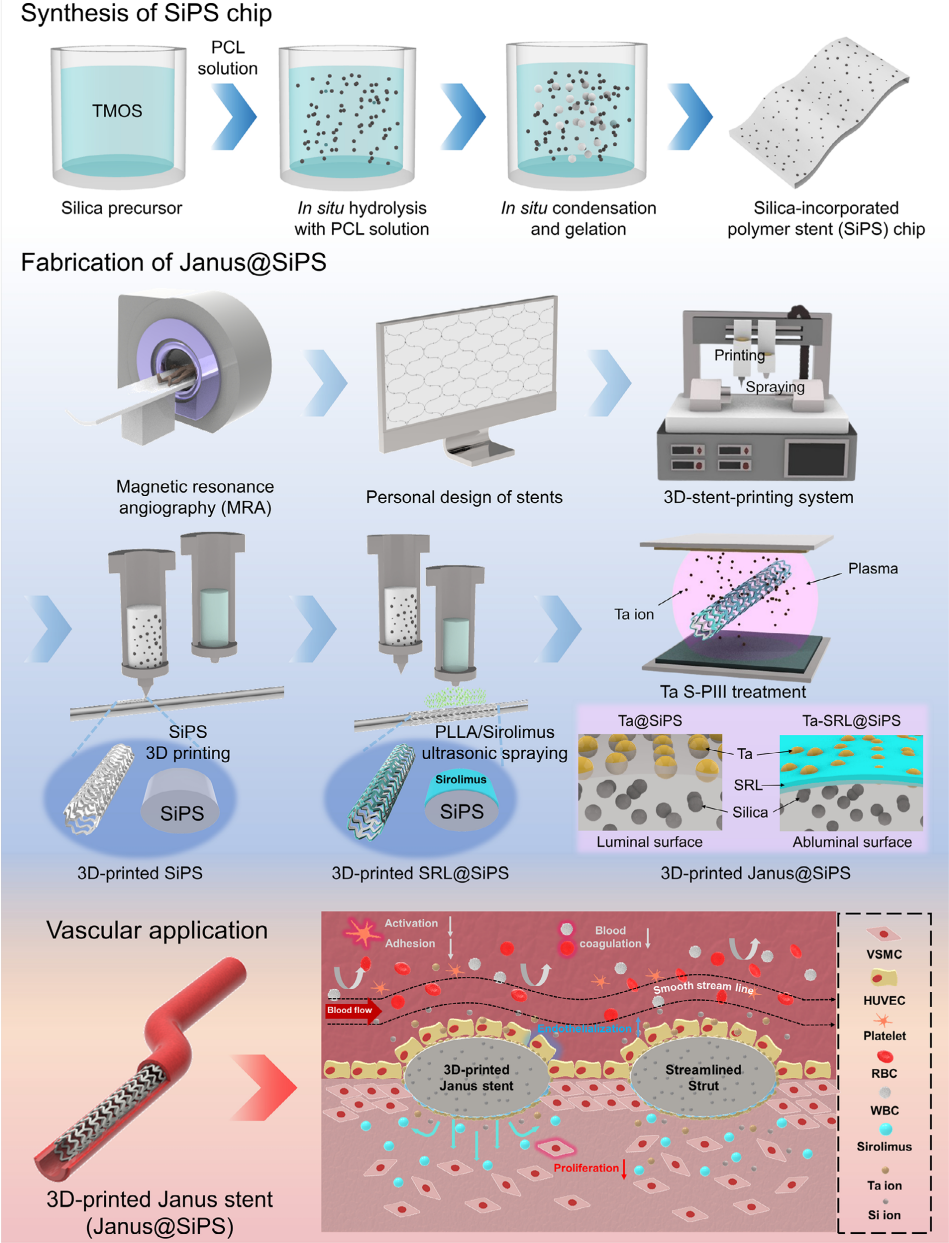
Schematic illustration of the fabrication and vascular application of 3D‐printed Janus‐nanoengineered biodegradable SiPSs. Schematic overview showing the synthesis of silica‐incorporated polymer stent (SiPS) chips for 3D printing, the fabrication of patient‐specific biodegradable stents via combined 3D printing and Janus nanoengineering, and the resulting favorable vascular responses. The streamlined, elliptical stent struts minimize flow disturbance and support endothelial function, while the Janus‐nanoengineered surfaces (luminal Ta ion‐implanted layer and abluminal sirolimus/PLLA‐Ta composite) provide dual theragenerative function. This design offers great potential for PAD treatment by controlling drug release, reducing platelet activation, and inhibiting VSMC proliferation. (SRL@SiPS: sirolimus‐coated SiPS, Janus@SiPS: Janus‐nanoengineered SiPS).

Motivated by these previous results, we developed a 3D‐printed silica‐incorporated polymer stent (SiPS) with Ta ion implantation, creating a promising theragenerative platform for vascular tissue engineering in PAD. This 3D‐printed biodegradable DES platform is based on a bioactive silica NP–polymer nanocomposite and Janus nanoengineering incorporating sirolimus and Ta ions via S‐PIII treatment. By integrating the synergistic strengths of inorganic NPs and polymer‐based organic biomaterials, 3D‐printed SiPSs are designed to tune the mechanical performance and enhance human umbilical vein endothelial cell (HUVEC) regeneration. Furthermore, the Janus‐nanoengineered surface layer of a SiPS can synergistically enhance vascular cellular responses. This is achieved through a luminal surface embedded solely with Ta ions, while the abluminal surface, coated with sirolimus/PLLA and implanted with Ta ions, facilitates controlled drug release (Figure 1). This configuration effectively inhibits VSMC proliferation and platelet activation, offering significant potential for application across diverse PAD therapies. The intrinsic physicochemical properties and resultant biological responses of the 3D‐printed biodegradable DES—encompassing in vitro biocompatibility and in vivo therapeutic efficacy for PAD—were systematically examined and critically interpreted.

## 2 Results and Discussion

### 2.1 Fabrication of 3D‐Printed SiPSs

Peripheral vascular disorders are typically associated with impaired vascular function, resulting in reduced delivery of nutrients and oxygen to tissues due to partial narrowing or complete occlusion of the arterial lumen [60]. In response, revascularization by expanding and supporting the stenotic or occluded blood vessels with BVSs has been used as an effective therapeutic approach for saving patients. Although several natural and synthetic polymers have been adopted for biodegradable vascular applications such as stents and grafts, achieving reliable clinical outcomes with structural integrity remains challenging. PCL, a representative candidate material for BVSs, provides temporary structural support during vascular regeneration and exhibits favorable biocompatibility and biodegradability without residual by‐products. To address its inherent hydrophobicity and relatively weak mechanical strength, various hybrid systems combining PCL with organic and inorganic biomaterials have been developed. For long‐term patency, the strategy focuses on promoting rapid endothelialization. A functional endothelium not only inhibits neointimal hyperplasia by releasing growth‐regulatory molecules and cytokines but also prevents platelet adhesion and activation by releasing anticoagulant factors [31]. Anti‐thrombogenic drugs or organic biomolecules, such as heparin, gelatin, and serum albumin, have been commonly applied to the luminal surface to promote the formation of a confluent endothelial layer [33]. Another approach to promote rapid endothelialization is the incorporation of metal oxide‐based functionalities, which can affect vascular responses [24, 34]. This strategy serves a dual purpose, facilitating drug delivery while simultaneously strengthening the physical and biological properties of the stent. Consequently, it has proven highly effective for fabricating and coating stents that promote rapid endothelial formation, thereby supporting durable functional performance over extended implantation periods. In our earlier study, sol–gel‐derived silica displayed superior wettability and bioactivity, while allowing facile incorporation into polymeric matrices without inducing segregation or phase separation. Consequently, a homogeneous nanostructured fiber comprising a PCL polymer and silica NPs or a coating layer was achieved, which exhibited improved mechanical strength, flexibility, water affinity, and cell compatibility. Building on these findings, we developed an innovative 3D‐printed BVS composed of a multifunctional silica‐incorporated polymer. The silica within the polymer matrix is anticipated to provide mechanical stability and modulate the degradation rate, while surface silica acts as nucleation points for rapid endothelialization.

**FIGURE 2:**
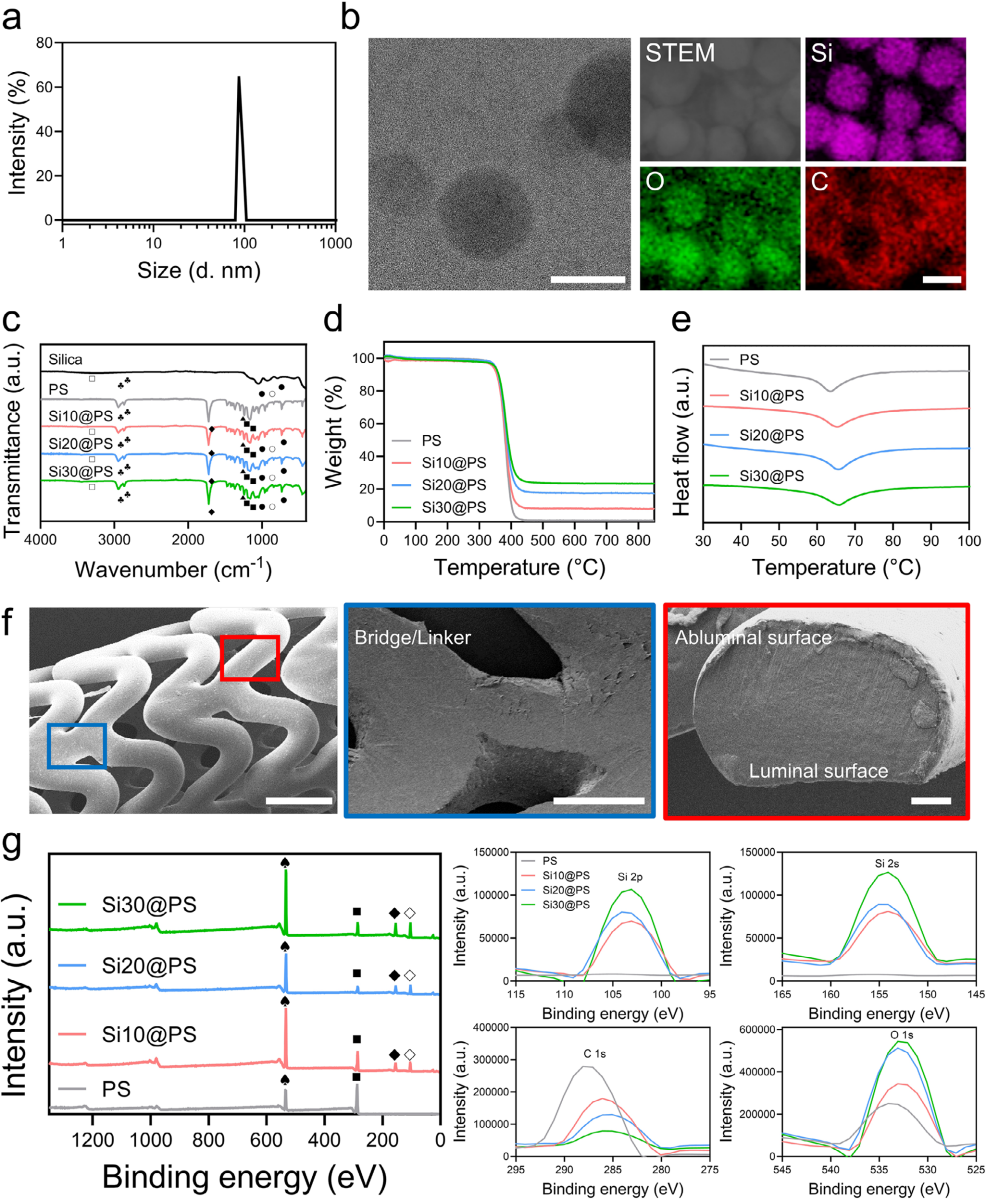
Fabrication and characterization of 3D‐printed SiPSs with various silica concentrations. (a) DLS analysis showing the size and distribution of sol–gel‐derived silica NPs within the polymer matrix (*n* = 3). (b) TEM, STEM, and elemental mapping observations of the sol–gel‐derived silica NPs in the polymer matrix (scale bar: 100 nm). (c) FT‐IR spectra of sol–gel‐derived silica, PS, and SiPS chips with varying silica contents (●: Si─O─Si stretching,°: Si─OH stretching, □: O─H stretching, ■: C─O─C stretching, ▲: C─C and C─O stretching, ♦: C─O stretching, ♣: CH_2_ stretching) (*n* = 3). (d) TGA and (e) DSC curves of SiPS chips containing various silica contents. (f) Representative FE‐SEM images showing the overall stent surface (scale bar: 1 mm), the bridge/linker surface is highlighted with a blue box (scale bar: 300 µm), while the cross‐sectional strut of the stent is highlighted with a red box (scale bar: 50 µm). (g) Representative high‐resolution XPS spectra of SiPS with varying silica contents (♠: O 1s, ■: C 1s, ◆: Si 2s, ◇: Si 2p).

To verify the successful incorporation and dispersion of silica NPs within the PCL matrix, their size and distribution were analyzed using dynamic light scattering (DLS). As shown in Figure 2a, the silica in the PCL solution exhibited a hydrodynamic size distribution, with a peak observed in the range of 80–100 nm. This result is consistent with transmission electron microscopy (TEM) observations, which revealed spherical NPs in the range of 80–110 nm distributed within the polymer matrix. Scanning transmission electron microscopy (STEM) images and elemental mapping using energy dispersive X‐ray spectroscopy (EDS) confirmed that the spherical NPs were composed of Si and O, which are characteristic of silica NPs. In addition to these signals, weak C and O signals from the polymer backbone were detected and uniformly distributed throughout the SiPS matrix. These results confirm that silica NPs synthesized via the sol–gel process were uniformly dispersed within the polymer matrix (Figure 2b).

To further elucidate the chemical interactions between silica and the polymer matrix, Fourier transform infrared (FT‐IR) spectroscopy was conducted to investigate the functional bonding characteristics of the SiPS chips. As shown in Figure 2c, FT‐IR spectra of the sol–gel‐derived silica displayed characteristic features of a silicate network. These included a Si─O─Si asymmetric stretching vibration at 1064 cm^−1^, a Si─OH asymmetric bending and stretching vibration at 943 cm^−1^, a Si─O─Si symmetric stretching vibration at 833 cm^−1^, and an O─H stretching vibration at 3309 cm^−1^ [61, 62]. For the polymer stent (PS) chips, distinct absorption bands were observed, including C─O─C stretching at 1167 cm^−1^, asymmetric C─O─C stretching at 1240 cm^−1^, C─C and C─O stretching at 1296 cm^−1^, asymmetric C═O stretching at 1723 cm^−1^, and asymmetric and symmetric CH_2_ stretching at 2928 and 2856 cm^−1^, respectively [58]. In the SiPS chips, additional peaks corresponding to the combined silica–polymer matrix were detected, confirming the successful incorporation of sol–gel‐derived silica into the polymer matrix. However, comparison of the FT‐IR spectra of silica NPs and SiPS chips revealed a shift in the hydroxyl (O─H) stretching band from 3309 to 3294 cm^−1^. Furthermore, the carbonyl (C═O) stretching peak of the SiPS chip shifted slightly—from 1722 to 1720 cm^−1^—compared with that of the bare PS chip. These shifts suggest intermolecular hydrogen‐bond formation between the silica hydroxyl and polymer carboxyl groups (Figure S1) [63]. Previous research on polymer‐based hybrid materials and nanocomposites incorporating silica NPs has reported a similar phenomenon [64, 65, 66]. Owens et al. categorized inorganic–organic composite materials into two types, Class I and Class II, according to the nature of the chemical interactions between their inorganic and organic components. In these composites, hydrogen bonds are formed between the amide, carbonyl, and carboxyl groups of the functionalized polymer and the hydroxyl groups of metal oxides synthesized via sol–gel processing. These interactions influence the mechanical properties of the resultant materials. FT‐IR results indicate that the synthesized SiPS chips exhibited similar hydrogen‐bonding interactions between silica and the polymer, thereby improving both the mechanical and physicochemical properties of the material.

The thermal stability and degradation behavior of SiPS chips containing different silica concentrations were analyzed using thermogravimetric analysis (TGA) and differential scanning calorimetry (DSC). TGA analysis revealed that all SiPS samples exhibited a single‐step degradation process. However, the bare PS chip exhibited no appreciable weight loss up to its decomposition temperature (∼350°C), whereas silica‐containing polymers displayed a slight weight loss occurring prior to polymer degradation [41, 63, 67]. This tendency became more pronounced at higher silica loadings, as condensation of surface silanol groups promoted moisture loss during thermal treatment [68]. Notably, the decomposition temperature of the SiPS chips increased with increasing sol–gel‐derived silica content, indicating that these chips exhibited greater thermal stability compared with the bare PS chip (Figure S2). This physicochemical reinforcement of the polymer can influence the thermal behavior, particularly the degradation temperature. The thermal stability of SiPS chips was enhanced with increasing silica loadings. Homogeneous SiPS chips, composed of silica and PCL linked via hydrogen bonding, were obtained using sol–gel‐based synthesis of organic–inorganic biomaterials. The improved thermal stability broadens the viable processing window, offering more reliable conditions for extrusion and 3D printing in commercial manufacturing. Moreover, thermal decomposition occurred below 500°C, and the residual mass measured at 600°C increased from 0.7 wt.% in the bare PS chip to 23.3 wt.% in the Si30@PS chip, which is attributed to the thermally stable inorganic silica remaining after polymer decomposition. The increased residual mass fraction qualitatively reflects the higher silica loading introduced into the polymer matrix and is consistent with the intended composition ratio (Figure 2d). DSC analysis revealed a slight increase in the melting temperature (*T*_m_) from 63.4°C for the bare PS chip to 65.6°C for Si30@PS, corresponding to a rise of 2.2°C. This moderate elevation in *T*_m_ can be attributed to subtle changes in the crystallization kinetics of the SiPS chips [69]. Generally, the incorporation of reinforcing components into a polymer induces heterogeneous crystallization with partially incomplete crystals, which can increase the melting temperature [70]. Thus, we assumed that the silica–polymer interfacial interactions (i.e., hydrogen bonding between the two phases) partially disrupted crystallinity, which in turn led to an increase in the *T*_m_ of the SiPS chips (Figure 2e).

Based on the synthesized SiPS chips, BVSs were fabricated using an additive 3D printing process. Bare PS and SiPS containing 10–30 wt.% silica were thermally processed and subsequently extruded through a stent 3D printing system under optimized printing parameters (Table S1). During extrusion, the SiPS was deposited from the nozzle onto a rotatable stainless‐steel mandrel. Using this mandrel, stent struts could be constructed following diverse 3D deposition path designs. In this study, an elliptical structural geometry was selected for the stent scaffold. The manufactured SiPS retained a stable tubular configuration, and the printing process accommodated a wide range of diameters, lengths, and geometrical designs (Figure S3).

The surface features of a stent are important because its 3D structure directly affects both expansion capability and the adhesion and proliferation of tissue cells during the repair process. In particular, the microstructure of the stent struts, such as surface roughness, is an important factor since it critically affects thrombus formation and platelet adhesion. The microstructures of the 3D‐printed SiPS samples were studied using field emission scanning electron microscopy (FE‐SEM). As shown in Figure 2f, the 3D‐printed stent presented a typical architecture with uniformly organized and interconnected bridges, consistent with the CAD design. At higher magnification, the 3D‐printed SiPS revealed a dense and smooth microstructure, intentionally designed to prevent platelet adhesion and thrombosis formation [71, 72]. The struts of the 3D‐printed stent presented an elliptical cross‐sectional shape with respective long and short axis diameters of approximately 280 and 200 µm. Alongside strut thickness, strut shape is a key factor influencing vascular responses, such as inflammation and neointimal hyperplasia (NIH). Jimenez et al. conducted blood flow dynamics studies with rectangular and circular strut stents. They reported that streamlined struts, such as circular and elliptical shapes, minimize or eliminate recirculation zones, which could inhibit endothelial regrowth [37]. Struts produced via fused filament fabrication and 3D printing tend to exhibit an elliptical rather than circular shape, thereby increasing the contact area and adhesion strength between successive layers [41]. Therefore, based on this well‐known strategy, we employed 3D‐printed stents with streamlined elliptical struts that could provide a more favorable environment for normal endothelial function by reducing blood flow disturbances (Figure 1). Quantitative EDS analysis confirmed the successful incorporation of silica within the PCL matrix. For Si30@PS, the silica content was quantified at 11.1 at%, indicating a homogeneous distribution throughout the cross‐section of the 3D‐printed stent strut. These findings indicate that sol–gel‐derived SiPS enabled well‐dispersed silica NPs within the PCL matrix (Figure S4). X‐ray photoelectron spectroscopy (XPS) was conducted to further examine the surface elemental characteristics resulting from silica incorporation. Consistent with the quantitative EDS results, the intensities of the Si 2p (103 eV), Si 2s (153 eV), and O 1s (532 eV) peaks, originating from the silica component, increased with increasing silica content. On the other hand, as the surface concentration of silica NPs increased, the intensity of the polymer‐originated C 1s peak decreased and shifted from 288 to 285 eV, indicating that silica was uniformly and precisely distributed within the PCL matrix (Figure 2g) [73, 74].

### 2.2 Characterization of 3D‐Printed Janus‐Nanoengineered SiPSs

In our previous study analyzing the physical properties of SiPS as a function of silica concentration, the 3D‐printed SiPSs with higher silica contents exhibited markedly improved mechanical strength. However, the reduction in polymer ductility outweighed the reinforcing effect, even in the absence of aggregated silica NPs. This could lead to potential stent failure during crimping and expansion. Nevertheless, despite this limitation, the material still exhibited greater deformability than commercial PLLA, the polymer most commonly used for BVS fabrication. Therefore, Si20@PS was selected as the fundamental material for the 3D‐printed stents, and subsequent Janus processing and characterization were carried out [63]. In this work, Ta ion implantation was applied to the surface of the 3D‐printed SiPS to fabricate a Janus surface, with each opposing side exhibiting distinct functional properties. The luminal SiPS surface, embedded with nano‐Ta ions, synergistically promoted responses of HUVECs. In contrast, the abluminal surface, coated with a sirolimus layer also embedded with Ta ions, inhibited VSMC proliferation and platelet activation, without an initial burst release of the drug. Previous studies have shown that Ta has primarily been used in cardiovascular applications, including vascular stents, owing to its excellent blood compatibility, inherent radiopacity, and excellent corrosion resistance [47]. Despite these favorable biological properties, the utilization of biometals as coatings on polymer surfaces remains limited because of structural and mechanical mismatches between the metal coatings and polymer substrates. Insufficient interfacial strength of the coating layers can lead to the formation of fragments and particles, which may disrupt cellular functions such as cytokine release, increase macrophage apoptosis and cytotoxicity, and ultimately contribute to stent failure [48]. Our unique S‐PIII process provides a robust and versatile approach for polymer surface modification. In this technique, high‐energy ions are physically implanted onto the polymer surface, achieving good dispersion within a controlled depth without forming an additional layer on the external substrate surface. This overcomes the limitations of conventional coatings, such as cracking and/or detachment. By applying a negative bias of approximately 2000 V to the substrate holder, high‐energy Ta ions (∼10 keV) were sufficiently accelerated to implant into the shallow surface of the SiPS (luminal side) and sirolimus‐containing PLLA layer (abluminal side). This process resulted in the formation of an implanted Ta layer within both surfaces.

**FIGURE 3:**
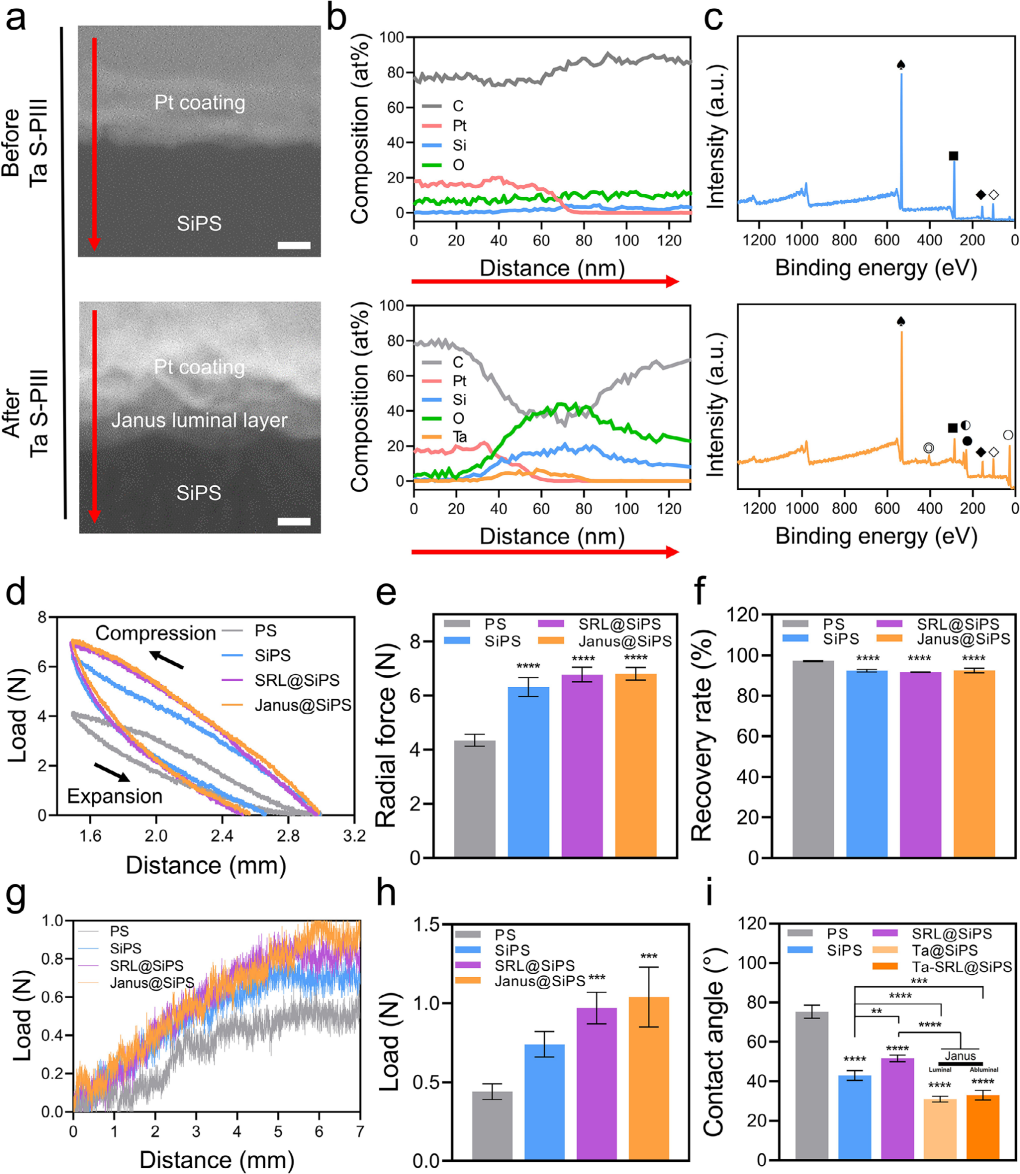
Physicochemical characterization of 3D‐printed Janus‐nanoengineered SiPSs. (a) FIB‐STEM cross‐sectional images (scale bar: 20 µm) and (b) EDS elemental spectra of C, O, Si, and Ta in 3D‐printed SiPSs, both untreated and after Ta S‐PIII treatment. (c) XPS profiles before and after Ta S‐PIII treatment (♠: O 1s, ◎: Ta 4p, ■: C 1s, ◐: Ta 4d_3/2_, ●: Ta 4d_5/2_, ◆: Si 2s, ◇: Si 2p,°: Ta 4f). Radial compression test results showing (d) load–distance curves, (e) radial force (*n* = 3), and (f) recovery rate of PS, SiPS, SRL@SiPS, and Janus@SiPS (*n* = 3). (g) Bending test results and (h) corresponding flexibility load values (*n* = 3). (i) Water contact angles of different surfaces of 3D‐printed stents (*n* = 3). Data are shown as mean ± standard deviation (SD). Normality was tested using the Shapiro–Wilk method, and one‐way ANOVA followed by Tukey's HSD post hoc analysis was applied, with significance at *p < 0.05, **p < 0.01, ***p < 0.005, and ****p < 0.001.

To evaluate the Ta‐applied layer in both its untreated state and after Ta S‐PIII treatment, the cross‐sectional morphology of the 3D‐printed stents was examined using focused ion beam‐transmission electron microscopy (FIB‐TEM). In addition, EDS and XPS were performed to provide a more detailed characterization of the Ta‐embedded surface layer, particularly its thickness and elemental composition after S‐PIII treatment. FIB‐TEM imaging and EDS line profile analysis revealed two clearly separated domains including an unmodified SiPS matrix and a platinum layer. Following Ta S‐PIII treatment, a brighter contrast layer of approximately 20 nm was observed at the outermost surface of the SiPS, indicating that Ta ions had penetrated to this depth without forming a discrete interface between the implanted region and the underlying substrate, as evidenced by line analysis (Figure 3a). The line‐scan profile exhibited a parabolic trend, with the Ta concentration peaking at approximately 7 at% near the surface and gradually decreasing with increasing depth (Figure 3b; Figure S5). The surface chemical composition of the 3D‐printed SiPSs, in both the untreated state and after Ta S‐PIII treatment, was analyzed using XPS. As shown in Figure 3c, all samples consistently displayed distinct C 1s (285 eV) and O 1s (532 eV) signals, corresponding to C─ C, O═C─O, Si─OH, and Si─O─Si functionalities, irrespective of Ta S‐PIII treatment [75, 76, 77]. In addition, all samples displayed characteristic peaks at 153 and 103 eV, corresponding to the Si 2s and Si 2p states derived from the silica phase within the SiPS. Notably, the Si 2p signal could be further deconvoluted into contributions from Si─O (102.4 eV) and Si─OH (103.2 eV) species. These spectral features were identical to those previously observed in Figure 2g, where different silica concentrations were compared [76]. In contrast, four new peaks corresponding to Ta appeared in the XPS profile of the SiPS surface following Ta S‐PIII treatment: 404 eV (Ta 4p), 242 eV (Ta 4d_3/2_), 230 eV (Ta 4d_5/2_), and ∼27 eV (Ta 4f). The high‐resolution spectrum of the Ta 4f region revealed peaks at 26 eV (Ta 4f_7/2_) and 28 eV (4f_5/2_), along with very‐low‐intensity peaks of Ta^0^ at 21.5 and 23.3 eV. These signals correspond to the typical chemical state of Ta in Ta_2_O_5_ and are in good agreement with previously reported values (Figure S6a). Additionally, the O 1s spectrum of the SiPS surface after Ta S‐PIII treatment was broader than that of the untreated SiPS surface. This was attributed to the formation of Ta─O bonds in silica or Ta_2_O_5_ along with hydroxyl groups (Ta─OH) originating from the outer surface or from silanol groups in the SiPS silica phase (Figure S6b) [77, 78, 79, 80].

Subsequently, an ultrathin spray coating using a PLLA solution containing sirolimus was selectively applied only to the abluminal surface of the stent, followed by the implantation of Ta ions throughout the stent using the Ta S‐PIII process, resulting in a Janus‐structured stent. This asymmetric surface design is a crucial strategy for enabling selective drug action while simultaneously maintaining the mechanical and surface benefits of Ta ion implantation [30, 63, 81]. Specifically, to ensure the local therapeutic effect of sirolimus, it is essential to clearly demonstrate that the drug exists only on the abluminal surface. Therefore, SEM‐EDX analysis was performed to identify the compositional differences between the abluminal and luminal surfaces of the Janus@SiPS. As shown in Figure S7a,b, the abluminal and luminal surfaces of the Janus@SiPS were simultaneously observed using low‐magnification SEM images. Subsequently, the areas marked by the red, blue, and purple boxes were gradually magnified. Finally, EDX analysis was performed on the purple box, corresponding to the highest magnification, to precisely compare the compositions of each surface. EDX mapping of the abluminal surface revealed that organic components, C and O, were clearly detected throughout the surface, consistent with the basic composition of SiPS. Furthermore, Si derived from the silica phase and Ta implanted through the Ta S‐PIII process were observed. This trend was confirmed in the EDX spectrum, where C and O peaks were clearly visible, along with elemental peaks for Si and Ta. The luminal surface also exhibited a similar qualitative trend, with C and O being the primary constituents, followed by Si and Ta. However, quantitative element analysis (wt.%) revealed significant differences between the two surfaces. The abluminal surface measured C and O contents of 24.4 ± 2.2 and 23.9 ± 1.8 wt.%, respectively, indicating a relatively high proportion of organic components compared to the luminal surface. On the other hand, Si and Ta contents were 17 ± 1.4 and 34.7 ± 5 wt.%, respectively. Conversely, on the luminal surface, C and O were measured at 18.7 ± 1.8 and 20.3 ± 1.5 wt.%, respectively. Si remained at a similar level at 17.8 ± 1.3 wt.%, while Ta showed a higher content at 43.2 ± 6.5 wt.%. This can be interpreted as a result of the relatively large contribution of Ta on a wt.% basis due to its high atomic weight (Figures S8 and S9). These quantitative compositional differences demonstrate that the Janus@SiPS stent has a clearly distinct surface composition between the abluminal and luminal surfaces. In particular, the relatively high proportion of organic components observed on the abluminal surface indirectly supports the selective formation of the PLLA coating, including sirolimus, on the abluminal surface, given that both PLLA and sirolimus are organic‐based materials. Therefore, these SEM–EDX analysis results effectively verify the successful fabrication of the Janus‐structured stent and the spatially selective distribution of the drug [17, 82, 83, 84].

### 2.3 Mechanical Evaluation of 3D‐Printed Janus‐Nanoengineered SiPSs

Although our previous study demonstrated that Si20@PS exhibits favorable biodegradability and biofunctionality, adequate mechanical performance is equally critical to ensure reliable expansion and structural stability within the vascular environment [63]. Therefore, the mechanical influence of Janus surface modification was investigated by evaluating the radial force and bending behavior of the 3D‐printed SiPSs. For vascular stent applications, Janus‐modified SiPSs must exhibit adequate radial force, flexibility, and recovery rate to ensure reliable fixation and deployment. Accordingly, compressive radial force analysis was conducted to quantitatively evaluate the mechanical resistance of the various Janus‐treated 3D‐printed stents.

As shown in Figure S10, all 3D‐printed stents exhibited elastic behavior after a loading–unloading cycle to 50% of their diameter, with load–distance curves demonstrating near‐complete recovery (Figure 3d). Following silica incorporation, the radial force of the SiPS reached 6.32 ± 0.35 N, which was approximately 50% higher than that of the 3D‐printed PS (4.35 ± 0.22 N). Sequential sirolimus coating and Ta S‐PIII treatment further enhanced the radial force, which increased significantly to 6.78 ± 0.27 and 6.81 ± 0.23 N, respectively. However, these increases were not statistically significant compared with those of the 3D‐printed SiPS. This modest increase in radial force can be attributed to the ultrathin spray coating of the sirolimus‐containing PLLA layer on the abluminal surface. The deposited PLLA coating provides additional mechanical reinforcement to the stent struts and partially smoothens surface irregularities or micro‐scale voids generated during the printing process, thereby alleviating localized stress concentrations and contributing to a minor improvement in mechanical performance (Figure 3e) [85, 86]. The radial force of the 3D‐printed Janus@SiPS exceeded that of commercial products ranging from 2.14 to 3.89 N [87]. Notably, while our stent features a similar strut design, its radial force is higher than that of the Abbot BVS (2.14 ± 0.03 N), suggesting that it performs at a level suitable for commercial application. All tested stents exhibited recovery rates above 90% after load release, with no statistically significant differences, indicating sufficient intravascular support for surgical implantation (Figure 3f). To further investigate mechanical performance, three‐point bending tests were subsequently performed. The load–displacement curves of the 3D‐printed stents are shown in Figure 3g. All stents demonstrated flexibility and recovered to their designed architecture without deformation, which is an essential requirement for catheter‐assisted stent deployment. Among them, the 3D‐printed Janus stent displayed the highest load value (1.04 ± 0.19 N), corresponding to the lowest flexibility, whereas the 3D‐printed PS exhibited the greatest flexibility. Based on the compressive radial test results comparing stents with and without silica, the presence of silica is anticipated to increase stiffness, which in turn reduces flexibility. After sirolimus coating, additional reduction in flexibility was observed in SRL@SiPS (0.97 ± 0.10 N). According to previous reports, polymer surface coatings can lead to decreased flexibility [87]. Moreover, PLLA, which has a higher elastic modulus than PCL, might negatively affect flexibility. However, no significant differences were observed between the SiPS, SRL@SiPS, and Janus stents, indicating that the Janus nanoengineering process did not alter the mechanical behavior of SiPS as a base material (Figure 3h). Although a slight reduction in flexibility was measured, the values were comparable to those of commercial stents—*Resolute Integrity* and *Xience PRIME* exhibit radial forces of approximately 1.14 ± 0.04 and 1.07 ± 0.04 N, respectively—demonstrating that the 3D‐printed Janus stent is suitable for cardiovascular implantation [88].

Clinical tracking and research results indicate that bare synthetic polymers such as PCL and PLLA carry a high risk of stent failure due to limited contact with the blood vessel surface and reduced endothelium regeneration efficacy. Their strongly hydrophobic surfaces and chemically inert polymeric chain groups, which lack reactive side groups, lead to poor vascular responses, thrombus formation, restenosis, and even inflammatory responses. Therefore, prior to evaluating the in vitro cellular responses, the wettability of various stent surfaces—an important surface characteristic affecting vascular tissue responses—was evaluated by measuring the contact angle of distilled water formed on the specimen surface. As illustrated in Figure 3i, the bare PS surface displayed a contact angle of 75.3° ± 3.3°, indicating relative hydrophobicity, whereas SiPS exhibited improved hydrophilicity with a contact angle of 43° ± 2.4°. This improvement reflects the hybridization of sol–gel‐derived silica, which possesses excellent wettability, into the 3D‐printed stents. After coating sirolimus‐loaded PLLA, the SRL@SiPS surface exhibited a slightly hydrophilic character, with a contact angle of 51.7° ± 1.7°. This value is lower than that of the bare PS, despite the strong hydrophobicity of sirolimus. Similar phenomena have been reported in other studies of sirolimus‐eluting stents, where variations in the surface roughness caused by the presence of the drug have been suggested to influence wettability [30, 89, 90]. The Ta@SiPS and Ta‐SRL@SiPS surfaces, which correspond to the luminal and abluminal surfaces of the 3D‐printed Ta‐SRL@SiPS, became more hydrophilic, with contact angles of 31° ± 1.4° and 33.1° ± 2.4°, respectively. These results are in good agreement with previous reports (Figure S11) [30, 91]. The low contact angles observed on both the Ta‐ion‐implanted SiPS and sirolimus‐loaded PLLA surfaces result from the high surface energy and wettability of elemental Ta on the 3D‐printed stent surface. Moreover, the increased wettability of the Ta‐ion‐implanted surface is attributed to the presence of hydrophilic functional groups (Ta─ OH), as confirmed by XPS analysis. These stent surface modifications are projected to improve synergistic bioactive functions, including the promotion of HUVEC responses on the luminal surface and inhibition of VSMC proliferation and platelet activation on the abluminal surface.

### 2.4 In Vitro Degradation Behavior of 3D‐Printed Janus‐Nanoengineered SiPSs

**FIGURE 4:**
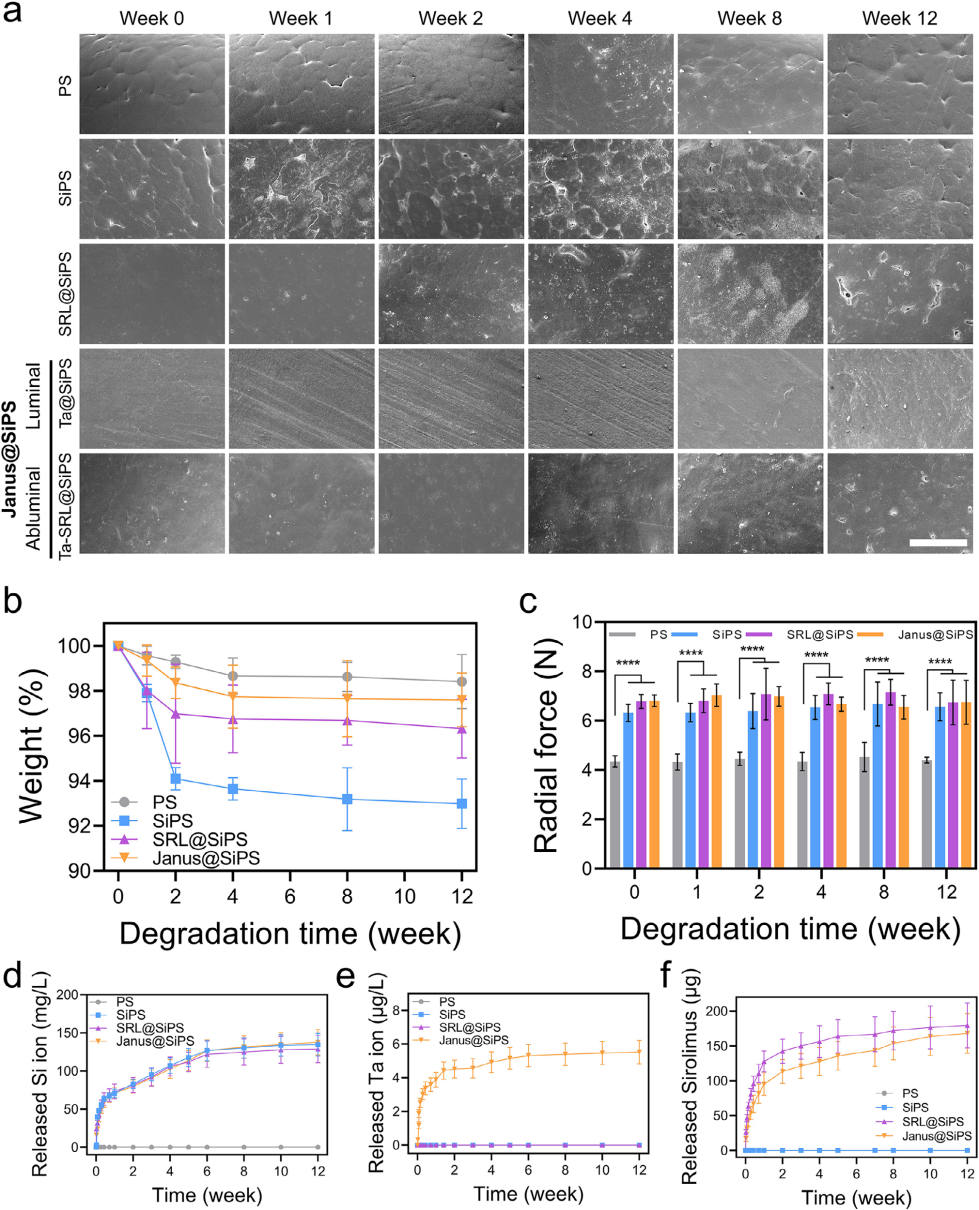
In vitro degradation and release behaviors of 3D‐printed Janus‐nanoengineered SiPSs. (a) Representative surface images obtained by FE‐SEM (scale bar: 50 µm). (b) Weight‐change ratios and (c) radial forces of 3D‐printed PS, SiPS, SRL@SiPS, and Janus@SiPS over 12 weeks of degradation (*n* = 3). (d) Accumulated release of Si ions and (e) release of Ta ions from 3D‐printed PS, SiPS, SRL@SiPS, and Janus@SiPS (*n* = 3). (f) Sirolimus release profiles (*n* = 3). Data are shown as mean ± standard deviation (SD). Normality was tested using the Shapiro–Wilk method, and one‐way ANOVA followed by Tukey's HSD post hoc analysis was applied, with significance at *p < 0.05, **p < 0.01, ***p < 0.005, and ****p < 0.001.

For applications as vascular stents, 3D‐printed BVSs must display excellent mechanical strength to support and retain narrowed blood vessels. They must also exhibit an appropriate degradation rate that parallels the rate of regeneration of injured vascular tissue. Therefore, the degradation behavior of each stent was evaluated over 12 weeks by monitoring changes in surface morphology, weight, and radial force. This duration was selected to allow sufficient time for healing of impaired peripheral vessels associated with PAD [87]. As shown in Figure 4a, the physical changes of the stents were evaluated by examining their surfaces using FE‐SEM. For the 3D‐printed PS and SiPS, surface degradation mainly occurred in the initially rough regions characterized by micropores. In particular, the SiPS boundary regions underwent deeper degradation. As the incubation progressed, a pronounced intaglio surface emerged by week 12. The SRL@SiPS, which initially possessed a smooth surface, gradually developed a rough texture with irregular cracks and fractures over time. In contrast, few voids were observed on both the luminal and abluminal surfaces of the Janus stent. Notably, elemental Ta was uniformly present on both the inner and outer surfaces of the stent, even after 12 weeks of immersion, demonstrating that the Ta S‐PIII treatment provided outstanding surface stability. (Figure S12). Following the degradation test, the PS exhibited a slight weight reduction of approximately 1.6% over 12 weeks, retaining 93% ± 1.1% of its original weight. Sol–gel‐derived silica NPs are known to undergo self‐degradation under physiological conditions. Consistent with this behavior, the SiPS showed a relatively faster weight loss during the extended degradation test [58]. In contrast, the 3D‐printed SRL@SiPS and Ta‐SRL@SiPSs exhibited almost no change in weight from their initial values (96.3% ± 1.3% and 97.6% ± 1.2%, respectively), suggesting that Janus surface modification successfully inhibited silica release (Figure 4b). Interestingly, all 3D‐printed stents exhibited similar radial force trends from weeks one to 12, comparable to their values prior to immersion (week 0). Moreover, no significant differences in radial force were observed at week 12 among the SiPS‐based stents (SiPS, SRL@SiPS, and Janus@SiPS), which may be attributed to the slow degradability of PCL even after functionalization. Although the weight decreased slightly during the first 12 weeks, the radial force of the 3D‐printed stents remained almost unchanged or displayed a gradual increase (Figure 4c). PCL degradation can occur via several mechanisms, including hydrolytic, radical‐mediated, thermal, pH‐mediated, intracellular, and enzymatic pathways [92]. Considering our experimental design, which sequentially involved 3D printing and the Ta S‐PIII process, we initially assumed that radical‐mediated and thermal degradation processes could be attributed to the degradation of the 3D‐printed Janus stent. However, given the very short exposure times to plasma and heat during the Ta S‐PIII and 3D printing processes, respectively, these mechanisms are unlikely to be the main contributors to stent degradation. Thus, we suppose that hydrolysis during the in vitro immersion test is the dominant factor affecting stent degradation. Similar trends showing decreased weight without changes in mechanical properties have also been reported in previous studies [87, 92, 93]. During the degradation stable stage, a temporary increase in mechanical properties was observed at week 10 [93]. This was attributed to the increased PCL crystallinity resulting from hydrolysis, which preferentially targets the amorphous regions. Our experimental data align with this phenomenon, as the mechanical properties of the 3D‐printed stents were maintained despite a slight decrease in weight. This is projected, given that our degradation test was conducted over 12 weeks, which falls within the stable stage. Considering that cardiovascular vessel regeneration requires approximately 12 weeks, the 3D‐printed Janus@SiPS can preserve its structural integrity. Simultaneously, it accelerates the degradation of PS to better align with the regeneration timeline of damaged blood vessels.

Subsequently, alterations in the crystalline fraction of PCL, the dominant polymeric framework of the stent, serve as a key metric for delineating degradation characteristics within the bulk matrix. Under aqueous conditions, chain scission in PCL preferentially occurs within disordered (amorphous) domains, thereby shifting the relative proportion of ordered crystalline regions. Accordingly, quantitative tracking of crystallinity is indispensable for clarifying the structural robustness and degradation pathway of PCL‐based bioresorbable vascular scaffolds (BVSs) [94, 95]. In this work, X‐ray diffraction (XRD) measurements were carried out to characterize the temporal evolution of crystallinity during degradation. An accelerated degradation protocol was implemented for 3 weeks, and independent 3D‐printed stents were analyzed at 0, 1, 2, and 3 weeks. Before assessing crystallinity, the acceleration factor relative to standard degradation conditions was determined through gravimetric mass‐loss analysis. The data demonstrated that exposure at 50°C resulted in an approximately fourfold enhancement in degradation kinetics compared with conventional 37°C conditions (Figure 4b; Figure S13) [96]. On this basis, 3 weeks under accelerated conditions were regarded as equivalent to roughly 12 weeks under physiological temperature. XRD characterization was therefore conducted at each interval to quantify changes in the crystalline fraction as degradation advanced. At the initial time point, PS, SiPS, SRL@SiPS, and Janus@SiPS displayed distinguishable baseline crystallinity values, reflecting variations introduced by silica incorporation, sirolimus/PLLA deposition, and Ta S‐PIII surface modification.

As illustrated in Figure S14, all specimens presented the characteristic diffraction reflections of semicrystalline PCL at 2*θ* ≈ 21.4°, 22°, and 23.7°, assigned to the (110), (111), and (200) planes of the orthorhombic lattice, respectively [97, 98]. Integration of the diffraction peaks enabled quantitative estimation of crystallinity, yielding initial values of 51.97% ± 1.24% (PS), 53.81% ± 2.15% (SiPS), 53.35% ± 2.15% (SRL@SiPS), and 53.03% ± 1.84% (Janus@SiPS). With increasing immersion time, PS exhibited a progressive rise to 54.17% ± 2.38% at week 3, consistent with preferential removal of amorphous segments. By contrast, SiPS showed only marginal variation (53.99% ± 2.28% at week 1 and 53.74% ± 2.08% at week 3), whereas SRL@SiPS exhibited a modest reduction to 52.25% ± 1.74% at week 3. Janus@SiPS displayed the smallest fluctuation over time (53.46% ± 2.14% at week 1 and 53.18% ± 1.57% at week 3), indicative of resistance to degradation‐associated crystalline rearrangement (Figures S14 and S15).

To substantiate the diffraction‐based findings, differential scanning calorimetry (DSC) was performed as an auxiliary thermal analysis. Whereas XRD interrogates lattice periodicity and crystalline domain organization, DSC captures bulk thermal transitions and provides quantitative information through melting enthalpy, which correlates directly with the overall crystalline fraction of the polymer network [99, 100].

As shown in Figure S16, each stent exhibited a single melting endotherm characteristic of semicrystalline PCL within approximately 60°C–70°C during the accelerated degradation period. No secondary thermal events were detected, and the melting temperature remained essentially constant with time, signifying retention of the crystalline phase architecture. The DSC profiles paralleled the crystallinity tendencies of XRD at all sampling intervals. The persistence of a single melting transition and the negligible variation in melting temperature across all groups indicate that the inherent crystalline phase of PCL maintained structural coherence under accelerated conditions. The comparatively stable degradation behavior observed in SiPS and Janus@SiPS is consistent with the limited crystallinity changes quantified from diffraction analysis, thereby supporting the robustness of the structural interpretation. The close agreement between XRD and DSC outcomes indicates that the evolution of crystallinity arises from a regulated matrix rearrangement accompanying degradation, rather than from random structural failure. These observations suggest that silica incorporation, the ultrathin SRL/PLLA layer, and Ta ion implantation jointly influenced hydrolytic degradation dynamics and subsequent crystal development within the PCL matrix, thereby preserving structural continuity throughout the degradation period [101, 102, 103, 104].

### 2.5 In Vitro Release Behavior of 3D‐Printed Janus‐Nanoengineered SiPSs

For effective therapy at the site of peripheral injury, sustained and controlled release of therapeutics is favored over rapid burst release. This is because controlled release helps maintain an optimal concentration within the therapeutic window throughout the treatment period for PAD. Therefore, in addition to assessing the degradation characteristics of 3D‐printed Janus‐treated SiPSs, the release profiles of both metal ions and sirolimus were monitored in an artificial plasma solution for 12 weeks. Similar release trends were observed throughout this period, exhibiting typical release profiles with comparable loaded and cumulative sirolimus amounts, regardless of abluminal sirolimus coating and Ta S‐PIII treatment. These results suggest that the abluminal sirolimus coating did not significantly affect the total amount of released Si ions or the macroscopic release behavior. Moreover, these results are in good agreement with the degradation data, which revealed comparable surface morphologies and radial forces among the various 3D‐printed stents (Figure 4d).

As shown in Figure S17, 3D‐printed SiPS and SRL@SiPSs presented a typical Si ion release profile characterized by an initial burst release within approximately two days. The 3D‐printed SiPS demonstrated a pronounced initial burst release, with 30.5% ions released within the first 12 h and over 49.4% released by day three, after which approximately half of the remaining Si ions were gradually released. In contrast, 3D‐printed SRL@SiPS displayed a relatively suppressed initial burst release attributed to the abluminal PLLA–sirolimus protective layer. The 3D‐printed Janus@SiPS exhibited a two‐step Si ion release profile: Initially, a burst release occurred, with less than half of the ions being released within the first 12 h, which was lower than that observed for 3D‐printed SiPS and SRL@SiPSs. This was followed by a slow and continuous release for the remainder of the monitoring period. This behavior is mainly attributed to the Janus nanosurfaces, which consist of a luminal nanolayer implanted with Ta ions on the topmost surface of SiPS and an abluminal sirolimus layer, also containing implanted Ta ions.

Importantly, the moderated initial burst release observed for Janus@SiPS is attributed to surface‐mediated diffusion control by the Janus nanosurface, whereas the subsequent long‐term Si ion release is governed predominantly by bulk degradation of the SiPS matrix. As a result, despite the distinct differences observed during the early release stage, the cumulative Si ion release profiles of the Si‐containing stents gradually converged at later time points, leading to comparable long‐term release behavior. These results indicate that the Janus nanosurface effectively modulates the early‐stage Si ion release kinetics but does not fundamentally alter the long‐term Si ion release governed by matrix degradation in the 3D‐printed stent struts. The stability of the Janus nanosurface was further evaluated by monitoring the concentration of Ta ions released under conditions identical to those used in the Si ion release test. Compared to the released Si ions, fewer Ta ions were released from the 3D‐printed Janus@SiPS. The embedded Ta ions exhibited an initial burst, followed by a gradual release, with nearly 80% of the total Ta ions released by one week. The accumulated amount of released ions was 4.92 ± 0.61 µg L^−1^ over four weeks (Figure 4e). Notably, FE‐SEM and EDS analyses revealed no significant changes in morphology or composition between the pre‐immersion state (week 0) and after 12 weeks of immersion. Surprisingly, even after this 12‐week period, Ta was still present on the surface of the 3D‐printed Janus@SiPS. The ultra‐thin, dense Ta nuclei‐implanted nanosurface layer, confined within approximately 40–80 nm from the stent surface, forms a compact and continuous Ta‐enriched region via the S‐PIII process, which is known to generate a stable passive oxide layer and enhance chemical stability in a physiological environment [33, 105]. Consistent with this, the Ta‐implanted Janus@SiPS stents exhibited degradation behavior comparable to that of SRL@SiPS, indicating that the Ta‐residing nanosurface layer does not compromise corrosion‐related stability under physiological conditions. Thus, this chemically and mechanically stable Ta‐residing layer could provide protection against clinical complications in BVSs.

Figure 4f illustrates the sirolimus release profiles from the different 3D‐printed SiPSs following a 12‐week immersion in an artificial plasma solution. Sirolimus release behavior from all 3D‐printed stents was evaluated over 12 weeks, after which no further release was observed. The 3D‐printed SRL@SiPS exhibited an initial burst release on the first day of immersion, resulting in a substantial cumulative release of sirolimus—approximately one‐third of the loaded amount. After two weeks, only approximately 20% of the residual sirolimus remained. In contrast, sirolimus from the 3D‐printed Janus@SiPS exhibited sustained release, with a relatively prolonged, near‐linear release profile. During the early phase (up to five days), the Janus@SiPS demonstrated a more prolonged release of sirolimus than SRL@SiPS. However, after one week of immersion, the sirolimus release rate significantly decreased (Figure S18). After eight weeks, most of the sirolimus loaded into the 3D‐printed SRL@SiPS had been released, whereas approximately 10% of the sirolimus was retained within the Janus nanolayer of the 3D‐printed Janus@SiPS. The extended, near‐linear release from the Janus nanosurface was mainly attributed to the incorporation of Ta ions close to the outer layer of the sirolimus–PLLA coating. Cheon et al. reported that implantation of heavy Ta ions into PLLA surfaces can induce compressive stress, which may reduce the spacing between surface PLLA chains [30]. This densification creates a more compact, metal nuclei‐implanted nanosurface that acts as an energetic physical barrier to diffuse sirolimus, which is relatively large in size (914.2 Da) [30]. Based on this complex strategy using Ta S‐PIII treatment, we predicted that the Ta S‐PIII‐treated luminal nanosurface would synergistically improve endothelial regeneration by releasing Ta and Si metal ions. In contrast, the Ta S‐PIII‐treated abluminal nanosurface could suppress SMC activity by moderating the excessive initial burst of sirolimus.

### 2.6 In Vitro Hemocompatibility of 3D‐Printed Janus‐Nanoengineered SiPSs

**FIGURE 5:**
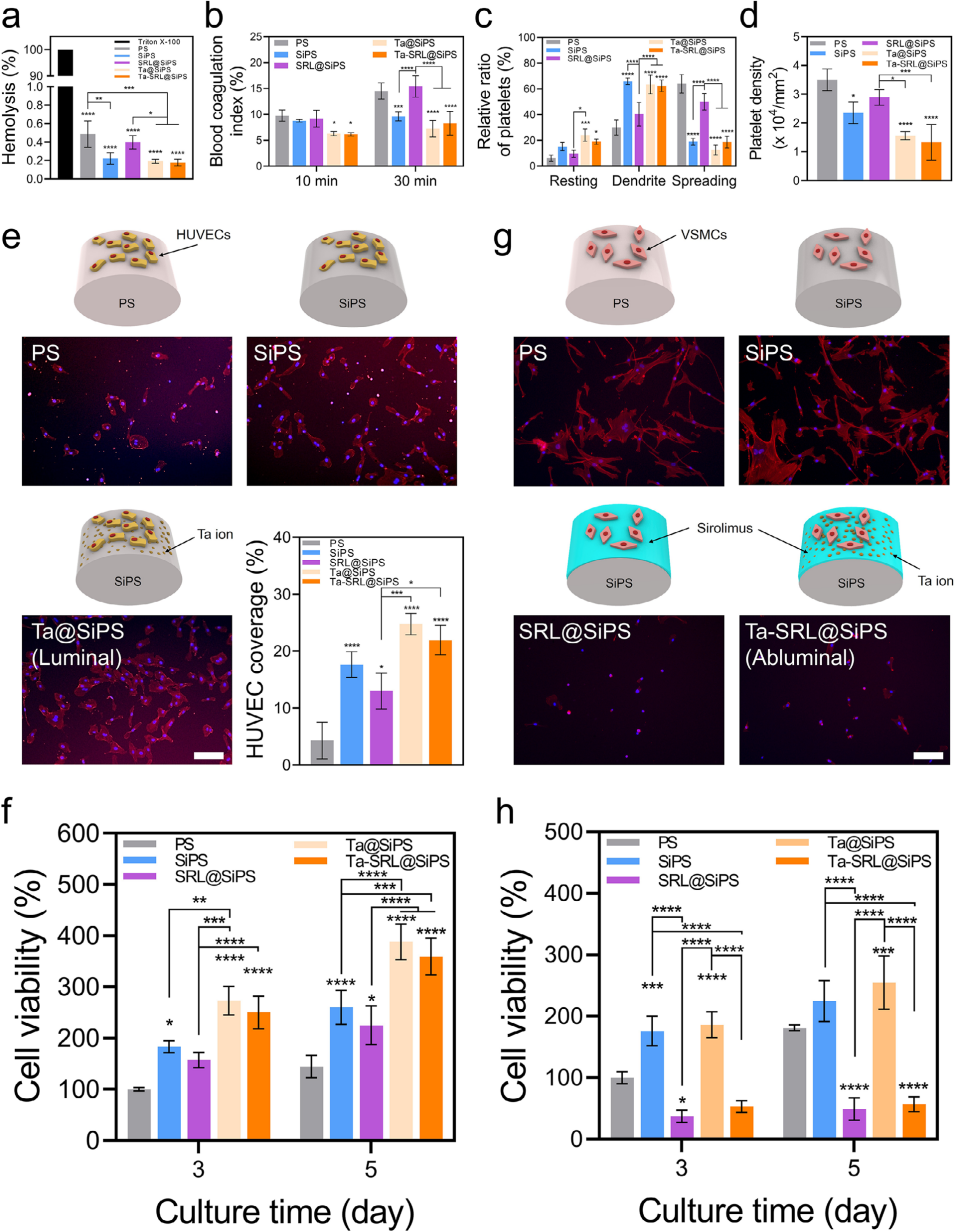
In vitro evaluation of hemocompatibility and cytocompatibility of 3D‐printed Janus‐nanoengineered SiPSs. (a) Hemolysis rates and (b) blood coagulation indices at 10 and 30 min for different 3D‐printed stents (*n* = 3). (c) Quantification of adhered platelets in resting, dendritic, and spreading states and (d) adhered platelet density on various 3D‐printed stents (*n* = 3). (e) Schematic illustrations, CLSM images, and quantitative HUVEC coverage on the luminal surfaces of the 3D‐printed stents (scale bar: 100 µm, *n* = 3). (f) HUVEC proliferation rate after three and five days of culture (*n* = 3). (g) Schematic illustrations and CLSM images of SMC adhesion on the abluminal surfaces of various 3D‐printed stents (scale bar: 100 µm). (h) SMC proliferation rate after three and five days of culture (*n* = 3). Data are shown as mean ± standard deviation (SD). Normality was tested using the Shapiro–Wilk method, and one‐way ANOVA followed by Tukey's HSD post hoc analysis was applied, with significance at *p < 0.05, **p < 0.01, ***p < 0.005, and ****p < 0.001.

Given that vascular stents are in direct contact with blood, in vitro hemocompatibility tests, including hemolysis, blood coagulation, and platelet adhesion, were conducted. The interactions between the 3D‐printed stents and erythrocytes are illustrated in Figure 5a. To quantitatively assess the hemolysis ratio, Triton X‐100 was employed as the positive control, whereas PBS was used as the negative control. The hemolysis ratios of all the 3D‐printed stents were below 0.5%, meeting the ASTM standard (<5%, F756‐2008). This indicates that these FDA‐approved PCL‐based stents exhibit excellent in vitro hemocompatibility, with hemolysis levels below the critical safety threshold for biomaterials. The hemolysis rate of the SiPS specimen (0.22% ± 0.06%) was less than half that of PS (0.49% ± 0.14%), implying that the improved surface wettability imparted by silica NPs decreased the hemolysis rate.

Following sirolimus coating, the hemolysis rate of SRL@SiPS increased again. This was attributed to the initial rapid burst release of sirolimus, which inhibited proliferating cells, as reflected in the cumulative sirolimus release profile. However, for the Ta@SiPS and Ta‐SRL@SiPS specimens, which respectively represent the luminal and abluminal surfaces of Janus@SiPS, the hemolysis rates were below 0.2%, suggesting good hemocompatibility of the Ta material [34, 106].

In the early post‐implantation phase, thrombogenicity, which is caused by intravascular coagulation and platelet activation, represents a major challenge for stents. Thus, cardiovascular stents must effectively inhibit blood coagulation and prevent thrombosis. Given that thrombogenesis involves a series of biochemical processes, such as blood coagulation and platelet aggregation, we evaluated both these antithrombotic properties in vitro [107]. The calculated blood coagulation indices of the 3D‐printed specimens, determined from UV absorbance measurements, are displayed in Figure 5b. The PS, SiPS, and SRL@SiPS surfaces presented statistically similar blood coagulation indices (∼9%), whereas the Ta@SiPS and Ta‐SRL@SiPS surfaces exhibited a 30% reduction in coagulation index after 10 min of incubation. These results suggest that Ta S‐PIII treatment significantly enhanced anticoagulation properties. Extending the incubation time to 30 min increased the coagulation indices of PS and SRL@SiPS to 15%, clearly distinguishing them from SiPS, Ta@SiPS, and Ta‐SRL@SiPS. These findings indicate that functionalizing the stent surface with silica and Ta imparted anticoagulant activity and helped suppress intravascular coagulation [108, 109, 110, 111].

Human platelet activation responses were also observed to evaluate the thrombogenicity of the stent surfaces. Platelet activation is known to induce distinctive morphological changes, typically classified into three stages reflecting increasing activation: the resting stage, which is a non‐thrombotic state with smooth discoid platelets; the dendritic stage, characterized by filopodia‐like protrusions; and the spread stage, representing severe platelet activation. Once adhered platelets become activated, they mediate inflammatory responses by releasing multiple pro‐inflammatory molecules. These molecules further promote blood coagulation and contribute to ISR and thrombosis. The 3D‐printed PS exhibited the highest platelet adhesion and spreading, owing to its hydrophobic surface that promotes platelet adhesion (Figure S19). Generally, hydrophobic surfaces hinder cell adhesion by restricting cell access to the surrounding fluid. However, they can also adsorb plasma proteins such as fibrinogen, which is a key extracellular matrix protein that binds to platelet receptors. Platelets adhered to 3D‐printed SiPS exhibited a more dendritic morphology and a 34% lower platelet density compared to those on 3D‐printed PS. This can be attributed to the hydroxyl groups on the silica NPs and indicates a lower platelet activation status. The morphology and density of platelets adhered to the 3D‐printed SRL@SiPS surface were similar to those on 3D‐printed PS, indicating that sirolimus exhibited no observable effect on platelet behavior; these results were consistent with the wettability measurements. Both the abluminal and luminal surfaces of the Ta S‐PIII‐treated Janus stent exhibited approximately 60% fewer adhered platelets, with the relative ratio of spread platelets reduced by 25% when compared to those of the 3D‐printed PS. These results support the assumption that the implanted Ta ion layer provides steric repulsion between the stent surface and adsorbed proteins, thus inhibiting platelet adhesion. Furthermore, both silicon dioxide and tantalum pentoxide are wide‐bandgap materials, with bandgaps of approximately 9 and 4.4 eV, respectively, compared to other transition metal oxides (e.g., bandgap of titanium dioxide: 3.2 eV) [112, 113, 114, 115, 116]. This wide bandgap helps prevent fibrinogen denaturation by inhibiting electron transfer from the occupied valence band of fibrinogen to the biomaterial surface. Such electron transfer can decompose fibrinogen into fibrin monomers and fibrinopeptides, subsequently leading to intraluminal thrombus formation (Figure 5c,d) [116]. Hence, the Janus nanosurface likely improves bioactive functions, including hemocompatibility and antithrombogenic properties. It may also serve as a strategic platform to minimize thrombus formation within the 3D‐printed Janus@SiPS.

### 2.7 In Vitro Cytocompatibility of 3D‐Printed Janus‐Nanoengineered SiPSs

For stent materials, cellular responses are fundamental. In particular, the promotion of cell regeneration is essential for re‐endothelialization. Although the optimal silica content was previously determined through mechanical considerations, additional in vitro evaluations were conducted to verify that the optimized composition also ensures cytocompatibility and the absence of cellular toxicity. Therefore, HUVECs were employed to evaluate the cytocompatibility of the base PS structure and the intrinsic cytotoxicity of silica. This was achieved by analyzing SiPSs with varying silica contents, given that endothelial cells constitute the intimate layer of native blood vessels. Cell adhesion on bare PS and SiPS after one day of culturing was confirmed using FE‐SEM and confocal laser scanning microscopy (CLSM) imaging (Figure S20). The results revealed that the cultured HUVECs were uniformly distributed across all specimen surfaces. Cells adhered and spread well on both bare PS and SiPS, and both the number of adhered HUVECs and their average spreading area increased with increasing silica content. These findings demonstrate that all 3D‐printed SiPS specimens supported HUVEC viability. Consequently, the highest adhesion and cell spreading were observed on 3D‐printed Si30@SiPS, as indicated by the predominance of green‐stained areas in the corresponding CLSM images. In addition to adhesion, HUVEC proliferation on each specimen was evaluated on days three and five using the CCK assay (Figure S21). HUVECs on all SiPS specimens displayed increased proliferation over time, suggesting that all the SiPS specimens were cytocompatible. Consistent with the adhesion results, the viability of HUVECs on all of the 3D‐printed SiPSs was significantly higher than that on bare PS throughout the testing period. Moreover, SiPS specimens with higher silica contents exhibited superior cytocompatibility. This enhanced cell response was attributed to the release of Si ions, which are known to promote cell adhesion and proliferation, together with the enhanced hydrophilicity resulting from silica incorporation. Additionally, the released Si ions can upregulate the vascular endothelial growth factor expression in HUVECs, thereby promoting blood vessel formation and inhibiting inflammatory reaction.

To assess the endothelium regeneration efficiency of the stent luminal surface, we investigated the morphology, surface coverage, and viability of HUVECs on the PS, SiPS, and Ta@SiPS specimens. Figure 5e displays representative fluorescence images and surface coverage of the cultured HUVECs, dual‐stained for actin filaments and nuclei, on the 3D‐printed specimen surfaces after 1 day of culture. A greater number of HUVECs adhered to the SiPS and Ta@SiPS surfaces than to PS. Regarding cell morphology, the HUVECs on the PS surface were just starting to spread with a few cellular protrusions, whereas fusiform cells were observed on the SiPS and Ta@SiPS surfaces. In particular, on Ta@SiPS, several distinct pseudopodia and actin filaments were clearly visible, confirming enhanced endothelial behavior. To assess the biological effect of drug elution from the abluminal coating, supplementary HUVEC culture studies were conducted on SRL@SiPS and Ta‐SRL@SiPS substrates, representing the outer surface of the Janus@SiPS configuration. As illustrated in Figure 5e and Figure S22, fluorescence imaging confirmed that endothelial adhesion and spreading on SRL@SiPS and Ta‐SRL@SiPS were comparable to those observed on the corresponding SiPS and Ta@SiPS surfaces. No evident decrease in cell attachment density or surface coverage was identified in the presence of sirolimus. HUVECs on SRL@SiPS and Ta‐SRL@SiPS exhibited elongated, spindle‐shaped morphologies with clearly organized actin filaments and discernible cellular extensions, closely resembling the morphology observed on SiPS and Ta@SiPS. In particular, cells cultured on Ta‐SRL@SiPS retained distinct cytoskeletal organization, indicating preserved endothelial characteristics despite incorporation of the sirolimus‐containing coating. These findings demonstrate that sirolimus released from the abluminal surface did not adversely affect endothelial adhesion or morphology under the current experimental conditions [17, 30]. Quantitative analysis of cell spreading further supported these observations. The PS surface displayed the lowest endothelial coverage (4.3% ± 3.2%). In comparison, SiPS exhibited increased coverage (17.7% ± 2.3%), while SRL@SiPS showed a similar level of spreading (13% ± 3.2%), indicating minimal influence of sirolimus on endothelial expansion. The Ta@SiPS surface demonstrated the greatest coverage (24.7% ± 1.9%), and this favorable response was largely preserved on Ta‐SRL@SiPS (22% ± 2.6%). Endothelial proliferation was subsequently examined on days 3 and 5 using the CCK‐8 assay. As shown in Figure 5f, absorbance at 450 nm increased from day 3 to day 5 for all specimens, reflecting sustained HUVEC growth on the 3D‐printed BVS substrates. At day 3, SiPS and SRL@SiPS exhibited enhanced viability (183% ± 11.7% and 157% ± 14.8%, respectively) relative to PS (100% ± 3%), whereas Ta@SiPS and Ta‐SRL@SiPS showed markedly higher metabolic activity (273% ± 27.8% and 250% ± 31.7%, respectively). By day 5, proliferation further increased across all groups. SiPS and SRL@SiPS reached 260% ± 33.3% and 224.9% ± 37.9%, respectively, exceeding PS (144.4% ± 21.8%). Notably, Ta@SiPS exhibited the highest viability (388% ± 35%), followed by Ta‐SRL@SiPS (359.2% ± 36.2%). Overall, endothelial viability on both Ta‐modified surfaces was significantly greater than that on PS and SiPS, with Ta@SiPS showing approximately a 2.7‐fold increase compared with PS at day 5.

Consistent with the tendency observed in Figure S21, incorporation of silica nanoparticles into the PCL matrix promoted endothelial proliferation relative to bare PS, and subsequent Ta ion modification further strengthened this response. This enhanced cellular activity is attributed to the combined presence of silica and Ta species on the luminal surface of the 3D‐printed Janus stent, which together support endothelial growth and are expected to facilitate accelerated endothelialization.

Si is known to effectively promote cell adhesion and proliferation, owing to its high hydrophilicity. Consequently, new blood vessel tissue could regenerate more efficiently at the boundary between the damaged vessel and stent when the luminal surface incorporates silica [117]. Strongly embedded Ta ions in SiPS generate reactive oxygen functionalities by breaking polymeric chains. Moreover, the increased wettability of the 3D‐printed stent surface, resulting from the hydrophilic Ta─OH groups, provides favorable cell–surface interactions. Overall, the findings on cell adhesion and viability indicate that the Ta@SiPS surface demonstrates superior biocompatibility compared with that of PS. This improvement is attributed to the combined effects of the Ta‐modified stent surface and the release of Ta and Si ions. Additionally, the Ta@SiPS surface can support the healing of damaged blood vessels without inducing long‐term side effects on cell proliferation, such as cytotoxicity, which can be associated with nanomaterials.

While rapid endothelialization is essential, suppression of SMC overproliferation is equally critical. NIH‐driven excessive SMC proliferation remains a principal factor contributing to stent restenosis. To examine SMC responses, adhesion and morphology were observed on the abluminal surfaces of the 3D‐printed stents, while Ta@SiPS, corresponding to the luminal surface of Janus@SiPS, was included (Figure 5g; Figure S23). After 24 h of incubation, VSMCs on PS and SiPS adhered readily and displayed elongated, well‐spread morphologies. In contrast, markedly fewer attached cells were observed on the sirolimus‐coated stents (SRL@SiPS and Ta‐SRL@SiPS), indicating effective suppression of VSMC expansion by sirolimus. The Ta@SiPS surface, however, supported cell attachment and spreading. Quantitative evaluation of surface coverage corroborated these observations (Figure S24). PS, SiPS, and Ta@SiPS exhibited comparatively high coverage ratios (15.7% ± 3.2%, 21.4% ± 3.6%, and 23.3% ± 4.7%, respectively). By comparison, SRL@SiPS and Ta‐SRL@SiPS demonstrated substantially lower values (2.3% ± 1.2% and 3% ± 1.4%). These findings confirm that sirolimus effectively restricts VSMC adhesion and overproliferation on the abluminal surface, even when combined with the cell‐adhesive Ta‐implanted surface. VSMC metabolic activity was further assessed on days 3 and 5 (Figure 5h). Sirolimus‐coated surfaces exhibited a significantly reduced proliferation rate. In contrast, SiPS showed markedly elevated VSMC viability at both time points (176.2% ± 24% and 224.9% ± 33.3%, respectively), suggesting an increased propensity for neointimal formation. A similar tendency was observed for Ta@SiPS (186.2% ± 21% and 254.9% ± 43.3% on days 3 and 5, respectively), indicating that Ta incorporation alone does not inhibit SMC proliferation. On days 3 and 5, SRL@SiPS (37.3% ±10% and 49% ± 18.2%) and Ta‐SRL@SiPS (52.9% ± 9.6% and 56.9% ± 12.1%) exhibited pronounced suppression of VSMC growth compared with PS (100% ± 9.6% and 181.2% ± 4.8%), consistent with the adhesion analysis. Although the proliferation rate on Ta‐SRL@SiPS was slightly higher than that on SRL@SiPS at both time points, the difference was not statistically significant. Overall, the sirolimus‐loaded surface can inhibit VSMC proliferation, whereas the implanted Ta ions have little impact on VSMC proliferation. Notably, the Ta S‐PIII‐treated Janus layer not only improved HUVEC–surface interactions on the luminal surface but also preserved the inhibitory effect of sirolimus on VSMC function. Although the Janus surface nanoengineering process required only a few tens of seconds, it enabled extensive Ta ion implantation into the SiPS at significantly high doses. This approach, in turn, facilitated the formation of asymmetric surface characteristics on both the luminal and abluminal surfaces of the 3D‐printed Janus stent.

### 2.8 In Vivo Assessment of 3D‐Printed Janus@SiPSs in the Porcine Popliteal Artery

**FIGURE 6:**
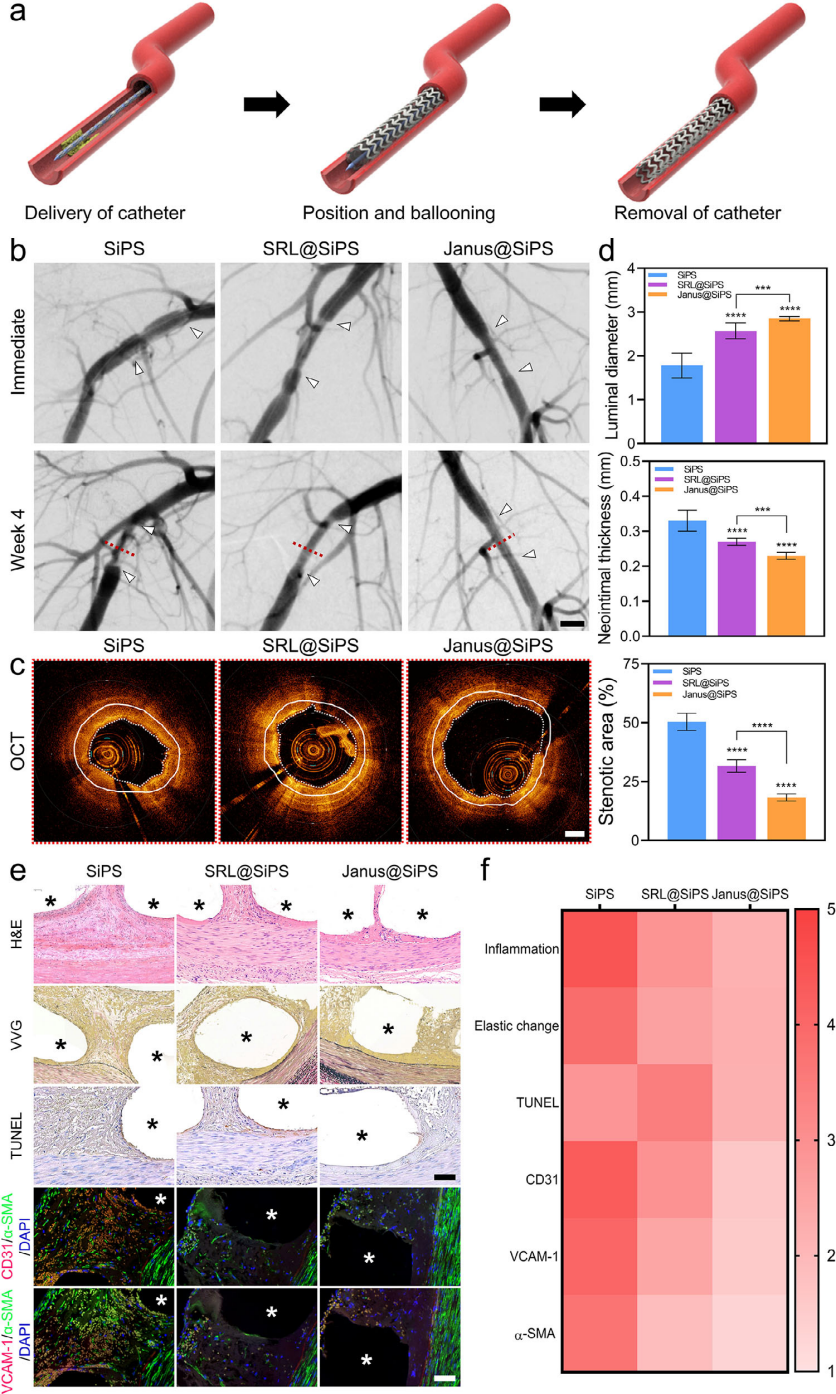
Angiographic, OCT, and histological evaluations at four weeks after stent implantation in the porcine popliteal artery. (a) Schematic illustration of the implantation procedure for 3D‐printed BVSs, showing sequential steps of catheter delivery, positioning with balloon expansion, and catheter removal after stent deployment. (b) Immediately and 4 weeks after angiography (scale bar: 6 mm), showing the stented popliteal artery (arrowheads), and (c) follow‐up OCT images (scale bar: 500 µm) demonstrating luminal patency and neointimal remodeling, where the white solid line delineates the original luminal area and the white dashed line outlines the neointimal area. (d) The radiographic evaluation showed changes of luminal diameter in angiography, neointima thickness, and percentages of stenotic area in OCT analysis (*n* = 3). (e) Representative H&E, VVG, TUNEL‐stained microscopic images around the stent strut (black or white stars) and CD31‐, VCAM‐1, and α‐SMA immunofluorescence images of the study groups. (f) Heatmap showing the degrees of the inflammatory cell infiltration, elastic lamina change, TUNEL‐, CD31‐, VCAM‐1, and α‐SMA‐coverage deposition in the study groups. Data are shown as mean ± standard deviation (SD). Normality was tested using the Shapiro–Wilk method. For non‐normally distributed data, statistical analysis was performed using the Kruskal–Wallis *H*‐test followed by pairwise comparisons with the Mann–Whitney *U*‐test, with significance at *p < 0.05, **p < 0.01, ***p < 0.005, and ****p < 0.001.

An in vivo porcine popliteal artery model was employed to evaluate the therapeutic efficacy and biological safety of the 3D‐printed Janus@SiPS under physiological hemodynamic conditions. Prior to implantation, the stents were crimped onto a balloon catheter and introduced to the targeted popliteal artery through standard endovascular access (Movie S1). As illustrated in Figure 6a, stent deployment comprised three sequential steps: (i) insertion and navigation of the catheter to the target site, (ii) precise positioning followed by balloon inflation for complete stent deployment, and (iii) withdrawal of the catheter system, leaving the expanded stent apposed to the arterial wall. This workflow reflects conventional stent‐assisted angioplasty used in clinical practice and enables reliable assessment of device performance, including deployment behavior, arterial wall apposition, and early healing responses.

Stent placement was technically successful in all pigs, with no procedure‐related complications, and all animals survived until the end of the study. Follow‐up angiography revealed differences in luminal narrowing at the stented popliteal artery among the study groups, without adverse events such as endoleak, migration, fracture, or occlusion. The SiPS group exhibited irregular luminal narrowing, caused by thrombosis and neointimal formation at the stented popliteal artery. Comparatively, stent patency in the SRL@SiPS and Janus@SiPS groups was relatively well preserved at four weeks post‐implantation. Notably, the mean luminal diameter was significantly greater in the Janus@SiPS group than in the SiPS and SRL@SiPS groups (Figure 6b). Consistent with these results, follow‐up optical coherence tomography (OCT) confirmed similar stent patency trends (Figure 6c). The mean neointima thickness and percentage of stenotic area were significantly lower in the Janus@SiPS group compared with those in the SiPS and SRL@SiPS groups (Figure 6d).

Mechanical property‐related factors may induce flow disturbances and heterogeneous wall shear stress distributions caused by stiffness mismatches between the BVS and surrounding vasculature after implantation [118]. In this study, silica‐applied SiPSs maintained improved luminal patency without evidence of elastic recoil for four weeks in vivo. The incorporation of silica has been shown to significantly enhance the mechanical properties of the stent, thereby representing an important strategy for vascular applications of BVSs, which inherently provide weak mechanical support. In addition, the laser cutting process used to fabricate balloon‐expandable BVSs can induce microcracks within the laser‐affected zone. These defects, along with irregular deformation during crimping, may contribute to mechanical failure after stent deployment [119, 120, 121]. In this study, the effective interaction between sol–gel‐derived silica NPs and PCL enhanced the compressive properties and structural integrity of the 3D‐printed stents, maintaining stent patency for four weeks without any stent failure during implantation.

Notably, while Janus treatment enhanced stent mechanical properties, extrusion through a 200 µm‐diameter nozzle during 3D printing limited strut thickness to approximately 200–280 µm. Previous studies have demonstrated that thick or bulky struts (∼150 µm), such as those using commercially available Absorb BVS (Abbott) can impair re‐endothelialization in polymeric biodegradable stents. This may promote inflammatory cell adhesion and increase the risk of late thrombosis [122]. Thick or bulky struts have been recognized as a major contributor to the higher incidence of scaffold thrombosis and myocardial infarction with early‐generation BVSs compared to second‐generation DESs, as shown in a meta‐analysis of randomized controlled trials. This ultimately led to the market withdrawal of these devices for the treatment of atherosclerosis [123, 124]. Notably, Abbott's Esprit BTK system, featuring newly developed thinner struts (100–120 µm), has emerged as a safe and promising strategy for the treatment of PAD, offering a safer alternative to early‐generation BVSs used in the treatment of coronary artery disease [125]. In this study, the nanoengineering approach combining Ta ion implantation and sirolimus loading demonstrated superior stent patency. This was achieved despite the relatively thick stent struts (200–250 µm), which could theoretically impair re‐endothelialization. Although inadequate wall apposition remains a concern due to the lack of radiopaque markers or materials in the SiPS, which are critical for precise vascular stent placement, all stent–wall appositions in this study were successfully corrected using OCT imaging. However, enhanced radiological visibility will be important for future clinical applications. Therefore, while further studies optimizing strut dimensions and refining nanoengineering strategies for SiPSs are warranted, the current Janus@SiPS maintained enhanced patency for four weeks post‐implantation without any stent‐related complications.

The histological findings are summarized in Tables S14 and S15, with representative examples shown in Figure 6e and Figure S26. The mean degrees of inflammatory cell infiltration, elastic lamina change, and TUNEL‐, CD31‐, VCAM‐1‐, and α‐SMA‐positive cell coverage differed significantly among the study groups. In particular, these parameters were significantly lower in the SRL@SiPS and Janus@SiPS groups than in the SiPS group. Furthermore, the mean degrees of TUNEL‐, CD31‐, VCAM‐1‐, and α‐SMA‐positive cell coverage in the Janus@SiPS group were significantly lower than those in the SRL@SiPS group. However, the mean degree of TUNEL‐positive staining was significantly higher in the SRL@SiPS group than in the SiPS and Janus@SiPS groups (Figure 6f; Figures S29–S34).

These results indicate that the SRL@SiPS and Janus@SiPS groups effectively prevented ISR by controlling excessive neointima formation and SMC migration in the stented popliteal artery. Currently, sirolimus is recognized as a promising cardiovascular drug, owing to its potent immunosuppressive and antiproliferative properties [126, 127]. As a macrocyclic lactone, it regulates SMC migration, myofibroblast proliferation, and collagen synthesis, thereby inhibiting all phases of ISR [63, 128]. Nevertheless, DESs with sirolimus have limitations associated with delayed endothelialization and hypersensitivity reactions related to the initial burst, depending on the drug dose and release kinetics. These effects result in drug‐induced cytotoxicity, which is consistent with our finding of increased TUNEL‐positive staining in the SRL@SiPS group [129, 130, 131]. These limitations may trigger adverse arterial responses and increase the risk of in‐stent thrombosis [132]. In the current study, the selective coating of sirolimus on the abluminal surface of SiPS enabled localized drug delivery, whereas the dual‐coated Ta‐SRL@SiPS, fabricated using the simple S‐PIII technique, exhibited more controlled and sustained sirolimus release kinetics than the SRL@SiPS group. Therefore, comparatively, the Janus@SiPS group exhibited significantly reduced TUNEL‐positive staining—an indicator of arterial wall cell necrosis induced by drug cytotoxicity—in the neostructure formed near the stent strut. In addition, Ta, which is a highly biocompatible metal ion, was incorporated into sirolimus‐coated SiPS to achieve uniform dispersion at an optimal depth, thereby enhancing biological performance within blood vessels [133]. Ta oxides enhance hemocompatibility by inhibiting charge transfer from fibrinogen, thereby limiting thrombus formation [134]. Moreover, fine Ta or Ta oxide particles may be gradually released, partially dissolved, and subsequently cleared through normal circulation, contributing to the application of Ta on vascular stents [135]. Most Ta‐ion‐based surface modification strategies have mainly aimed to promote re‐endothelialization by improving platelet adhesion and fibrinogen adsorption, while reducing thrombogenicity. However, these strategies offer limited control over VSMC activity, which plays a key role in vascular disease progression [34, 136, 137, 138]. In this study, the pristine SiPS group demonstrated pronounced VSMC migration and excessive neointimal formation accompanied by increased CD31‐positive structures (Figure S27) and VCAM‐1 which is an indicator of inflammatory and monocyte activity (Figure S28). These expressions were not restricted to the luminal surface but were distributed throughout the thickened neointima, suggesting pathological neoangiogenesis (vasa vasorum proliferation) or infiltration of inflammation‐associated endothelial progenitor cells within the neointimal region [139]. Therefore, this may partially reflect intra‐neointimal neovascularization rather than functional luminal endothelial recovery. Meanwhile, the Janus@SiPS group, benefitting from the synergistic effects of sirolimus and Ta ions, exhibited markedly reduced neointimal formation and VSMC migration, while maintaining a well‐preserved tunica media. Despite the promising results of multifunctional nanoengineered stents with Ta and sirolimus, the short four‐week follow‐up period in this study warrants further investigation into late‐stage inflammation and thrombosis. The rate of degradation is closely linked to the degree of inflammatory response [140]. Therefore, long‐term follow‐up studies exceeding 30 months are essential to evaluate arterial remodeling and morphological restoration during the transition from late inflammation to vascular repair [141, 142]. Nevertheless, although further long‐term studies are required, the present findings demonstrate that well‐preserved stent patency and stable vascular remodeling were achieved using the 3D‐printed SiPS with Janus functionalization.

## 3 Conclusion

In summary, our findings reveal a theragenerative, personalized, and biodegradable DES approach that enhances hemocompatibility while reducing thrombogenicity in the treatment of PAD. This approach was achieved by 3D‐printing stent struts from a silica‐incorporated polymer stent (SiPS) formulation containing silica NPs, followed by Ta S‐PIII treatment of the stent surface to create asymmetric (Janus) surface properties. Homogeneous SiPS stents were successfully 3D‐printed with excellent shape fidelity and printability, where silica nanoparticles enhanced the mechanical properties of PCL and promoted HUVEC adhesion and proliferation. Ta S‐PIII enabled the formation of stable Ta nanolayers on both the luminal and abluminal surfaces, improving surface hydrophobicity, modulating degradation, and controlling the release of Si ions and sirolimus while imparting hemocompatible and antithrombogenic properties to the luminal surface. As a result, the Janus‐nanoengineered SiPS stent suppressed VSMC proliferation and neointima formation while maintaining luminal patency and enhanced mechanical performance in vivo. Furthermore, the incorporation of sirolimus and Ta within the SiPS matrix significantly inhibited VSMC migration and prevented excessive neointima formation in the porcine popliteal artery. Although long‐term follow‐up studies are required to evaluate late inflammatory and thrombotic responses during in vivo degradation, we propose that this multifunctional theragenerative Janus@SiPS platform holds strong therapeutic potential for preventing ISR in PAD. Moreover, the 3D‐printed Janus stent, developed through a combination of 3D printing and Janus nanoengineering, offers a promising avenue for future clinical applications in PAD.

## 4 Materials and Methods

### 4.1 Synthesis of SiPS With Silica

To prepare a 10 w/v% polymer solution, poly(ε‐caprolactone) (PCL; *Mn* = 45 000, Sigma Aldrich, USA) was first dissolved in dichloromethane (DCM; Sigma Aldrich, USA). The silica precursor was prepared by combining tetramethylorthosilane (TMOS; Sigma Aldrich, USA), deionized water (DW), hydrochloric acid (Dae‐Jung, Republic of Korea), and DCM in a volume ratio of 2.5:0.5:0.01:3 (Figure 1) [58]. Following a 30 min stirring period to form a uniform sol, silica sol at varying concentrations (0–30 wt.%) was incorporated into the PCL solution. The mixture was then dried on a plate for 24 h, and the resulting dried solid samples were cut into small chips for stent 3D‐printing.

### 4.2 Fabrication of 3D‐Printed SiPSs

The functionalized PSs were fabricated using a 3D stent printing system (Plancklab, Republic of Korea), as depicted in Figure 1. Each stent strut unit cell was repeated seven times in the longitudinal direction and six to eight times circumferentially. Three linking bridges connected the adjacent unit cells. The rotation direction coordinates of the designed structure (*x*, *y*, and *z*), were converted into G‐code and transmitted to the 3D printer through the instrument software. The printing system, which comprised a translational stage with three axes (*x‐Ɵ‐z*), dispenser, nozzle, compression/heat controller, and software system, operated according to the input G‐code. SiPS chips were melted in a heating dispenser at 100°C for 20 min, while the nozzle heater temperature was maintained at 110°C. After the PCL was melted, a steady air pressure of 300–350 kPa was applied to the dispenser to extrude the strand through a 200 µm nozzle. Rectangular specimens measuring 10 × 10 × 2 mm were also printed using a 3D‐bioprinter (4D6, Rokit, Republic of Korea) for physicochemical testing. The printing conditions were set at 110°C–130°C with a head speed of 5 mm s^−1^.

### 4.3 Nanoengineered Janus Treatment of 3D‐Printed SiPSs

To prepare the ultrasonic spraying solution, poly‐*L*‐lactic acid granules (PLLA; *Mn* = 100 000, Pureco, Republic of Korea) were heated in a furnace at 200°C and then rapidly cooled. The heat‐treated PLLA was dissolved in 1,4‐dioxane (Dae‐jung, Republic of Korea) to obtain a 1.5 w/v% solution containing sirolimus (LC laboratory, USA) at a concentration of 33 wt.%. In the employed 3D stent printing system, sirolimus‐loaded PLLA was applied to the abluminal surface of the 3D‐printed stents using an ultrasonic spray coating nozzle following printing of the functionalized PSs. The spraying parameters were configured with a spray concentration of 100 µg mL^−1^ and a rotational speed of 30 rpm. The coated stents were then dried at 4°C. After coating, the abluminal coated stents were removed from the *Ɵ*‐axis mandrel of the 3D‐printer, cleaned with ethanol, and exposed to UV light for 10 min prior to Ta S‐PIII treatment.

A DC magnetron sputter gun housing (Ultech, Republic of Korea) was equipped with a Ta target measuring 75 mm (diameter) × 5 mm (thickness) (purity 99.99%, Kojundo Chemical Lab, Japan). Prior to S‐PIII treatment, the vacuum chamber was evacuated to a pressure of 5 × 10^4^ Pa using both rotary and diffusion pumps. For sample preparation, the Ta target was positioned parallel to the length of the printed stents, which were secured to a stainless‐steel plate. A negative voltage of 2000 V was applied for 10 s under an Ar gas pressure of 7 mTorr. A target current of 50 mA was applied without any additional heating. The same procedure was performed on all 3D‐printed samples to generate nanoengineered Janus surfaces.

### 4.4 Characterization of 3D‐Printed SiPSs

The size and distribution of silica NPs in PCL were evaluated using dynamic light scattering (DLS; Zetasizer Nano ZS, Malvern Instruments, UK). Measurements were performed using a 633 nm laser at a scattering angle of 173° and a temperature of 25°C. The reported data represent the averaging of five independent measurements. The size and chemical composition of silica in PCL were further examined using transmission electron microscopy (TEM; JEM‐2100F, JEOL, Japan) coupled with EDS at an acceleration voltage of 200 kV. TEM samples were prepared by fragmenting the hydrogels into granules, diluting them with DW, and depositing the suspension onto a holey carbon grid (Quantifoil R1.2/1.3, 200 mesh Cu, Structure Probe, USA). The samples were then dried under vacuum for 24 h prior to observation.

The chemical bonds present in the synthesized SiPS were analyzed using Fourier‐transform infrared (FT‐IR) spectroscopy (Cary 630 FTIR, Agilent, USA). FT‐IR spectra were recorded in the range of 400–400 cm^−1^ for the bare PS, sol–gel‐derived silica, and SiPSs of varying silica concentrations. The thermal properties of the synthesized SiPSs, as a function of silica content, were evaluated through differential scanning calorimetry (DSC; PerkinElmer DSC 4000, USA) and thermogravimetric analysis (TGA; TGA2, Mettler‐Toledo, Germany). For DSC measurements, samples (2–3 mg) were sealed in aluminum pans and heated from 30°C to 100°C at a rate of 10°C min^−1^ under an argon atmosphere. TGA measurements were performed at the same heating rate (10°C min^−1^) over a temperature range of 30°C–900°C under a nitrogen atmosphere (flow rate of 50 mL min^−1^) to evaluate the thermal stability of the samples (∼5 mg).

The microstructural features of the 3D‐printed SiPSs were investigated using field‐emission scanning electron microscopy (FE‐SEM; S‐4800, HITACHI, Japan) for surface and cross‐sectional observations, coupled with EDS for elemental mapping. Both the surface and cross‐sectional morphologies of the bare PS and SiPSs were analyzed.

### 4.5 Characterization of 3D‐Printed Janus‐Nanoengineered SiPSs

To assess the surface composition of the specimens, cross‐sections were prepared using a focused ion beam (FIB; Helios 650, FEI, USA). Prior to milling, the specimens were coated with Pt using a Pt coater (108 Auto Sputter, Cressington, UK) at a current of 20 mA for 60 s. FIB milling was conducted at an acceleration voltage of 30 kV, followed by a final cleaning step at 2 kV using a 39 pA beam current. Cross‐sectional samples were examined using TEM coupled with EDS at 200 kV. The chemical composition and presence of Janus‐treated layers were analyzed using X‐ray photoelectron spectroscopy (XPS; Axis Supra, Kratos, UK). The hydrophilicity of the samples was evaluated by measuring the contact angle of DW droplets on their surfaces using a contact angle analyzer (Phoenix 300, SEO, Republic of Korea).

Mechanical tests were performed to evaluate the radial force, flexibility, and recovery rate of the 3D‐printed stents using a universal testing machine (UTM; MINOS‐005, MTDI, Republic of Korea). The radial force was measured during a loading–unloading compression test in which the diameter was reduced by 50% at a rate of 10 mm min^−1^. Flexibility was assessed using a three‐point bending test carried out at 10 mm min^−1^ in accordance with ASTM F2606‐09 for vascular stents. The span length of the testing apparatus was 11 mm, and the maximum deflection was set to 2.2 mm. For the recovery rate experiment, the stents were compressed to 50% of their original diameter, and the diameter was remeasured after 6 h using Equation (1):

(1) $$\mathrm{Recovery}\;\mathrm{rate}\;\left(\%\right)=\frac{D_{a}}{D_{o}}\;\times \;100$$

where *D_o_* is the original stent diameter and *D_a_* is the stent diameter 6 h after compression by 50%.

### 4.6 In Vitro Degradation and Release Behavior Study

An in vitro degradation study was conducted to evaluate changes in surface morphology, weight, and mechanical properties of the 3D‐printed stents. Stents with dimensions of 3 mm (diameter) × 18 mm (length) were fabricated using 3D printing technology. The stents were submerged in Dulbecco's phosphate‐buffered saline (DPBS; Welgene, Republic of Korea) for 1, 2, 4, 8, and 12 weeks and subsequently rinsed with DW. After overnight freeze‐drying, the specimens were used to determine the percentage weight loss using Equation (2):

(2) $$\mathrm{Comparative}\;\mathrm{weight}\;\left(\%\right)=\frac{W_{d}}{W_{i}}\;\times \;100$$

where *W_i_* represents the initial weight of the stent, and *W_d_* denotes the weight of the stent after freeze‐drying at each time point. The dried specimens were subsequently evaluated for radial force using a UTM and for surface morphology using FE‐SEM.

To further assess degradation changes in the polymer matrix within a shortened timeframe, an accelerated degradation study was additionally performed. The stents were immersed in DPBS at an elevated temperature (50°C) for up to 3 weeks, corresponding to the degradation behavior observed up to 12 weeks under standard conditions. Samples were collected at 0, 1, 2, and 3 weeks, rinsed thoroughly with DW, and freeze‐dried prior to further characterization.

The crystallinity evolution of the PCL matrix during accelerated degradation was analyzed by X‐ray diffraction (XRD; miniflex 600, Rigaku, Japan). XRD patterns were recorded using an X‐ray diffractometer equipped with a Cu Kα radiation source (*λ* = 1.5406 Å), operated at 40 kV and 30 mA. Diffraction data were collected over a 2*θ* range of 10°–40° at a scanning rate of 2° min^−1^. The degree of crystallinity was calculated by integrating the crystalline peak area relative to the total diffraction area after background subtraction. Differential scanning calorimetry (DSC; DSC 300, NETZSCH, Germany) was additionally employed to complement the XRD analysis and evaluate bulk crystallinity changes. DSC measurements were performed using a DSC instrument (model and manufacturer) under a nitrogen atmosphere. Samples were heated from 0°C to 100°C at a heating rate of 10°C min^−1^. The melting enthalpy (Δ*H*_m_) was obtained from the endothermic melting peak, and the degree of crystallinity was calculated using the theoretical melting enthalpy of 100% crystalline PCL.

To investigate the drug release characteristics, the 3D‐printed stents were immersed in DPBS (3 mL) containing 0.05% Tween‐20 at 37°C for 12 weeks. At regular intervals, the medium containing the released sirolimus was collected and filtered through 0.2 µm filters. The filtered medium was then combined with anhydrous methanol, and the optical density of the resulting filtered solution was measured using a Nanodrop spectrophotometer (Nanodrop, Thermo Fisher, USA) at a wavelength of 278 nm.

The 3D‐printed stents were submerged in DPBS (3 mL) for 12 weeks. To verify the initial release of Ta and Si ions, samples were collected at 4 and 12 h, after which the collection intervals were extended to one, two, three, four, and five days, followed by one, two, three, four, five, six, eight, 10, and 12 weeks. The ion concentrations were determined using an inductively coupled plasma mass spectrometer (ICP‐MS; Thermo Fisher, USA), after which the collected solution was replaced with fresh DPBS (3 mL).

### 4.7 In Vitro Hemocompatibility Evaluation

Hemolysis, platelet adhesion, and whole blood coagulation analyses were performed to evaluate the in vitro hemocompatibility of the 3D‐printed stents. Human blood samples were obtained from healthy adult volunteers by venipuncture, and 3.8 wt.% citrate acid was added as an anticoagulant. The degree of hemolysis was compared using red blood cells (RBCs). Various 3D‐printed specimens were placed in tubes containing normal saline (5 mL) and diluted whole blood (0.1 mL). The tubes were incubated in a 37°C water bath for 1 h. A combination of saline solution (5 mL) and whole blood (0.1 mL) served as the negative control, whereas a mixture of DW (5 mL) and whole blood (0.1 mL) was used as the positive control. The samples were subsequently centrifuged for 5 min at 3000 rpm, and the absorbance of the resulting supernatant was measured at a wavelength of 545 nm using a microplate reader (Synergy H1 Hybrid Multi‐Mode Reader, BioTek, USA). The percentage hemolysis was calculated using Equation (3):

(3) $$\mathrm{Hemolysis}\;\left(\%\right)=\frac{OD_{t}\;-\;OD_{nc}}{OD_{pc}\;-\;OD_{nc}}\;\times \;100$$

here, *OD_t_* is the sample optical density, *OD_nc_* is the optical density of the negative control, and *OD_pc_* is the optical density of the positive control.

The blood coagulation test was performed by depositing the citrated whole blood onto the specimen surface, followed by the addition of a coagulation reagent comprising calcium chloride solution (0.2 mol L^−1^). After incubation at 37°C for 10 and 30 min, the specimens were gently rinsed in DW, and then vigorously shaken for 1 min to obtain a homogeneous solution. The whole blood coagulation index was determined by measuring the absorbance of the resulting solution at a wavelength of 545 nm using Equation (4):

(4) $$\mathrm{Blood}\;\mathrm{coagulation}\;\mathrm{index}\;\left(\%\right)=\left(1\;-\;\frac{A_{s}}{A_{w}}\right)\;\times \;100$$

where *A_s_* represents the absorbance of anticoagulated whole blood in contact with the specimen and *A_w_* represents the absorbance of DW.

The platelet adhesion tests were carried out with platelet‐rich plasma (PRP) obtained by centrifuging citrated whole blood at 1500 rpm for 15 min. After removing the supernatant, the PRP was applied to the 3D‐printed specimens in fresh conical tubes and incubated for 1 h at 37°C. Free platelets were removed by washing with DPBS, and the adherent platelets were fixed at room temperature for 20 min in glutaraldehyde (2.5%), followed by dehydration in a graded ethanol series from 50% to 100% ethanol for 10 min. The samples were subsequently analyzed using FE‐SEM.

### 4.8 In Vitro Cytocompatibility Evaluation

For the evaluation of cell adhesion, coverage, and viability, human umbilical vein endothelial cells (HUVECs; LONZA, C2519A, Switzerland) and vascular smooth muscle cells (VSMCs; LONZA, CC‐2583, Switzerland) were cultivated in endothelial cell basal medium‐2 (EBM‐2; LONZA, Switzerland) and smooth muscle cell growth basal medium (SmBM; LONZA, Switzerland), respectively, in an incubator with 5% CO_2_ at 37°C. HUVECs were seeded at a density of 3 × 10^4^ cells mL^−1^ to assess cell adhesion and coverage, and at 2 × 10^4^ cells mL^−1^ to evaluate cell viability on PS, SiPS, SRL@SiPS, Ta@SiPS, and Ta‐SRL@SiPS samples. VSMCs were tested using the same method used for the HUVECs with PS, SiPS, SRL@SiPS, Ta@SiPS, and Ta‐SRL@SiPS samples. Prior to confocal laser scanning microscopy (CLSM) examination, cells cultured for one day were fixed in 4% paraformaldehyde for 10 min and subsequently washed twice with PBS. To block nonspecific binding sites, the remaining cells were treated for 5 min with 0.1% Triton X‐100 and 1% bovine serum albumin (Sigma Aldrich, USA), followed by staining of F‐actin and cell nuclei with phalloidin and 4′, 6‐diamidino‐2‐phenylindole (DAPI), respectively. Cell adhesion was examined with a microscope (Nikon, Japan), and cell viability was assessed using the Cell Counting Kit‐8 (CCK‐8; Dong‐in LS, Republic of Korea). Following incubation for three and five days, the samples were collected, washed with DPBS, and transferred to a new cell culture plate with fresh medium containing 10% CCK‐8 reagent. After 2 h, the solutions were transferred to a 96‐well plate and analyzed at a wavelength of 450 nm using a microplate reader (Bio‐chrom, USA).

### 4.9 In Vivo Evaluation of a Porcine Peripheral Restenosis Model

Stent was placed under general anesthesia using a mixture of zolazepam (50 mg kg^−1^), tiletamine (50 mg kg^−1^), and xylazine (10 mg kg^−1^). Anesthesia was maintained by inhalation of 0.5%–2% isoflurane with oxygen (1:1, 510 mL kg^−1 ^min^−1^). The peripheral artery approach for stent placement was described previously in detail [143, 144]. Briefly, an 8‐Fr sheath was inserted into the right common carotid artery under ultrasonographic guidance (iU22; PHILIPS, Amsterdam, Netherlands), and intra‐arterial heparin was administered at a dose of 100 U kg^−1^. A 6‐Fr catheter with a 0.032 in guidewire was advanced to the superficial femoral artery under fluoroscopic guidance (OEC Elite CFD; GE HealthCare, IL, USA). Pre‐procedural angiography was performed to evaluate anatomical variations and measure the arterial diameter of the targeted popliteal artery. The balloon catheter (diameter, 3 mm; length, 12 mm) was loaded with a stent (diameter, 3 mm; length, 10 mm). It was then guided over a 0.014 in micro‐guidewire and positioned in the popliteal artery. The balloon catheter was fully inflated to 8–10 atm to achieve a stent‐to‐artery diameter ratio of 1.2 under continuous fluoroscopic guidance for 30 s. Post‐procedural angiography was performed immediately after stent placement to confirm the stent location and identify any procedural‐related complications (Figure S25). Post‐balloon dilation was performed using a non‐compliant balloon catheter (diameter, 3.5 mm) to fully expand the deployed stent. After removal of the carotid sheath, a vascular closure device was used to seal the puncture site, followed by manual compression for 10 min. Analgesics (ketorolac trometamol, 30 mg mL^−1^) and antibiotics (gentamicin, 40 mg mL^−1^) were administered for three days. In addition, all animals received daily aspirin (300 mg per day) and clopidogrel (75 mg per day) as dual antiplatelet therapy, starting three days prior to stent placement and continuing until the sacrifice date.

Follow‐up angiography and optical coherence tomography (OCT; Aurios, Dotter, Republic of Korea) were performed immediately and at four weeks after stent placement to assess stent patency, position, and any stent‐related complications. Luminal diameters of the stented popliteal artery were measured using RadiAnt DICOM Viewer (ver. 2020.2; Medixant, Poland).

OCT imaging was performed using an OCT imaging catheter (Benetis; Dotter, Republic of Korea). The catheter was advanced beyond the distal portion of the stented popliteal artery over a 0.014 in micro‐guidewire. Automatic pullback and contrast media flushing were conducted at typical flush rates of 4–5.5 mL s^−1^ and 300–400 psi to generate OCT images. Longitudinal stent areas were measured in 300 µm interval slices along the stented segment. Neointimal thickness was determined by averaging the distance from the outer margin of the stent strut to the neointima surface across the acquired OCT images [145]. The percentage stenotic area was calculated using Equation (5):

(5) $$\mathrm{Stenotic}\;\mathrm{area}\;\left(\%\right)=\frac{neointimal\;area}{stent\;area}\;\times \;100$$

Angiographic and OCT findings were assessed by three independent observers blinded to group assignment, and final measurements were determined by consensus [145].

Both stented popliteal arteries were surgically excised, and tissue samples were preserved in 4% formalin for 72 h. The central segment of each stented artery was cut transversely for histological examination. Specimens were embedded in paraffin blocks and stained with H&E and VVG. Histological analysis with H&E assessed the extent of inflammatory cell infiltration. The extent of inflammatory cell infiltration was evaluated subjectively using the following scale: 1, mild; 2, mild to moderate; 3, moderate; 4, moderate to severe; and 5, severe [144, 145]. Changes in elastic lamina were evaluated using VVG staining and classified based on the vascular layer affected by each stent strut as follows: 0, no injury; 1, disruption of the internal elastic lamina; 2, perforation extending into the media; and 3, penetration through the external elastic lamina into the adventitia [146, 147]. All specimens were scanned using a digital slide scanner (Pannoramic 250 FLASH III, 3D HISTECH, Hungary), and measurements were performed using a digital microscope viewer (CaseViewer, 3D HISTECH, Hungary).

Immunohistochemistry (IHC) analysis was performed on paraffin‐embedded slices using the TUNEL assay. The extent of TUNEL‐positive cell coverage was evaluated subjectively using the following scale: 1, mild; 2, mild to moderate; 3, moderate; 4, moderate to severe; and 5, severe [63, 148]. Immunofluorescence (IF) analysis was performed using primary antibodies against CD31 as an endothelial cell marker, VCAM‐1 as a marker of activated macrophages, and α‐SMA as a marker of VSMC migration. Secondary antibody labeling was conducted using Alexa Fluor 488 or 594 conjugates, and nuclei were counterstained with DAPI. The extent of CD31‐, VCAM‐1‐, and α‐SMA‐positive cell coverage was graded subjectively based on density and distribution around the stent struts using the following scale: 1, mild; 2, mild to moderate; 3, moderate; 4, moderate to severe; and 5, severe. IHC and IF images were evaluated using CaseViewer software, with five randomly selected fields per sample.

### 4.10 Statistical Analysis

All quantitative experimental data are presented as the mean ± standard deviation and were obtained from a minimum of three replicates per group. Data were analyzed using IBM SPSS Statistics 26 (IBM, Armonk, USA). Statistical significance was assessed using one‐way analysis of variance (ANOVA) with Tukey's post hoc test, the Kruskal–Wallis H test, and the Mann–Whitney *U*‐test with pairwise comparisons. Differences were considered statistically significant at *p* < 0.05, and significance levels are denoted as follows: *p < 0.05, **p < 0.01, ***p < 0.005, and ****p < 0.001. Comprehensive statistical analyses are detailed in Tables S2–S15 in the Supporting Information.

## Author Contributions

J.S., D.W., and H.L. wrote and edited the manuscript throughout all of the activities. J.P. and H.J. edited the manuscript. S.K. and J.P. supported the fabrication of the 3D‐printed stent. S.B., H.K., J.G., and C.M. performed the structural and mechanical analyses. T.J. contributed to the understanding of S‐PIII and Janus nanoengineering. M.K. conducted cryo‐TEM analyses. D.L., D.L., K.Y., G.C., and S.L. contributed to the explanation of in vitro tests. J.K. and Y.P. contributed to the understanding of animal results. J.S., D.W., H.L., J.P., and H.J. contributed to the interpretation and discussion of the results.

## Funding

This work was supported by the National Research Foundation of Korea (NRF) grant funded by the Korea government (MSIT) (Nos. RS‐2024‐00405381; RS‐2025‐00513935; RS‐2026‐25469139; RS‐2026‐25475904); the Korea Institute of Marine Science & Technology Promotion (KIMST) funded by the Ministry of Oceans and Fisheries (RS‐2024‐00405273); the Korean Fund for Regenerative Medicine (KFRM) grant funded by the Korea government (the Ministry of Science and ICT and the Ministry of Health & Welfare, KFRM 24A0105L1); the Nano Material Technology Development Program through the NRF grant funded by the Korea government (MSIT) (No. RS‐2022‐NR068191); Korea Institute of Industrial Technology (KITECH) (KITECH UR‐26‐0018).

## Ethics Statement

The animal study was approved by the Institutional Animal Care and Use Committee (IACUC) at the Institute for Life Sciences, in compliance with the US National Institutes of Health guidelines for the ethical treatment of laboratory animals (IACUC No. 2023‐30‐228). A total of 24 popliteal arteries from 12 Yorkshire pigs (weight: 45.2–49.4 kg; International Animal Experiment Center, Pocheon, Korea) were randomly assigned to three groups, with eight popliteal arteries from four pigs in each group.

## Conflicts of Interest

The authors declare no conflict of interest.

## Supporting information




**Supporting File 1**: advs75026‐sup‐0002‐SuppMat.docx.

**Supporting File 2**: advs75026‐sup‐0001‐Movie1.mp4.

## Data Availability

The data that support the findings of this study are available from the corresponding author upon reasonable request.;
