# Supplementary material for: Mechanically Robust Biodegradable Stents With Theragenerative Vascular Responses via Combined 3D Printing and Janus Nanoengineering
Source: Adv Sci (Weinh). 2026 Mar 31;13(34):e23965. doi: 10.1002/advs.202523965 (PMC13285157; doi:10.1002/advs.202523965)
Supplement: Supplementary file 1 — Supporting File 1: advs75026‐sup‐0002‐SuppMat.docx. [file ADVS-13-e23965-s002.docx]

Supporting Information

**Mechanically Robust Biodegradable Stents with Theragenerative Vascular Responses via Combined 3D Printing and Janus Nanoengineering**

*Jong Hwa Seo****^†^****, Dong-Sung Won****^†^****, Hyun Lee****^†^****, Seojoon Bang, Hyeong Seok Kang, Ju Yeong Gwon, Chan Ho Moon,* *Ji Won Kim^3^, Yubeen Park, Minho Kang, Dong Yun Lee, Donghyun Lim, Kisuk Yang, Gi Doo Cha, Soo-Hong Lee, Tae-Sik Jang, Seokbeom Kim, Jun-Kyu Park, Jung-Hoon Park^*^, Hyun-Do Jung^*^.*

J. H. Seo, S. Bang, H. S. Kang, C. H. Moon, H.-D. Jung

Division of Materials Science and Engineering, Hanyang University, Seoul 04763, Republic of Korea

E-mail: hdjung@hanyang.ac.kr, Hyun-Do Jung

D.-S. Won, J. W. Kim, Y. Park, J.-H. Park

Department of Convergence Medicine, Asan Medical Center, University of Ulsan College of Medicine, 88 Olympic-ro 43-gil, Songpa-gu, Seoul 05505, Republic of Korea

Biomedical Engineering Research Center, Asan Institute for Life Sciences, Asan Medical Center, 88 Olympic-ro 43-gil, Songpa-gu, Seoul 05505, Republic of Korea

E-mail: jhparkz@amc.seoul.kr, J.-H. Park

H. Lee

Research Institute of Intelligent Manufacturing & Materials Technology, Korea Institute of Industrial Technology, 156, Gaetbeol-ro, Yeonsu-gu, Incheon 21999, Republic of Korea

J. Y. Gwon, D. Y. Lee, D. Lim

Department of Bioengineering, Hanyang University, Seoul 04763, Republic of Korea

M. Kang

Department of Biotechnology, The Catholic University of Korea, Bucheon 14662, Republic of Korea

Department of Biomedical-Chemical Engineering, The Catholic University of Korea, Bucheon 14662, Gyeonggi-do, Republic of Korea

K. Yang

Division of Bioengineering, College of Life Sciences and Bioengineering, Incheon National University, Incheon 22012, Republic of Korea

G. D. Cha

Department of Systems Biotechnology, Chung-Ang University, Anseong-si, Gyeonggi-do, 17546, Republic of Korea

S.-H. Lee

Department of Biomedical Engineering, Dongguk University, Seoul 04620, Republic of Korea

T.-S. Jang

School of Biomedical Convergence Engineering, Pusan National University, Yangsan 50612 Republic of Korea

S. Kim

Plancklab Inc., Seoul 08584, Republic of Korea

J-.K. Park

IMT Inc., Gwangju, 61086 Republic of Korea

†These authors contributed equally to this work.

*These authors are co-corresponding authors.


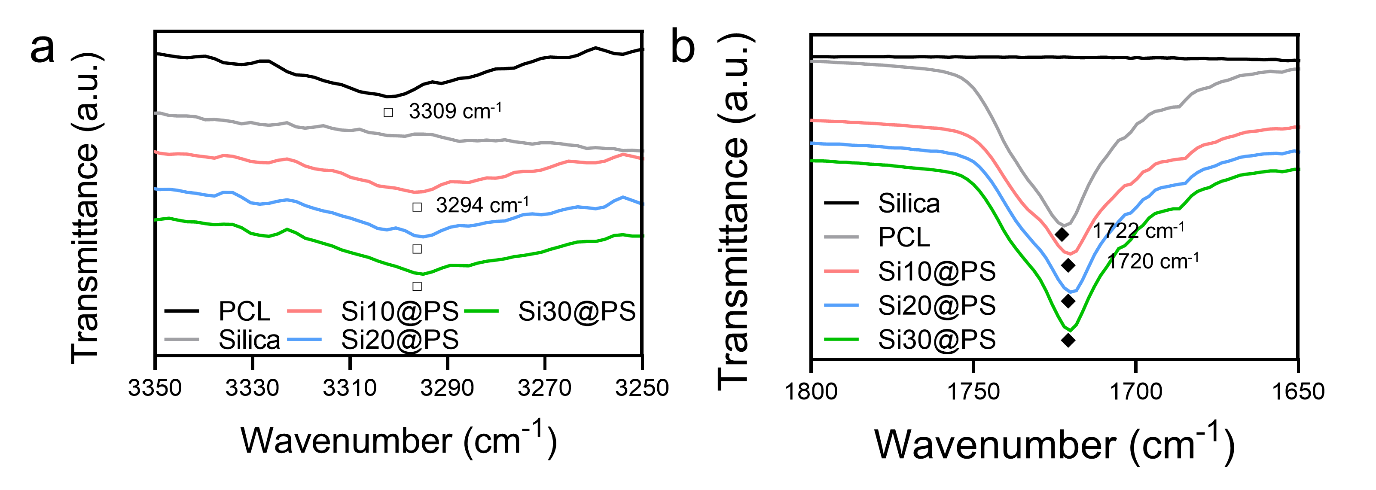


**Figure S1.** High resolution FT-IR spectra of sol–gel-derived silica, PS, and SiPS chips with varying silica contents. (a) O–H (□) and (b) C=O (◆) bond regions highlighting characteristic peak shifts that confirm successful synthesis and silica incorporation into the PS matrix (n = 3).

**Figure S2.** High-resolution TGA curves for 3D-printed SiPS with varying silica concentrations.


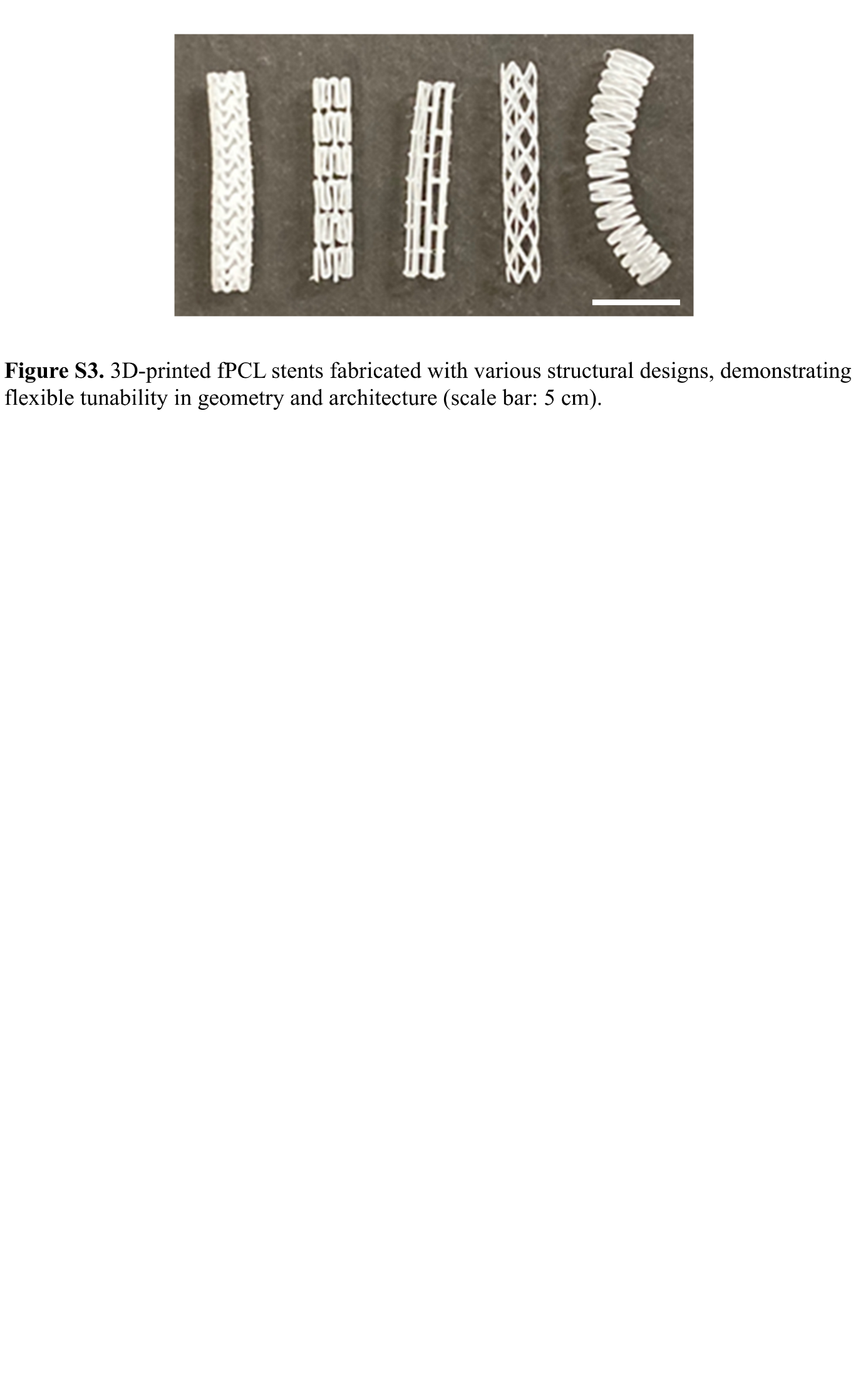


**Figure S3.** 3D-printed SiPSs with various structural designs, demonstrating flexible tunability in geometry and architecture (scale bar: 5 cm).

**Figure S4.** EDS chemical composition analysis of PS and SiPSs with various silica contents.


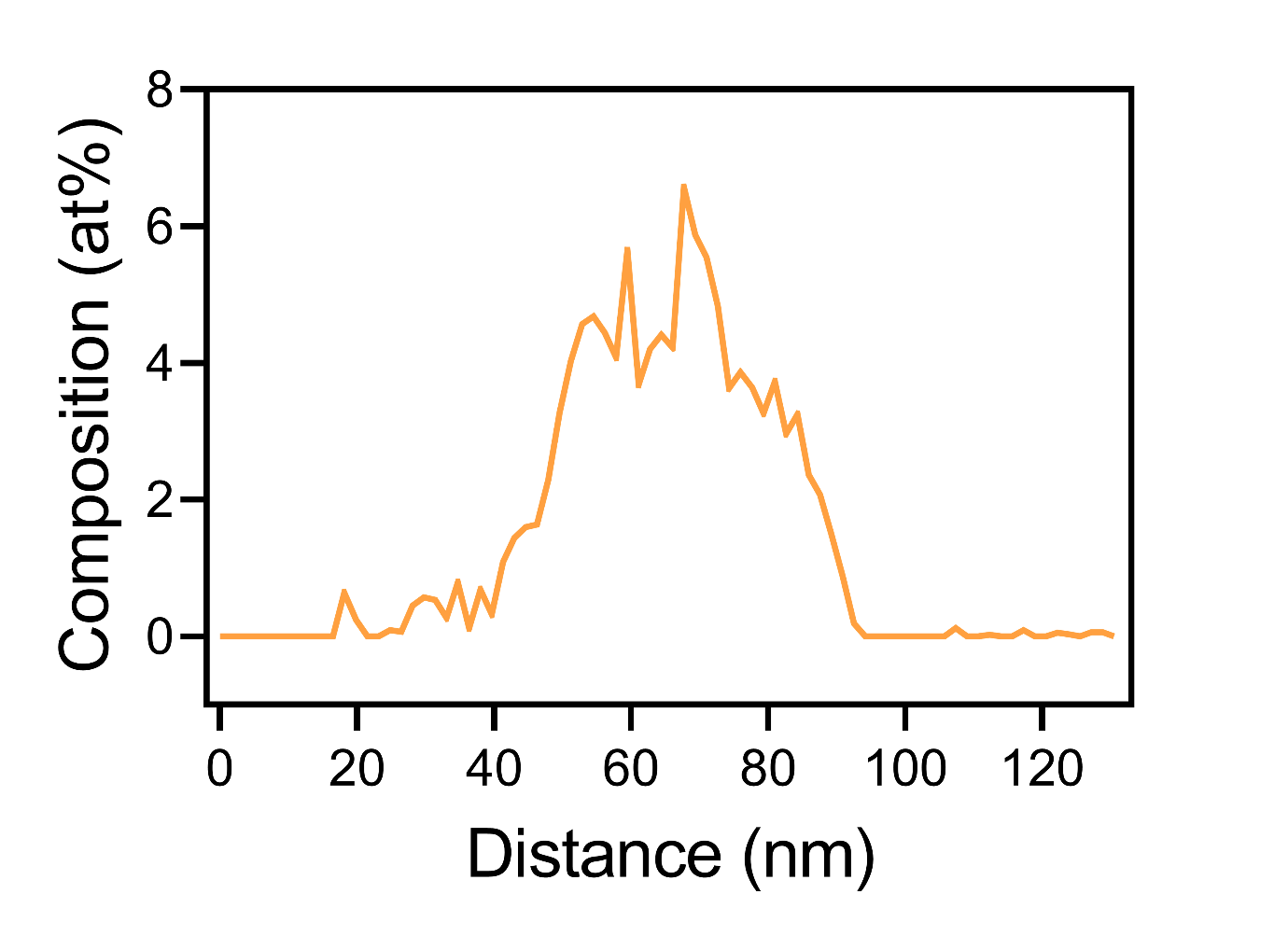


**Figure S5.** High-resolution EDS spectra of elemental Ta in 3D-printed SiPS after Ta S-PIII treatment.


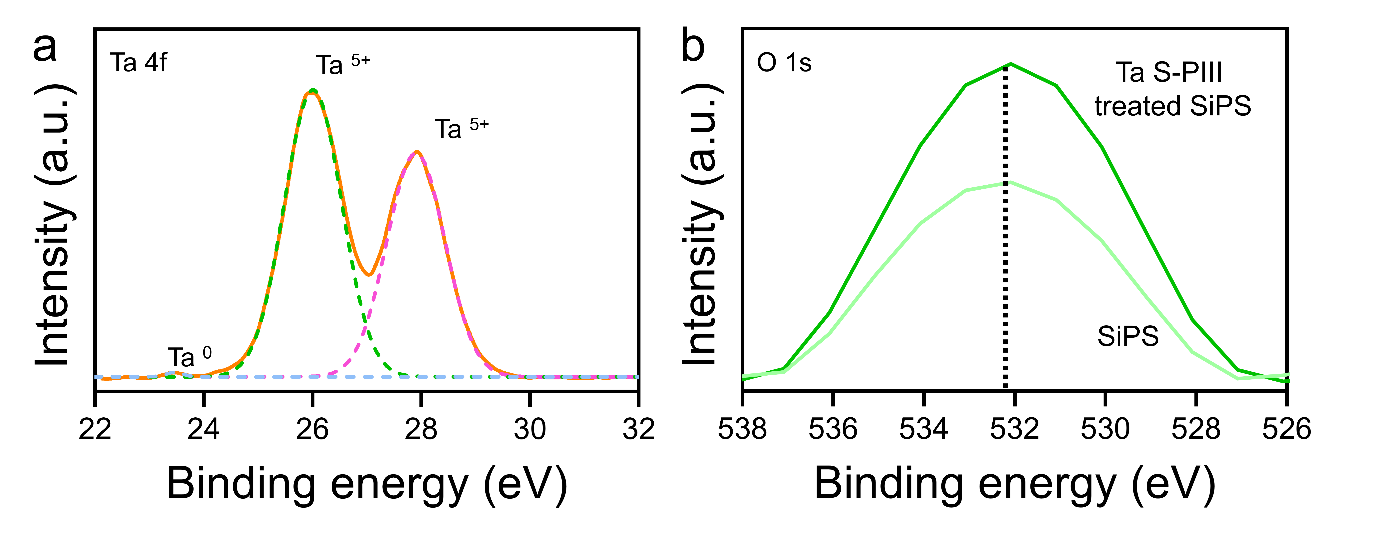


**Figure S6.** High-resolution XPS spectra of (a) Ta 4f on the surface of 3D-printed SiPS after Ta S-PIII treatment and (b) O 1s on the surface of 3D-printed SiPS before and after Ta S-PIII treatment.


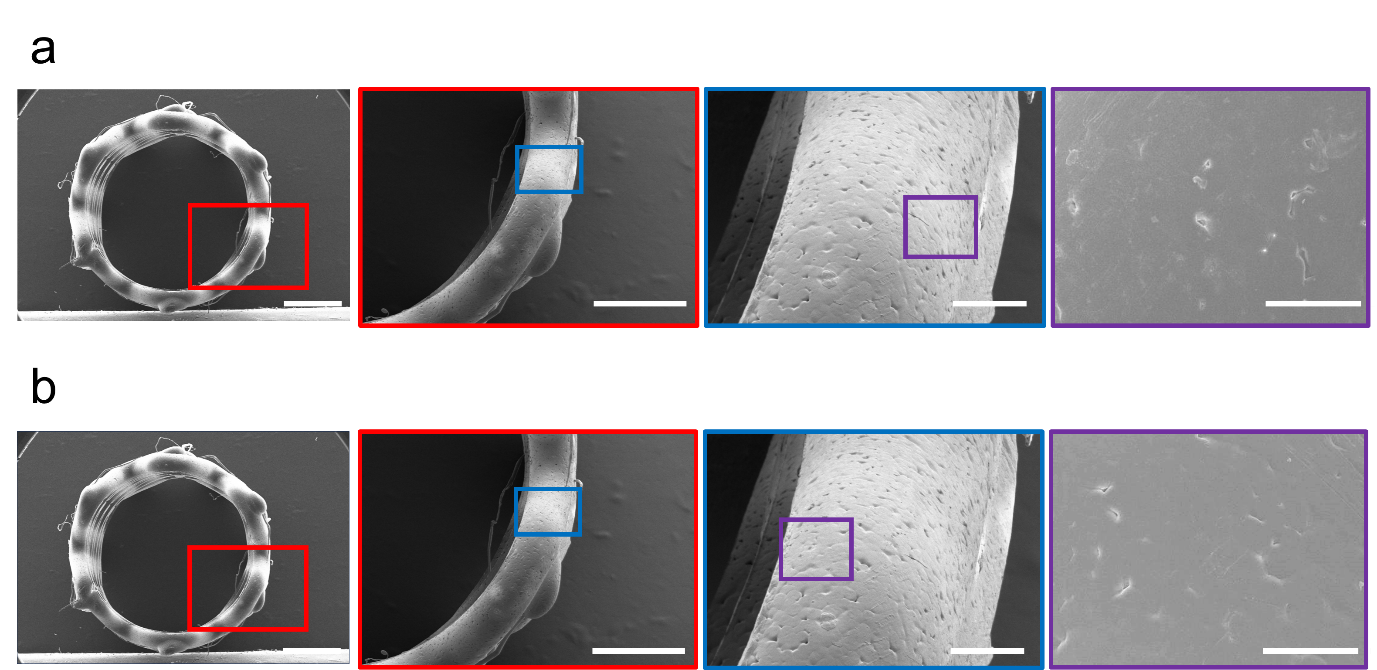


**Figure S7.** Sequential SEM images showing stepwise magnification of (a) the abluminal surface (scale bar: 1 mm) and (b) the luminal surface (scale bar: 1 mm), where the red (scale bar: 500 μm), blue (scale bar: 100 μm), and purple boxed regions (scale bar: 20 μm) indicate the areas selected for progressive enlargement from low to high magnification.


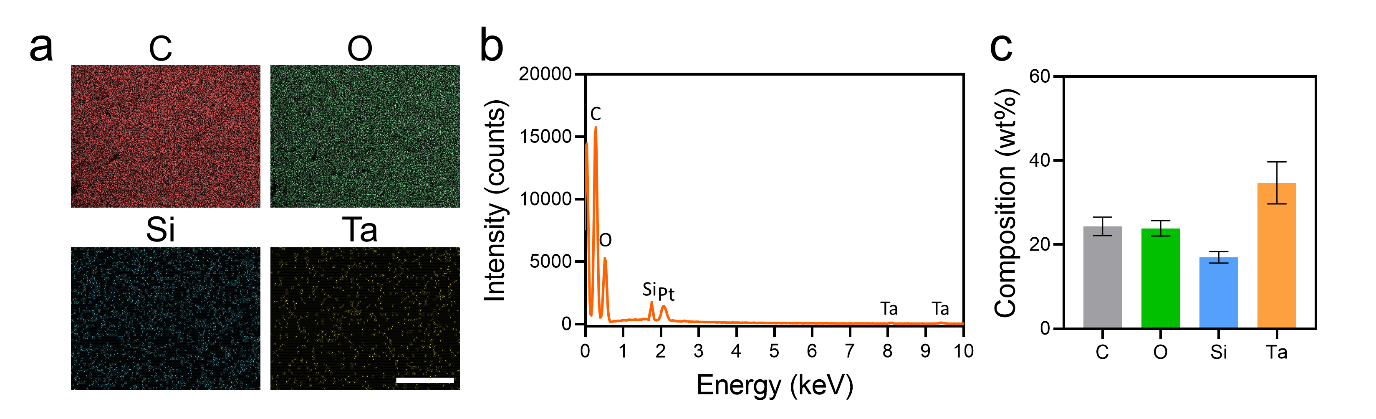


**Figure S8.** (a) EDX elemental mapping images (scale bar: 20 μm), (b) corresponding EDX spectrum, and (c) quantitative elemental composition of the abluminal surface.


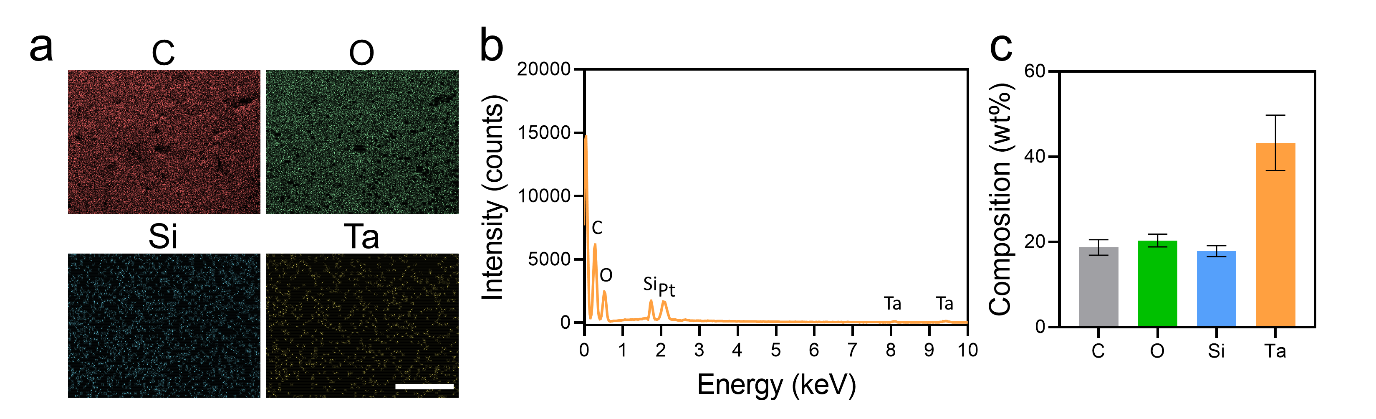


**Figure S9.** (a) EDX elemental mapping images (scale bar: 20 μm), (b) corresponding EDX spectrum, and (c) quantitative elemental composition of the luminal surface.


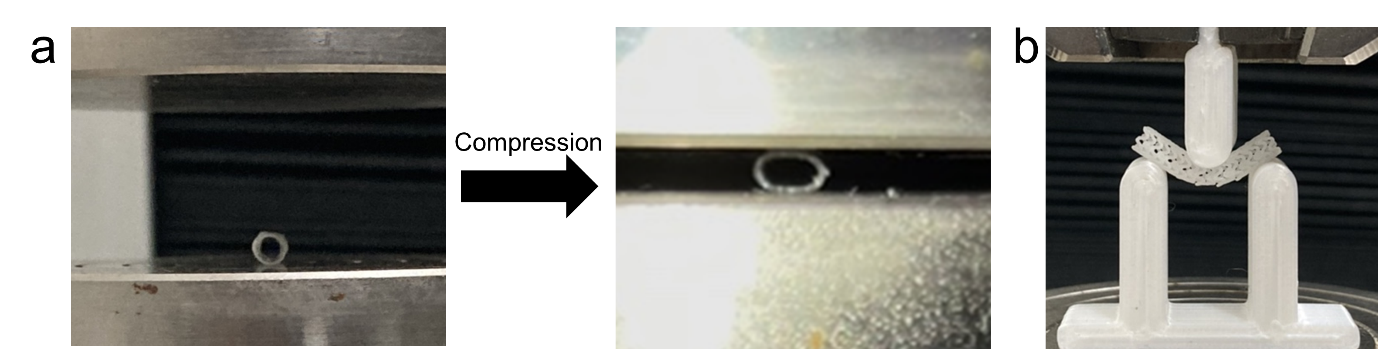


**Figure S10.** Representative experimental images illustrating the (a) radial force and (b) flexibility of 3D-printed SiPSs.


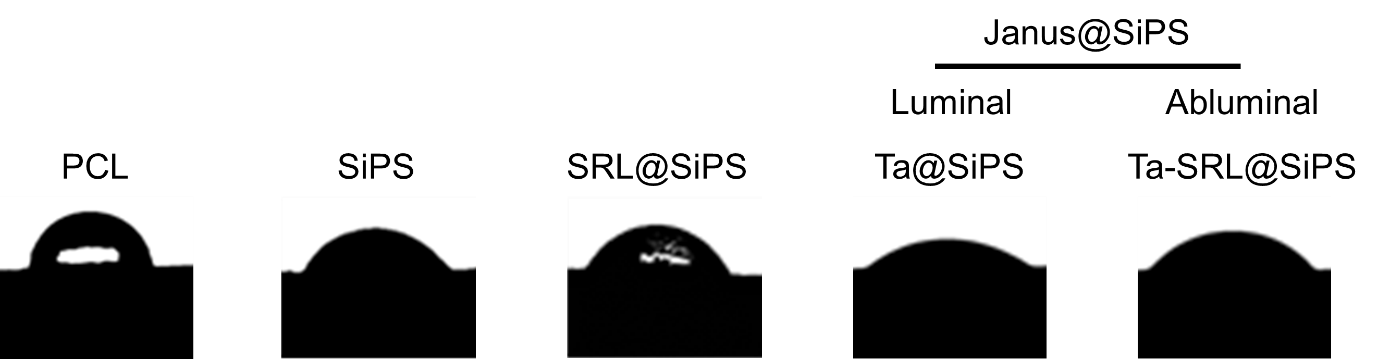


**Figure S11.** Optical images of water contact angles on 3D-printed SiPS surfaces indicating surface wettability.


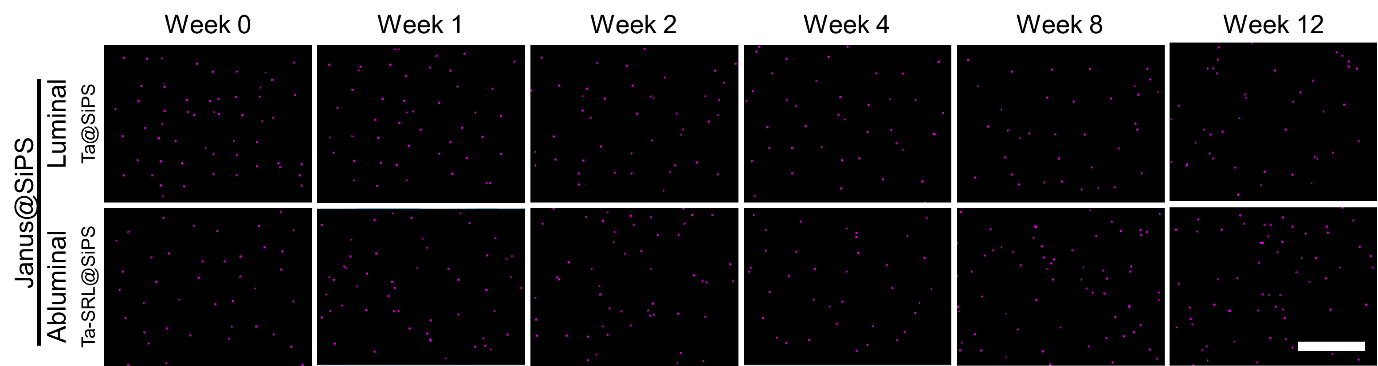


**Figure S12.** Time-dependent Ta elemental mapping of the luminal and abluminal surfaces of Janus@SiPSs (scale bar: 5 nm).


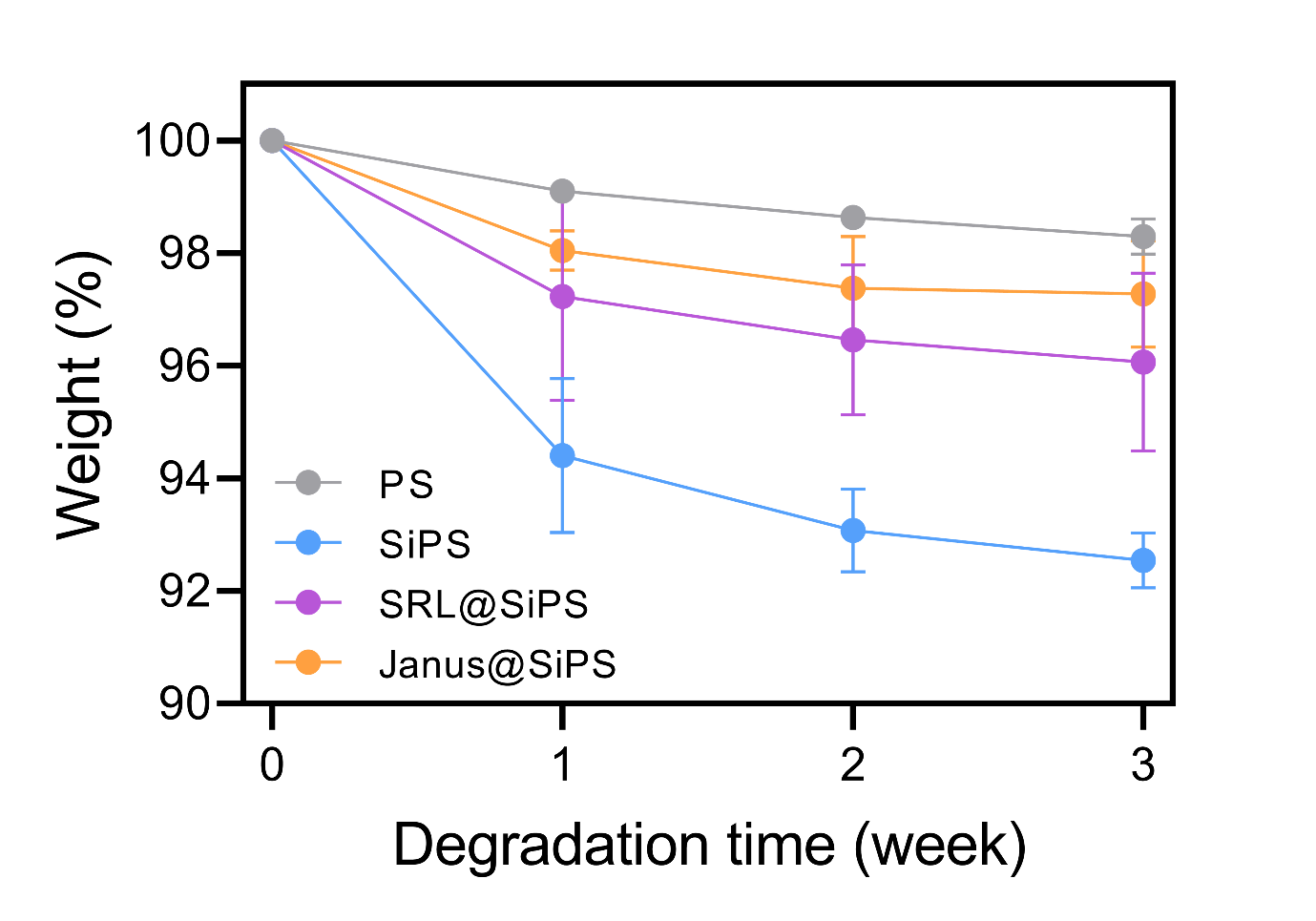


**Figure S13.** Ratio of weight change for 3 weeks for 3D-printed PS, SiPS, SRL@SiPS, and Janus@SiPS through accelerated degradation experiments (n = 3).


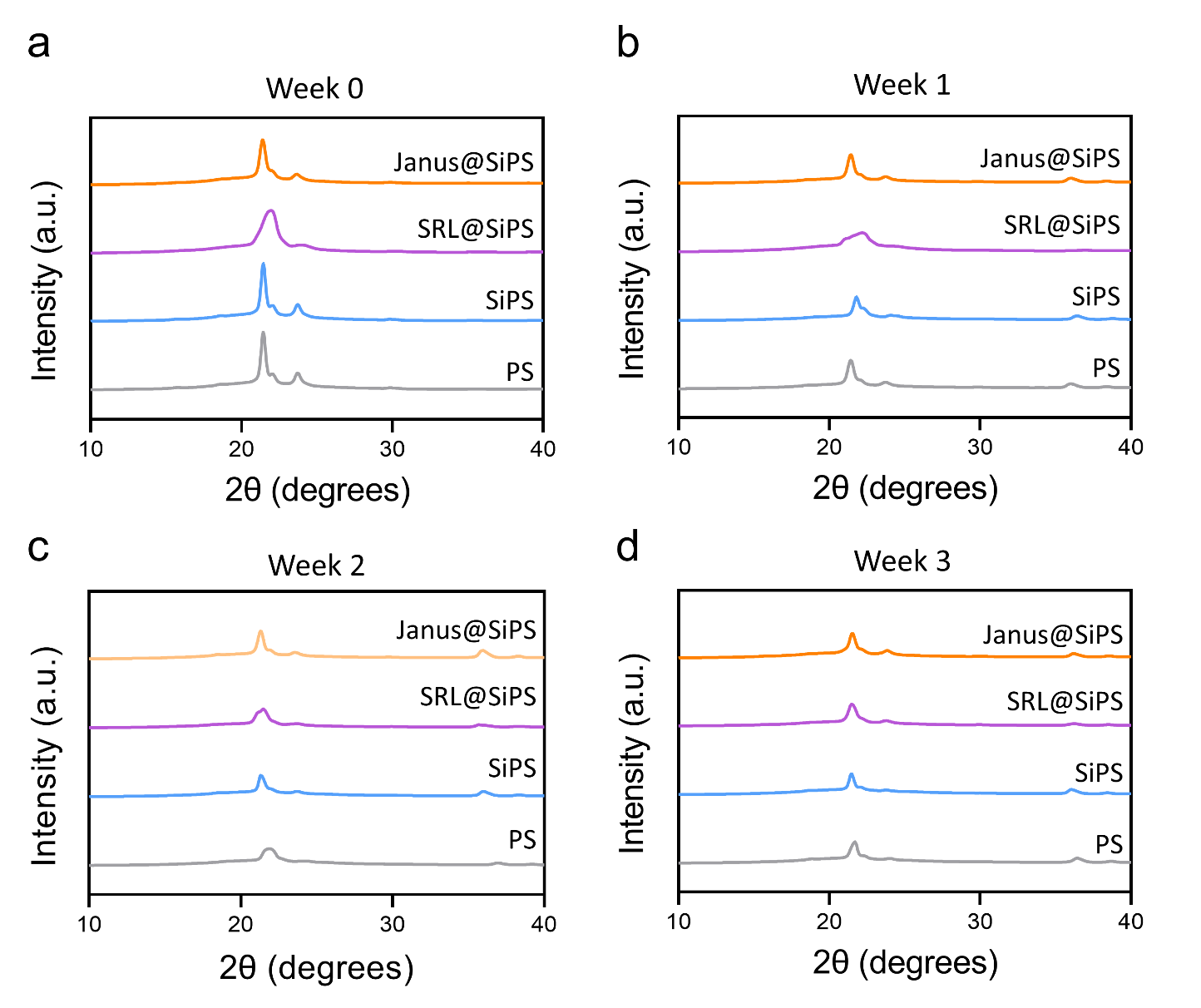


**Figure S14.** XRD patterns of PS, SiPS, SRL@SiPS, and Janus@SiPS obtained during accelerated degradation at (a) 0, (b) 1, (c) 2, and (d) 3 weeks.

**Figure S15.** Quantitative crystallinity of PCL in PS, SiPS, SRL@SiPS, and Janus@SiPS calculated from the XRD patterns obtained during accelerated degradation at 0, 1, 2, and 3 weeks (n = 3). Data are shown as mean ± standard deviation (SD). Normality was tested using the Shapiro-Wilk method, and one-way ANOVA followed by Tukey’s HSD post hoc analysis was applied, with significance at *p<0.05, **p<0.01, ***p<0.005, and ****p<0.001.


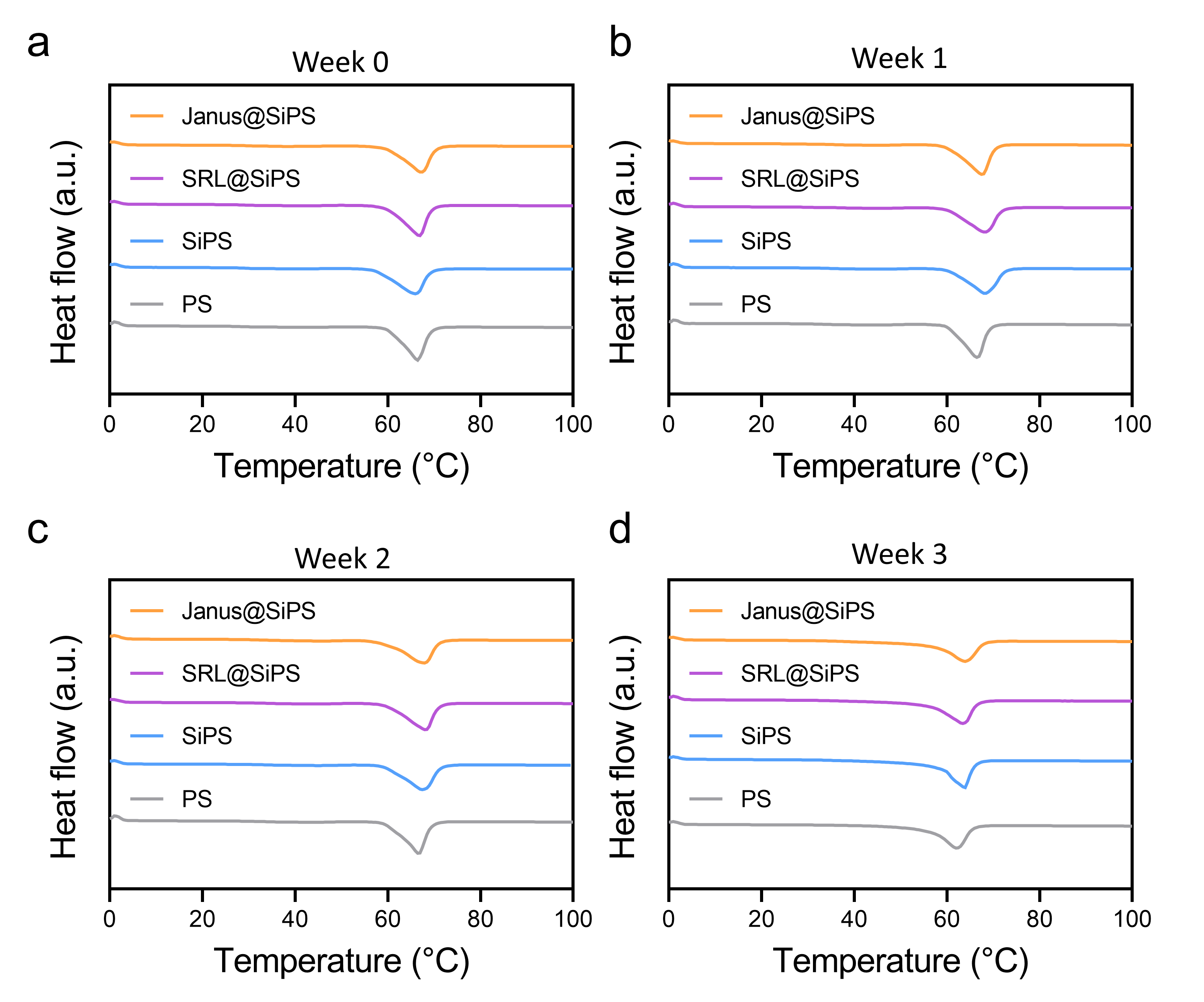


**Figure S16.** DSC analysis of crystallinity evolution during accelerated degradation at (a) 0, (b) 1, (c) 2, and (d) 3 weeks.


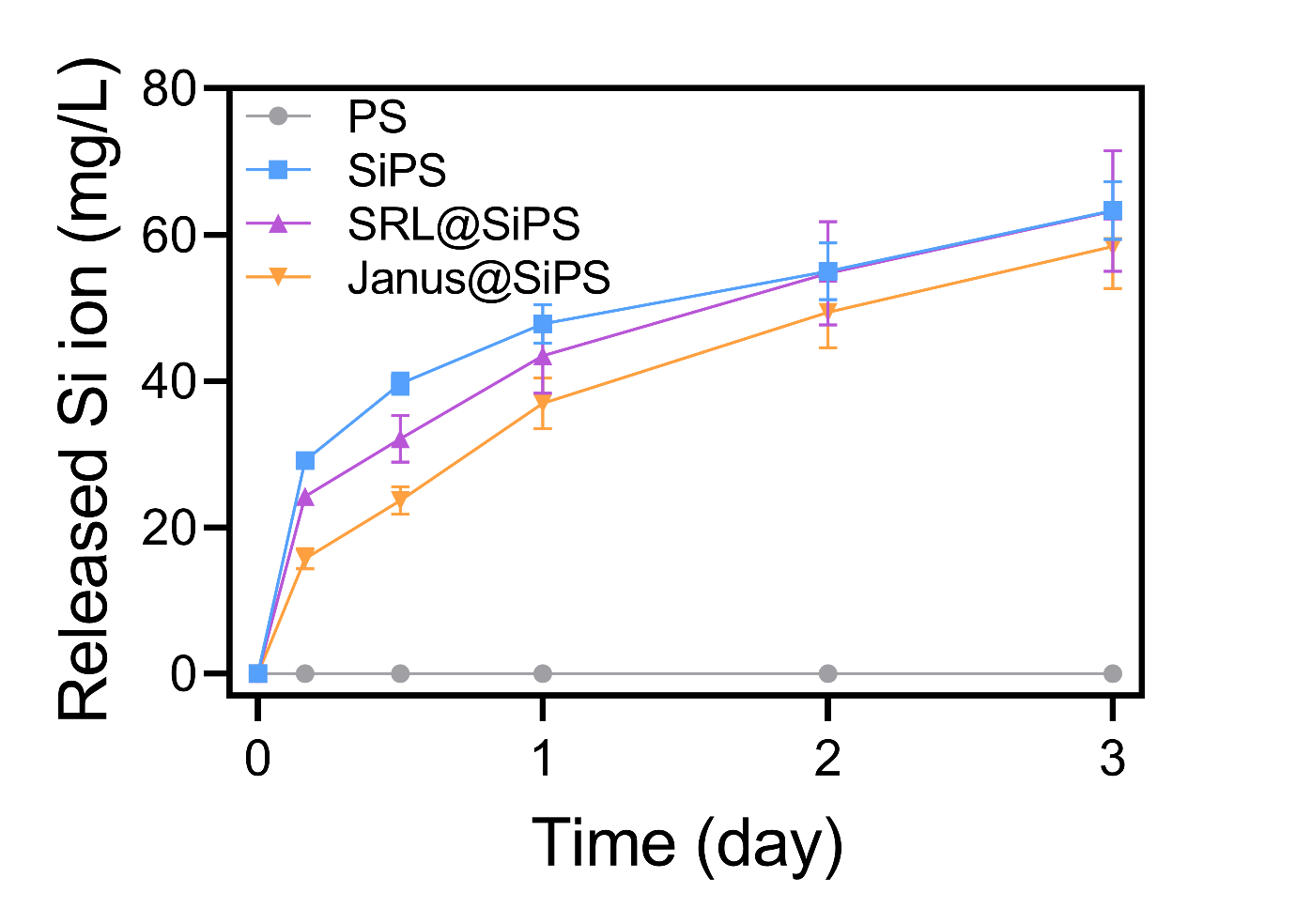


**Figure S17.** Initial burst release behavior of accumulated Si ions from various 3D-printed stents (n = 3).


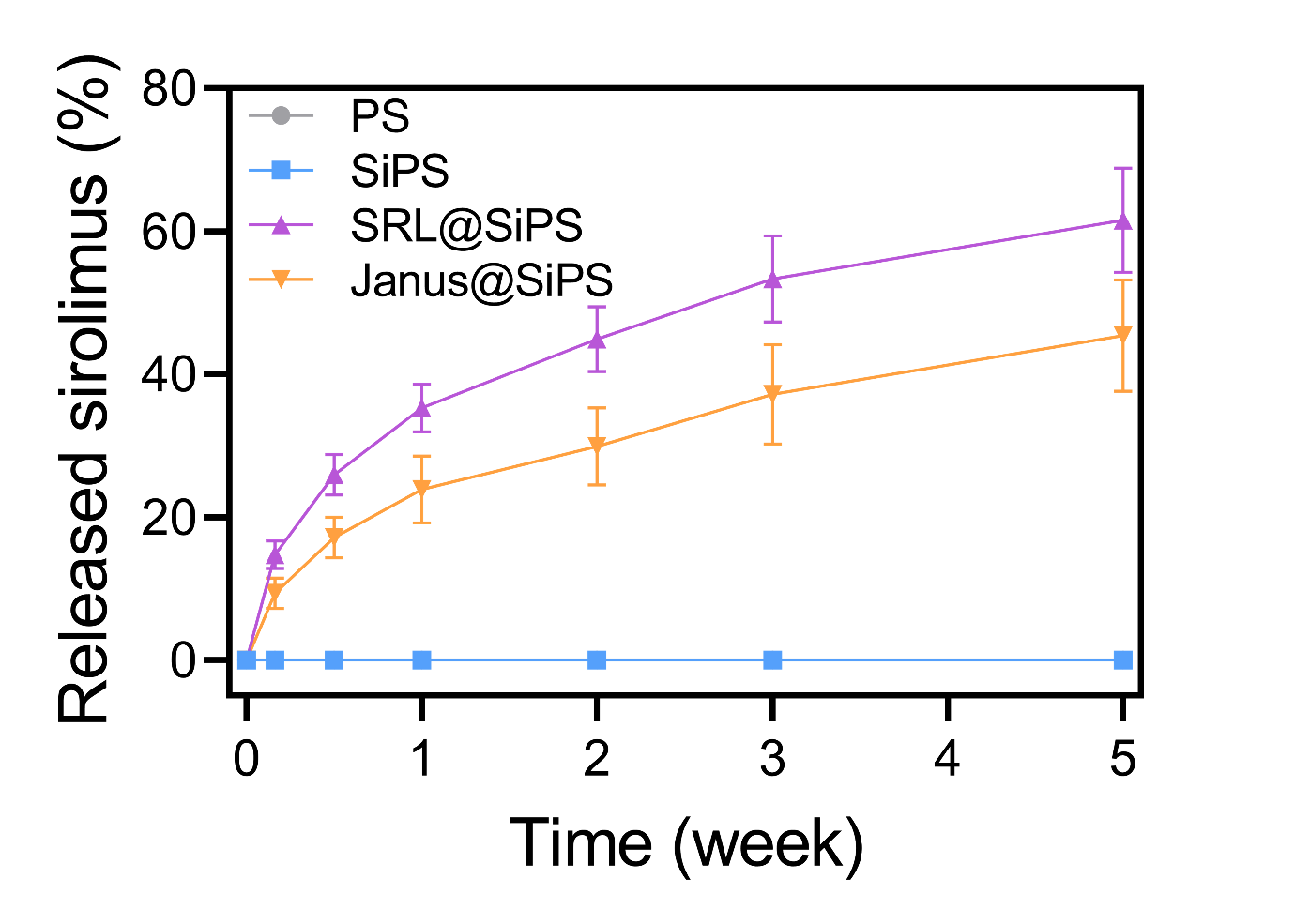


**Figure S18.** Initial burst release behavior of accumulated sirolimus from various 3D-printed stents (n = 3).


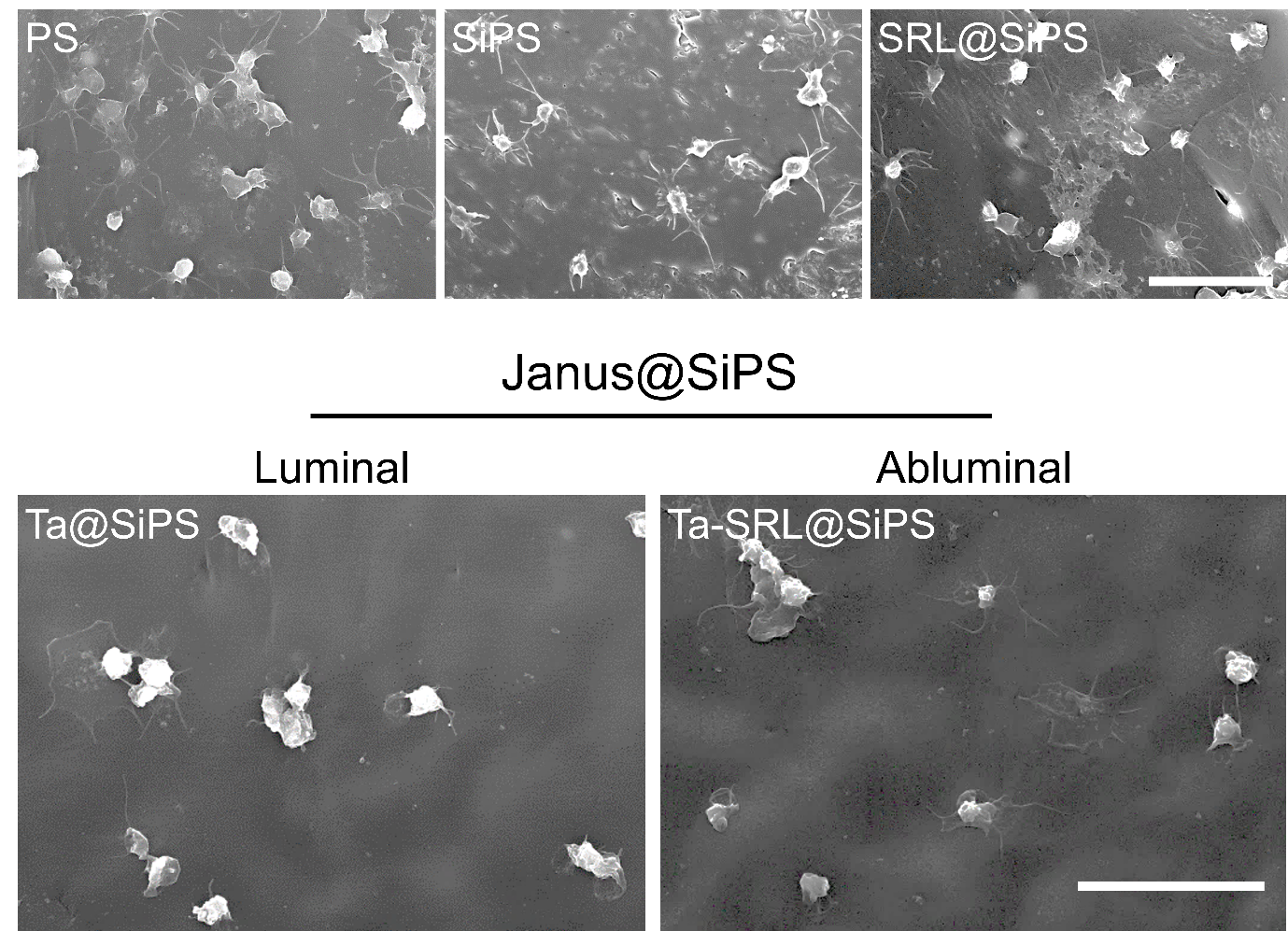


**Figure S19.** Representative FE-SEM images of platelets adhered to different 3D-printed SiPSs (scale bar: 10 μm).


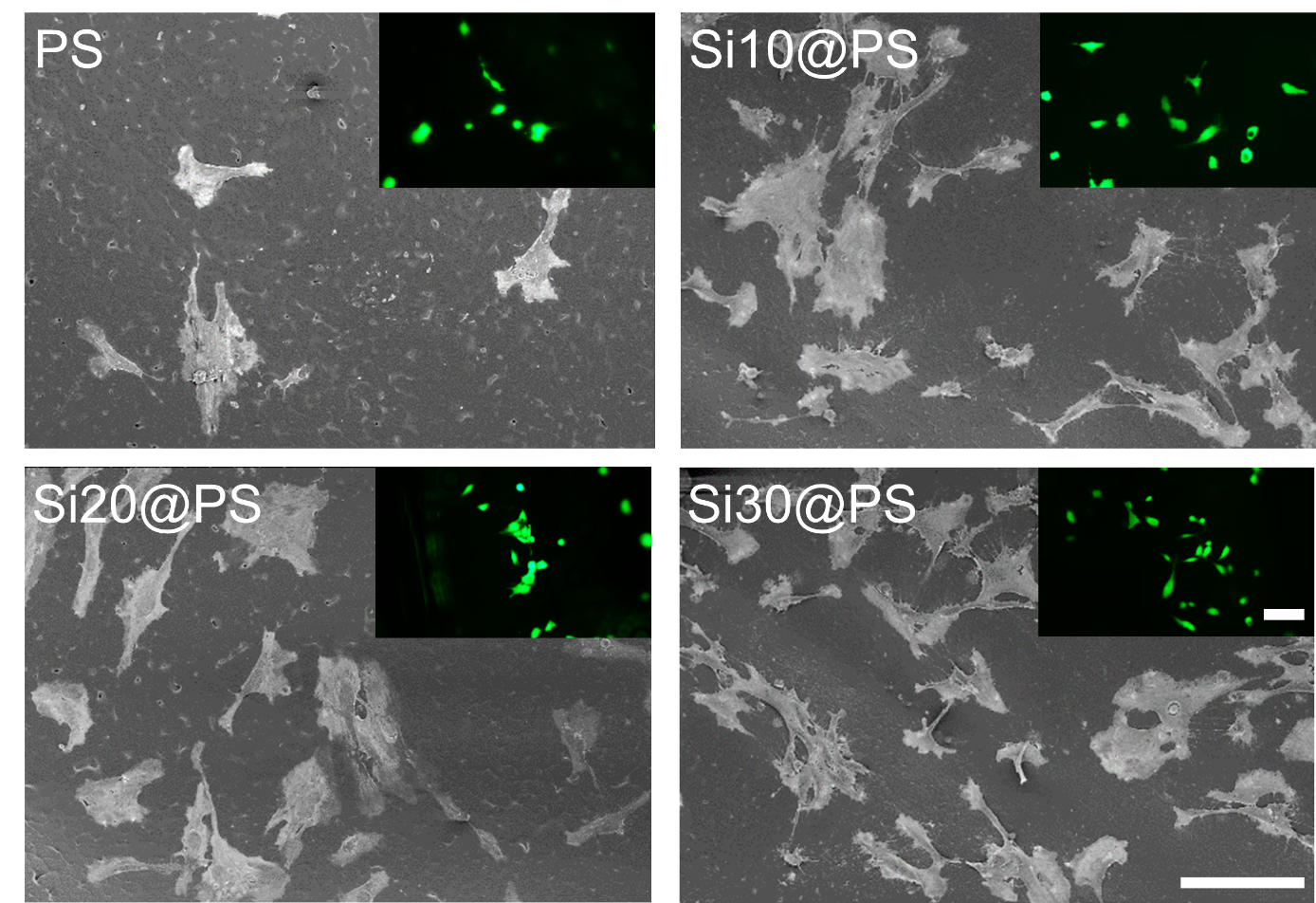


**Figure S20.** FE-SEM and CLSM inlet images of HUVECs cultured for one day on 3D-printed SiPSs with varying silica contents (scale bar: 100 μm).

**Figure S21.** Rate of HUVEC proliferation after 3 and 5 days of culture (n = 3). Data are shown as mean ± standard deviation (SD). Normality was tested using the Shapiro-Wilk method, and one-way ANOVA followed by Tukey’s HSD post hoc analysis was applied, with significance at *p<0.05, **p<0.01, ***p<0.005, and ****p<0.001.


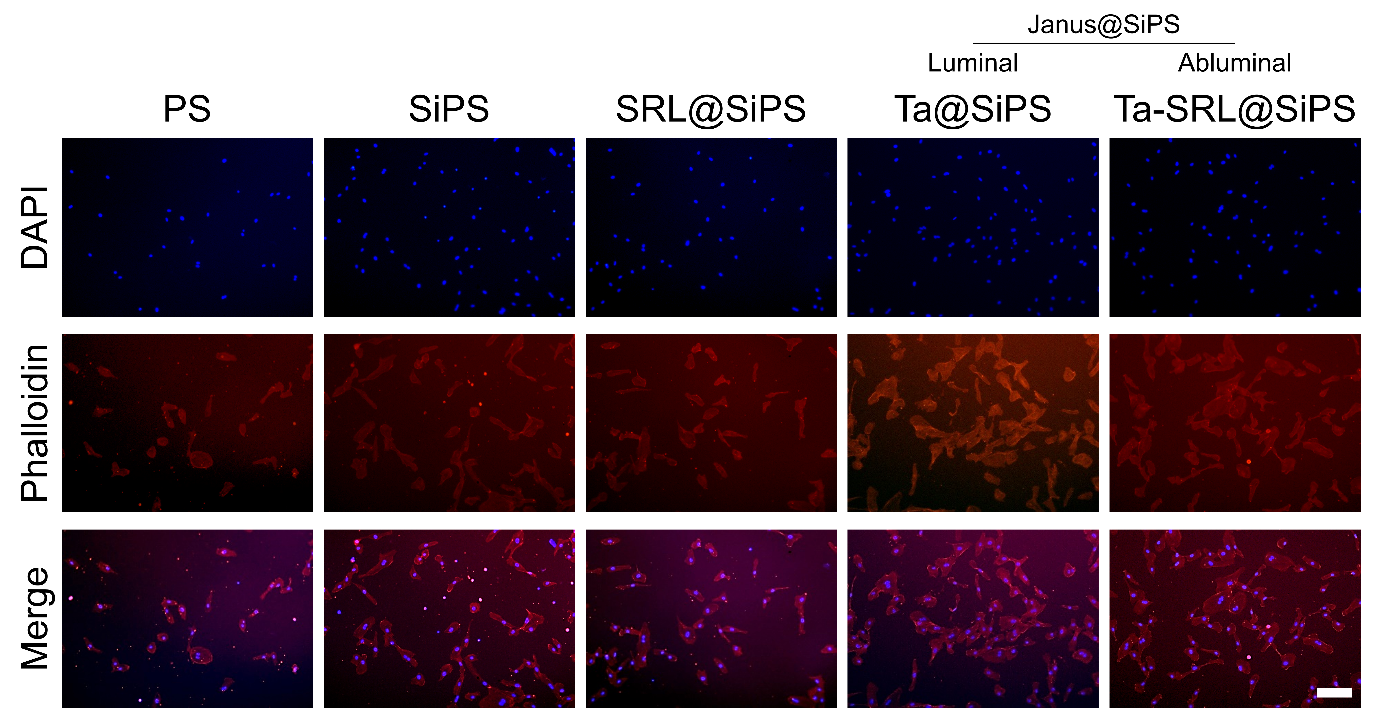


**Figure S22.** CLSM images of HUVEC attachment on different 3D-printed PS surfaces after 1 day of culture (scale bar: 100 μm).


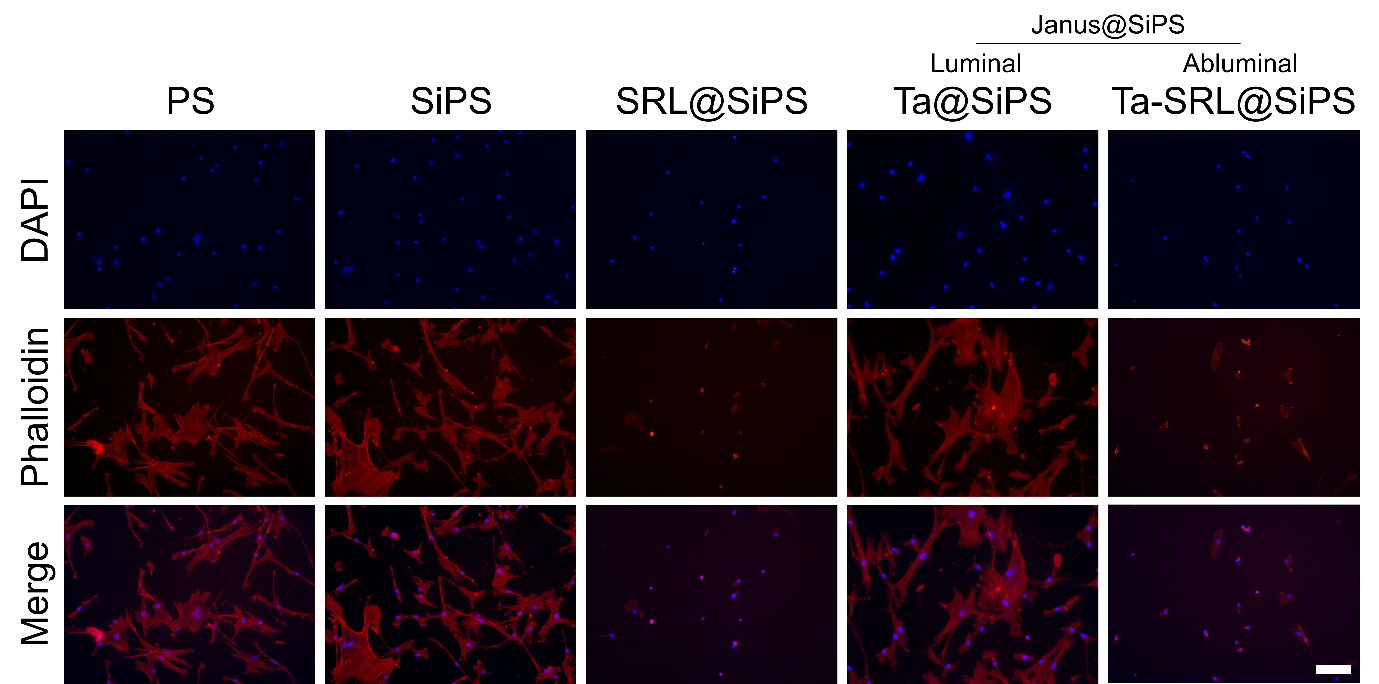


**Figure S23.** CLSM images of SMC attachment on different 3D-printed PS surfaces after 1 day of culture (scale bar: 100 μm).

**Figure S24.** Quantitative analysis of cell coverage on different 3D-printed PS surfaces determined from CLSM images after 1 day of SMC culture (n = 3). Data are shown as mean ± standard deviation (SD). Normality was tested using the Shapiro-Wilk method, and one-way ANOVA followed by Tukey’s HSD post hoc analysis was applied, with significance at *p<0.05, **p<0.01, ***p<0.005, and ****p<0.001.


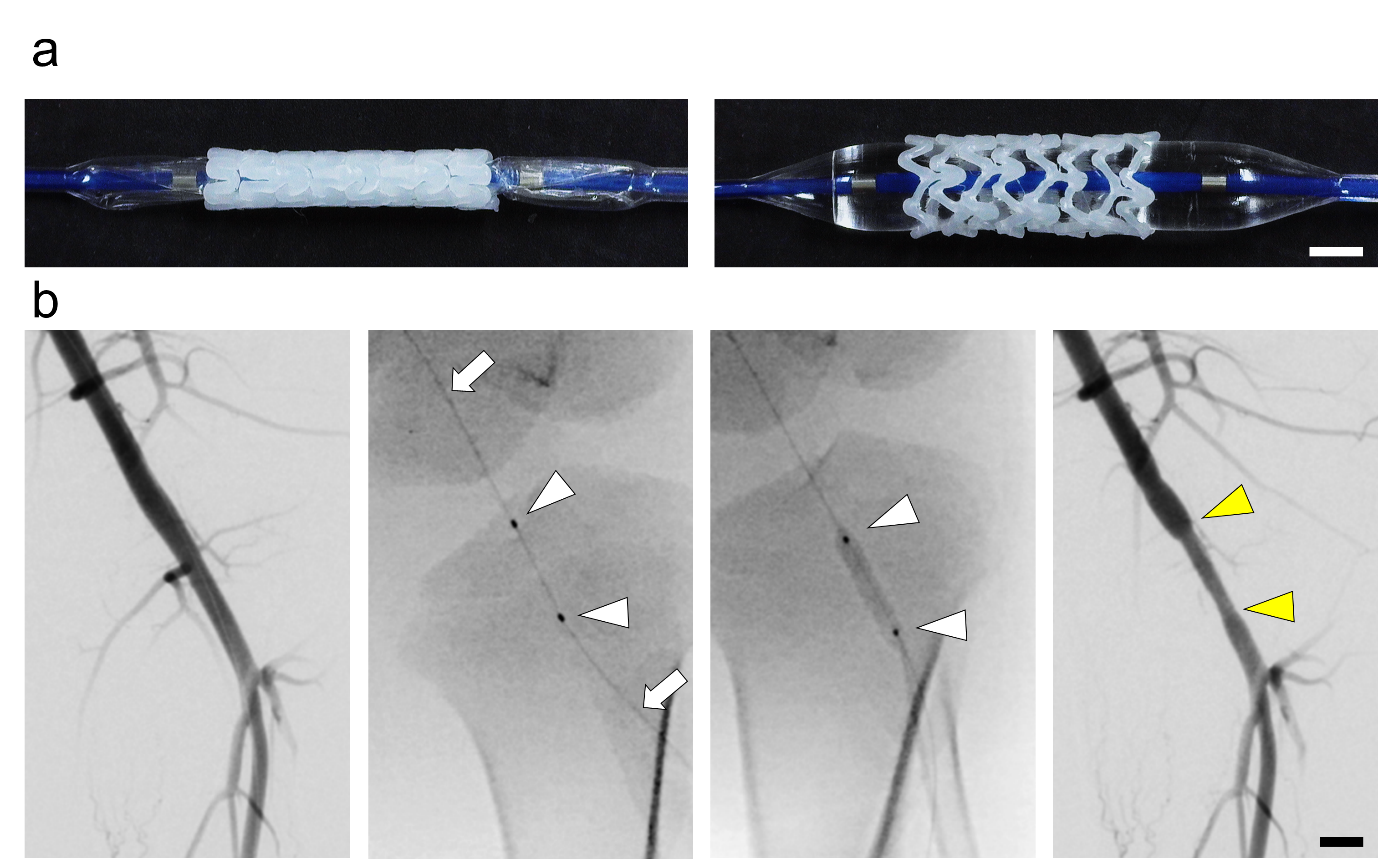


**Figure S25.** Balloon-expandable 3D-printed SiPS and technical steps for stent placement in the porcine popliteal artery. (a) Photograph showing the crimped and expanded 3D-printed SiPS onto the balloon catheter (scale bar: 2 mm). (b) Fluoroscopic images depicting the pre-procedural angiography and the crimped SiPS with balloon catheter (white arrowheads) advanced over a 0.014 in micro-guidewire (white arrows). The balloon is then fully inflated to implant the SiPS into the targeted popliteal artery. Post-procedural angiography showing the luminal patency of the SiPS (yellow arrowheads) in the porcine popliteal artery (scale bar: 6 mm).


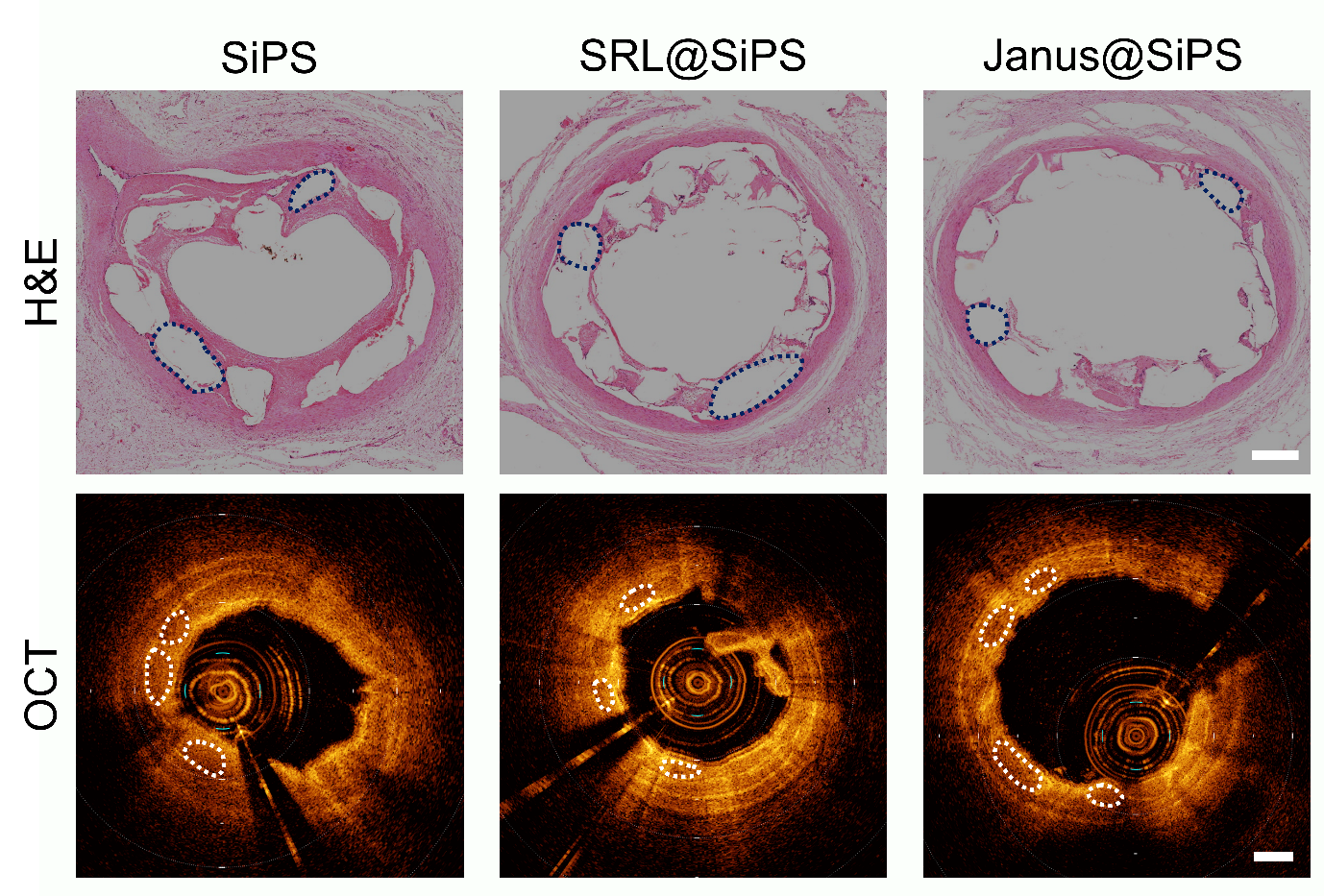


**Figure S26.** Representative H&E-stained microscopic and OCT images showing the entire luminal structure obtained at 4 weeks after stent placement in the study groups (scale bar: 500 μm, blue and white circles mean stent strut, respectively)


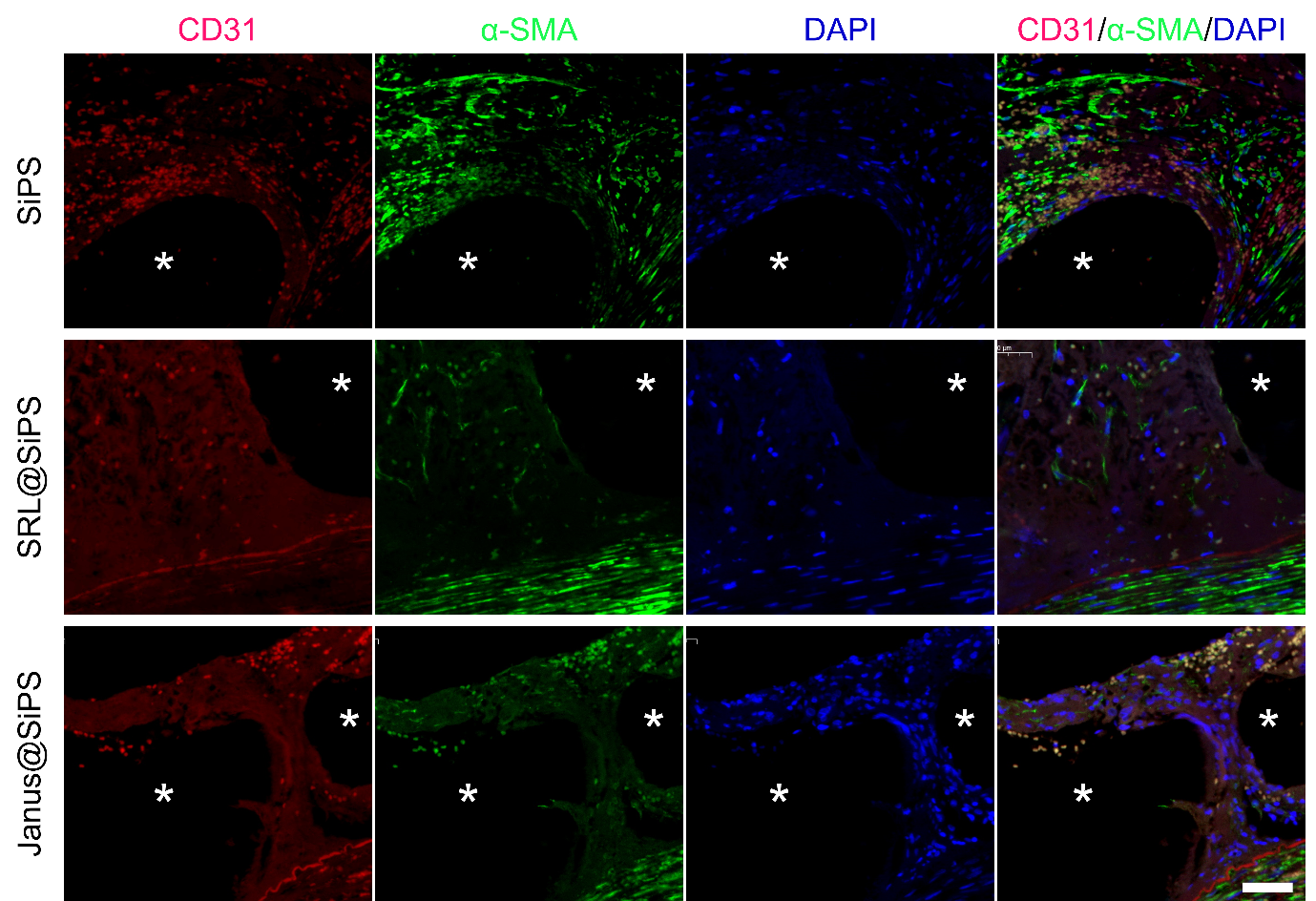


**Figure S27.** Representative CD31 and α-SMA immunofluorescence images around the stent strut (white stars) captured at four weeks after stent placement in the porcine popliteal artery (scale bar: 50 μm).


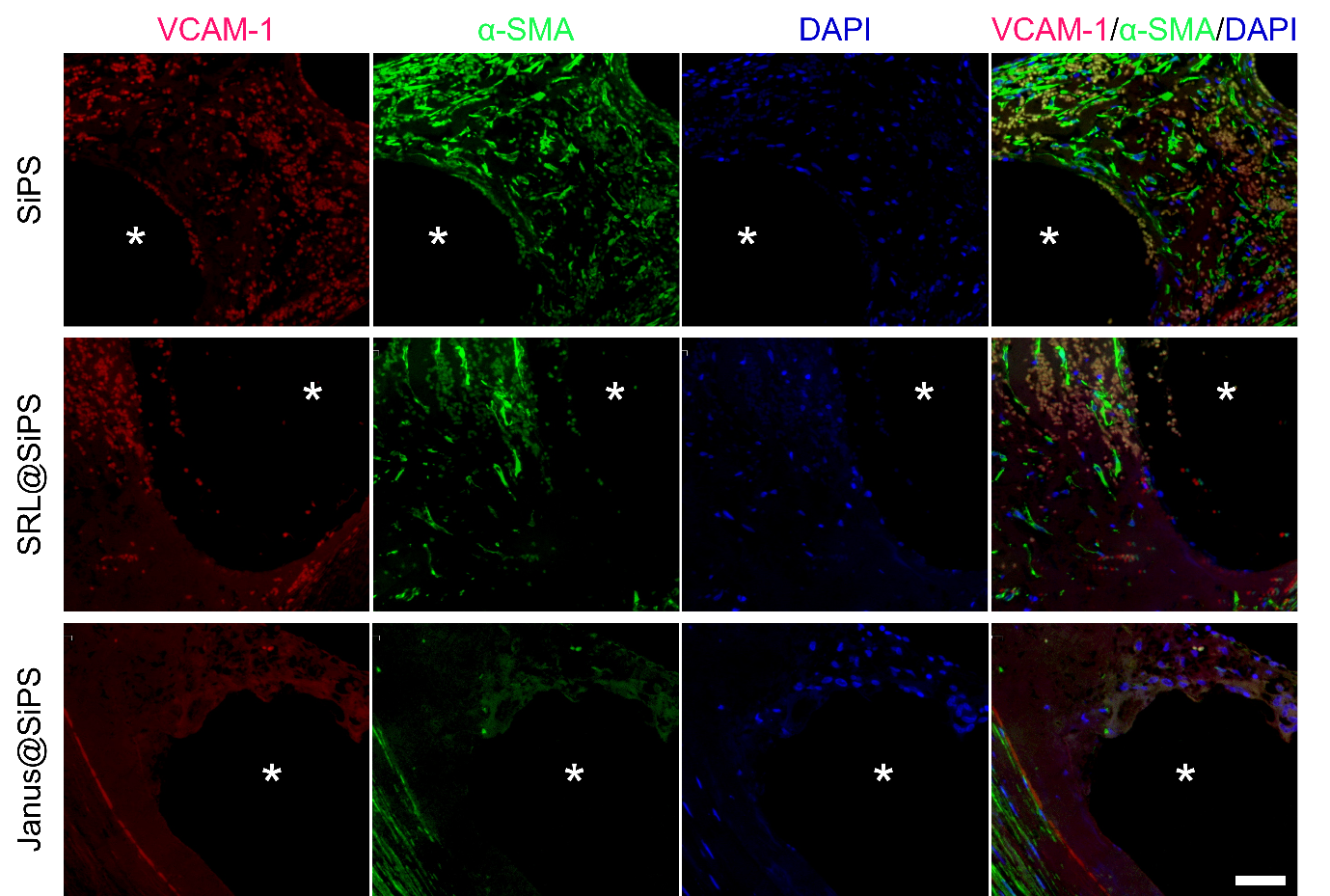


**Figure S28.** Representative VCAM-1 and α-SMA immunofluorescence images around the stent strut (white stars) captured at four weeks after stent placement in the porcine popliteal artery (scale bar: 50 μm).

**Figure S29.** Quantitative values corresponding to the heatmap depicting the degree of inflammatory cell infiltration in the study groups (n = 3). Data are shown as mean ± standard deviation (SD). Normality was tested using the Shapiro-Wilk method. For non-normally distributed data, statistical analysis was performed using the Kruskal-Wallis H test followed by pairwise comparisons with the Mann-Whitney U test, with significance at *p<0.05, **p<0.01, ***p<0.005, and ****p<0.001.

**Figure S30.** Quantitative values corresponding to the heatmap depicting the degree of elastic lamina changes in the study groups (n = 3). Data are shown as mean ± standard deviation (SD). Normality was tested using the Shapiro-Wilk method. For non-normally distributed data, statistical analysis was performed using the Kruskal-Wallis H test followed by pairwise comparisons with the Mann-Whitney U test, with significance at *p<0.05, **p<0.01, ***p<0.005, and ****p<0.001.

**Figure S31.** Quantitative values corresponding to the heatmap depicting the degree of TUNEL-positive cell coverage in the study groups (n = 3). Data are shown as mean ± standard deviation (SD). Normality was tested using the Shapiro-Wilk method. For non-normally distributed data, statistical analysis was performed using the Kruskal-Wallis H test followed by pairwise comparisons with the Mann-Whitney U test, with significance at *p<0.05, **p<0.01, ***p<0.005, and ****p<0.001.

**Figure S32.** Quantitative values corresponding to the heatmap depicting the degree of CD31-positive cell coverage in the study groups (n = 3). Data are shown as mean ± standard deviation (SD). Normality was tested using the Shapiro-Wilk method. For non-normally distributed data, statistical analysis was performed using the Kruskal-Wallis H test followed by pairwise comparisons with the Mann-Whitney U test, with significance at *p<0.05, **p<0.01, ***p<0.005, and ****p<0.001.

**Figure S33.** Quantitative values corresponding to the heatmap depicting the degree of VCAM-1-positive cell coverage in the study groups (n = 3). Data are shown as mean ± standard deviation (SD). Normality was tested using the Shapiro-Wilk method. For non-normally distributed data, statistical analysis was performed using the Kruskal-Wallis H test followed by pairwise comparisons with the Mann-Whitney U test, with significance at *p<0.05, **p<0.01, ***p<0.005, and ****p<0.001.

**Figure S34.** Quantitative values corresponding to the heatmap depicting the degree of SMA-positive cell coverage in the study groups (n = 3). Data are shown as mean ± standard deviation (SD). Normality was tested using the Shapiro-Wilk method. For non-normally distributed data, statistical analysis was performed using the Kruskal-Wallis H test followed by pairwise comparisons with the Mann-Whitney U test, with significance at *p<0.05, **p<0.01, ***p<0.005, and ****p<0.001.

**Table S1.** 3D printing parameters of BVSs with varying silica contents.

| Silica  wt% | Nozzle Diameter  (μm) | Temperature (℃) | Speed (mm/min) | Pressure  (MPa) |
| --- | --- | --- | --- | --- |
| 0 | 200 | 130 | 100 | ≥ 0.25 |
|  | 300 |  |  | ≥ 0.18 |
| 10 | 200 | 110 |  | ≥ 0.3 |
|  | 300 |  |  | ≥ 0.2 |
| 20 | 200 |  |  | ≥ 0.4 |
|  | 300 |  |  | ≥ 0.3 |
| 30 | 200 |  |  | ≥ 0.5 |
|  | 300 |  |  | ≥ 0.4 |

**Table S2.** Statistical comparisons of mechanical properties from the compressive radial force and bending tests of different 3D-printed stents.

| Groups | Mean ± SD | *p* value | Comparison of two groups (*p* value) | | | | | | |
| --- | --- | --- | --- | --- | --- | --- | --- | --- | --- |
|  |  |  | a | b | | c | | | d |
| Radial force [N] | | | | | | | | | |
| PS (a) | 4.35 ± 0.22 | < 0.001^a)^ | - | | < 0.001 | | < 0.001 | < 0.001 | |
| SiPS (b) | 6.32 ± 0.35 |  | < 0.001 | | - | | 0.242 | 0.202 | |
| SRL@SiPS (c) | 6.78 ± 0.27 |  | < 0.001 | | 0.242 | | - | 0.999 | |
| Janus@SiPS (d) | 6.81 ± 0.23 |  | < 0.001 | | 0.202 | | 0.999 | - | |
| Recovery rate [%] | | | | | | | | | |
| PS (a) | 97.14 ± 0.28 | < 0.001^a)^ | - | | < 0.001 | | < 0.001 | < 0.001 | |
| SiPS (b) | 92.35 ± 0.66 |  | < 0.001 | | - | | 0.601 | 0.998 | |
| SRL@SiPS (c) | 91.64 ± 0.14 |  | < 0.001 | | 0.601 | | - | 0.503 | |
| Janus@SiPS (d) | 92.45 ± 1.15 |  | < 0.001 | | 0.998 | | 0.503 | - | |
| Load of bending test [N] | | | | | | | | | |
| PS (a) | 0.44 ± 0.05 | < 0.005^a)^ | - | | 0.055 | | < 0.005 | < 0.005 | |
| SiPS (b) | 0.74 ± 0.08 |  | 0.055 | | - | | 0.154 | 0.055 | |
| SRL@SiPS (c) | 0.97 ± 0.10 |  | < 0.005 | | 0.154 | | - | 0.882 | |
| Janus@SiPS (d) | 1.04 ± 0.19 |  | < 0.005 | | 0.055 | | 0.882 | - | |

^a)^ One-way ANOVA test

**Table S3.** Statistical comparisons of wettability based on water contact angle measurements for different surfaces of 3D-printed specimens.

| Groups | Mean ± SD | *p* value | Comparison of two groups (*p* value) | | | | |
| --- | --- | --- | --- | --- | --- | --- | --- |
|  |  |  | a | b | c | d | e |
| Radial force [N] | | | | | | | |
| PS (a) | 75.33 ± 3.23 | < 0.001^a)^ | - | < 0.001 | < 0.001 | < 0.001 | < 0.001 |
| SiPS (b) | 43.01 ± 2.45 |  | < 0.001 | - | < 0.01 | < 0.001 | < 0.005 |
| SRL@SiPS (c) | 51.67 ± 1.70 |  | < 0.001 | < 0.01 | - | < 0.001 | < 0.001 |
| Ta@SiPS (d) | 31.02 ± 1.41 |  | < 0.001 | < 0.001 | < 0.001 | - | 0.832 |
| SRL-Ta@SiPS (e) | 33.01 ± 2.45 |  | < 0.001 | < 0.005 | < 0.001 | 0.832 | - |

^a)^ One-way ANOVA test

**Table S4.** Statistical comparisons of radial force from the compressive radial force test of different 3D-printed stents over a degradation period of 12 weeks.

^a)^ One-way ANOVA test

| Groups | Mean ± SD | *p* value | Comparison of two groups (*p* value) | | | | | | |
| --- | --- | --- | --- | --- | --- | --- | --- | --- | --- |
|  |  |  | a | b | | c | | | d |
| Week 0 [N] | | | | | | | | | |
| PS (a) | 4.36 ± 0.23 | < 0.001^a)^ | - | | < 0.001 | | < 0.001 | < 0.001 | |
| SiPS (b) | 6.32 ± 0.35 |  | < 0.001 | | - | | 0.718 | 0.682 | |
| SRL@SiPS (c) | 6.79 ± 0.28 |  | < 0.001 | | 0.718 | | - | 1.000 | |
| Janus@SiPS (d) | 6.81 ± 0.23 |  | < 0.001 | | 0.682 | | 1.000 | - | |
| Week 1 [N] | | | | | | | | | |
| PS (a) | 4.33 ± 0.33 | < 0.001^a)^ | - | | < 0.001 | | < 0.001 | < 0.001 | |
| SiPS (b) | 6.33 ± 0.37 |  | < 0.001 | | - | | 0.705 | 0.385 | |
| SRL@SiPS (c) | 6.81 ± 0.48 |  | < 0.001 | | 0.705 | | - | 0.952 | |
| Janus@SiPS (d) | 7.04 ± 0.45 |  | < 0.001 | | 0.385 | | 0.952 | - | |
| Week 2 [N] | | | | | | | | | |
| PS (a) | 4.46 ± 0.27 | < 0.001^a)^ | - | | < 0.001 | | < 0.001 | < 0.001 | |
| SiPS (b) | 6.40 ± 0.70 |  | < 0.001 | | - | | 0.414 | 0.535 | |
| SRL@SiPS (c) | 7.08 ± 1.05 |  | < 0.001 | | 0.414 | | - | 0.997 | |
| Janus@SiPS (d) | 6.99 ± 0.40 |  | < 0.001 | | 0.535 | | 0.997 | - | |
| Week 4 [N] | | | | | | | | | |
| PS (a) | 4.35 ± 0.37 | < 0.001^a)^ | - | | < 0.001 | | < 0.001 | < 0.001 | |
| SiPS (b) | 6.54 ± 0.49 |  | < 0.001 | | - | | 0.606 | 0.990 | |
| SRL@SiPS (c) | 7.09 ± 0.44 |  | < 0.001 | | 0.606 | | - | 0.786 | |
| Janus@SiPS (d) | 6.68 ± 0.29 |  | < 0.001 | | 0.990 | | 0.786 | - | |
| Week 8 [N] | | | | | | | | | |
| PS (a) | 4.53 ± 0.59 | < 0.001^a^ | - | | < 0.001 | | < 0.001 | < 0.001 | |
| SiPS (b) | 6.68 ± 0.89 |  | < 0.001 | | - | | 0.700 | 0.991 | |
| SRL@SiPS (c) | 7.16 ± 0.52 |  | < 0.001 | | 0.700 | | - | 0.518 | |
| Janus@SiPS (d) | 6.55 ± 0.48 |  | < 0.001 | | 0.991 | | 0.518 | - | |
| Week 12 [N] | | | | | | | | | |
| PS (a) | 4.41 ± 0.12 | < 0.001^a^ | - | | < 0.001 | | < 0.001 | < 0.001 | |
| SiPS (b) | 6.57 ± 0.57 |  | < 0.001 | | - | | 0.978 | 0.978 | |
| SRL@SiPS (c) | 6.74 ± 0.90 |  | < 0.001 | | 0.978 | | - | 1.000 | |
| Janus@SiPS (d) | 6.75 ± 0.89 |  | < 0.001 | | 0.978 | | 1.000 | - | |

**Table S5.** Statistical comparison of PCL crystallinity of different 3D-printed stents determined by XRD during accelerated degradation at 0, 1, 2, and 3 weeks.

^a)^ One-way ANOVA test

| Groups | Mean ± SD | *p* value | Comparison of two groups (*p* value) | | | | | | |
| --- | --- | --- | --- | --- | --- | --- | --- | --- | --- |
|  |  |  | a | b | | c | | | d |
| Week 0 [%] | | | | | | | | | |
| PS (a) | 51.97 ± 1.24 | > 0.05^a)^ | - | | 0.707 | | 0.850 | 0.926 | |
| SiPS (b) | 53.81 ± 2.15 |  | 0.707 | | - | | 0.993 | 0.967 | |
| SRL@SiPS (c) | 53.35 ± 2.15 |  | 0.850 | | 0.993 | | - | 0.997 | |
| Janus@SiPS (d) | 53.03 ± 1.84 |  | 0.926 | | 0.967 | | 0.997 | - | |
| Week 1 [%] | | | | | | | | | |
| PS (a) | 53.68 ± 2.34 | > 0.05^a)^ | - | | 0.998 | | 1.000 | 0.999 | |
| SiPS (b) | 53.99 ± 2.28 |  | 0.998 | | - | | 1.000 | 0.990 | |
| SRL@SiPS (c) | 53.86 ± 2.54 |  | 1.000 | | 1.000 | | - | 0.995 | |
| Janus@SiPS (d) | 53.46 ± 2.14 |  | 0.999 | | 0.990 | | 0.995 | - | |
| Week 2 [%] | | | | | | | | | |
| PS (a) | 54.55 ± 2.54 | > 0.05^a)^ | - | | 0.979 | | 0.928 | 0.946 | |
| SiPS (b) | 53.88 ± 2.34 |  | 0.979 | | - | | 0.996 | 0.999 | |
| SRL@SiPS (c) | 53.51 ± 1.94 |  | 0.928 | | 0.996 | | - | 1.000 | |
| Janus@SiPS (d) | 53.61 ± 1.79 |  | 0.946 | | 0.999 | | 1.000 | - | |
| Week 3 [%] | | | | | | | | | |
| PS (a) | 54.17 ± 2.38 | > 0.05^a)^ | - | | 0.994 | | 0.678 | 0.937 | |
| SiPS (b) | 53.74 ± 2.08 |  | 0.994 | | - | | 0.819 | 0.987 | |
| SRL@SiPS (c) | 52.25 ± 1.74 |  | 0.678 | | 0.819 | | - | 0.948 | |
| Janus@SiPS (d) | 53.18 ± 1.57 |  | 0.937 | | 0.987 | | 0.948 | - | |

**Table S6.** Statistical comparisons of hemolysis ratios between different 3D-printed stents.

| Groups | Mean ± SD | *p* value | Comparison of two groups (*p* value) | | | | | |
| --- | --- | --- | --- | --- | --- | --- | --- | --- |
|  |  |  | a | b | c | d | e | f |
| Hemolysis [%] | | | | | | | | |
| Triton X-100 (a) | 100.00 ± 0.01 | < 0.001^a)^ | - | < 0.01 | 0.677 | < 0.005 | < 0.005 | < 0.001 |
| PS (b) | 0.49 ± 0.14 |  | < 0.01 | - | 0.094 | 0.995 | 0.972 | < 0.001 |
| SiPS (c) | 0.22 ± 0.06 |  | 0.677 | 0.094 | - | < 0.05 | < 0.05 | < 0.001 |
| SRL@SiPS (d) | 0.40 ± 0.07 |  | < 0.005 | 0.995 | < 0.05 | - | 0.999 | < 0.001 |
| Ta@SiPS (e) | 0.19 ± 0.02 |  | < 0.005 | 0.972 | < 0.05 | 0.999 | - | < 0.001 |
| SRL-Ta@SiPS (f) | 0.18 ± 0.04 |  | < 0.001 | < 0.001 | < 0.001 | < 0.001 | < 0.001 | - |

^a)^ One-way ANOVA test

**Table S7.** Statistical comparisons of blood coagulation indices from the hemocompatibility assay of different surfaces of 3D-printed specimens.

| Groups | Mean ± SD | *p* value | Comparison of two groups (*p* value) | | | | |
| --- | --- | --- | --- | --- | --- | --- | --- |
|  |  |  | a | b | c | d | e |
| 10 min [%] | | | | | | | |
| PS (a) | 9.79 ± 1.07 | < 0.001^a)^ | - | 0.900 | 0.984 | < 0.05 | < 0.05 |
| SiPS (b) | 8.79 ± 0.25 |  | 0.900 | - | 0.996 | 0.218 | 0.180 |
| SRL@SiPS (c) | 9.20 ± 1.62 |  | 0.984 | 0.996 | - | 0.115 | 0.093 |
| Ta@SiPS (d) | 6.29 ± 0.41 |  | < 0.05 | 0.218 | 0.115 | - | 1.000 |
| SRL-Ta@SiPS (e) | 6.16 ± 0.31 |  | < 0.05 | 0.180 | 0.093 | 1.000 | - |
| 30 min [%] | | | | | | | |
| PS (a) | 14.54 ± 1.56 | < 0.001^a)^ | - | < 0.005 | 0.940 | < 0.001 | < 0.001 |
| SiPS (b) | 9.62 ± 0.87 |  | < 0.005 | - | < 0.001 | 0.263 | 0.766 |
| SRL@SiPS (c) | 15.40 ± 2.06 |  | 0.940 | < 0.001 | - | < 0.001 | < 0.001 |
| Ta@SiPS (d) | 7.25 ± 1.60 |  | < 0.001 | 0.263 | < 0.001 | - | 0.887 |
| SRL-Ta@SiPS (e) | 8.29 ± 2.28 |  | < 0.001 | 0.766 | < 0.001 | 0.887 | - |

^a)^ One-way ANOVA test

**Table S8.** Statistical comparisons of platelet activation ratios from platelet observation of different surfaces of 3D-printed specimens.

| Groups | Mean ± SD | *p* value | Comparison of two groups (*p* value) | | | | |
| --- | --- | --- | --- | --- | --- | --- | --- |
|  |  |  | a | b | c | d | e |
| Resting [%] | | | | | | | |
| PS (a) | 6.07 ± 2.44 | < 0.005^a)^ | - | 0.201 | 0.908 | < 0.005 | < 0.05 |
| SiPS (b) | 15.22 ± 3.14 |  | 0.201 | - | 0.6614 | 0.228 | 0.893 |
| SRL@SiPS (c) | 9.64 ± 2.77 |  | 0.908 | 0.6614 | - | < 0.05 | 0.187 |
| Ta@SiPS (d) | 24.07 ± 4.78 |  | < 0.005 | 0.228 | < 0.05 | - | 0.728 |
| SRL-Ta@SiPS (e) | 18.95 ± 1.95 |  | < 0.05 | 0.893 | 0.187 | 0.728 | - |
| Dendrite [%] | | | | | | | |
| PS (a) | 29.95 ± 5.84 | < 0.001^a)^ | - | < 0.001 | 0.113 | < 0.001 | < 0.001 |
| SiPS (b) | 65.86 ± 2.48 |  | < 0.001 | - | < 0.001 | 0.975 | 0.916 |
| SRL@SiPS (c) | 40.34 ± 9.20 |  | 0.113 | < 0.001 | - | < 0.001 | < 0.001 |
| Ta@SiPS (d) | 63.42 ± 7.26 |  | < 0.001 | 0.975 | < 0.001 | - | 0.999 |
| SRL-Ta@SiPS (e) | 62.40 ± 4.46 |  | < 0.001 | 0.916 | < 0.001 | 0.999 | - |
| Spreading [%] | | | | | | | |
| PS (a) | 63.98 ± 7.19 | < 0.001^a)^ | - | < 0.001 | < 0.05 | < 0.001 | < 0.001 |
| SiPS (b) | 18.92 ± 2.63 |  | < 0.001 | - | < 0.001 | 0.537 | 1.000 |
| SRL@SiPS (c) | 50.02 ± 6.42 |  | < 0.05 | < 0.001 | - | < 0.001 | < 0.001 |
| Ta@SiPS (d) | 12.51 ± 3.80 |  | < 0.001 | 0.537 | < 0.001 | - | 0.578 |
| SRL-Ta@SiPS (e) | 18.65 ± 4.59 |  | < 0.001 | 1.000 | < 0.001 | 0.578 | - |

^a)^ One-way ANOVA test

**Table S9.** Statistical comparisons of platelet density from platelet activation analysis of different surfaces of 3D-printed specimens.

| Groups | Mean ± SD | *p* value | Comparison of two groups (*p* value) | | | | |
| --- | --- | --- | --- | --- | --- | --- | --- |
|  |  |  | a | b | c | d | e |
| Platelet density [$\times$10^4^ mm^-2^] | | | | | | | |
| PS (a) | 3.50 ± 0.38 | < 0.001^a)^ | - | < 0.05 | 0.371 | < 0.001 | < 0.001 |
| SiPS (b) | 2.36 ± 0.38 |  | < 0.05 | - | 0.490 | 0.165 | 0.056 |
| SRL@SiPS (c) | 2.89 ± 0.27 |  | 0.371 | 0.490 | - | < 0.05 | < 0.005 |
| Ta@SiPS (d) | 1.56 ± 0.14 |  | < 0.001 | 0.165 | < 0.05 | - | 0.948 |
| SRL-Ta@SiPS (e) | 1.33 ± 0.62 |  | < 0.001 | 0.056 | < 0.005 | 0.948 | - |

^a)^ One-way ANOVA test

**Table S10.** Statistical comparisons of HUVEC coverage ratios from DAPI/phalloidin fluorescence staining of different surfaces of 3D-printed specimens.

| Groups | Mean ± SD | *p* value | Comparison of two groups (*p* value) | | | | |
| --- | --- | --- | --- | --- | --- | --- | --- |
|  |  |  | a | b | c | d | e |
| HUVEC coverage ratio [%] | | | | | | | |
| PS (a) | 4.30 ± 3.21 | < 0.05^a)^ | - | < 0.001 | < 0.05 | < 0.001 | < 0.001 |
| SiPS (b) | 17.65 ± 2.27 |  | < 0.001 | - | 0.278 | 0.053 | 0.343 |
| SRL@SiPS (c) | 13.00 ± 3.16 |  | < 0.05 | 0.278 | - | < 0.005 | < 0.05 |
| Ta@SiPS (d) | 24.74 ± 1.86 |  | < 0.001 | 0.053 | < 0.005 | - | 0.708 |
| Ta-SRL@SiPS (e) | 21.95 ± 2.60 |  | < 0.001 | 0.343 | < 0.05 | 0.708 | - |

^a)^ One-way ANOVA test

**Table S11.** Statistical comparisons of CCK assay results evaluating the cell viability of HUVECs cultured on different 3D-printed specimens for three and five days.

| Groups | Mean ± SD | *p* value | Comparison of two groups (*p* value) | | | | |
| --- | --- | --- | --- | --- | --- | --- | --- |
|  |  |  | a | b | c | d | e |
| Day 3 [%] | | | | | | | |
| PS (a) | 100.00 ± 3.04 | < 0.001^a)^ | -  < 0.005 | < 0.05  < 0.005 | 0.1266 | < 0.001 | < 0.001 |
| SiPS (b) | 183.00 ± 11.72 |  | < 0.05 | - | 0.779 | < 0.01 | 0.054 |
| SRL@SiPS (c) | 157.00 ± 14.84 |  | 0.1266 | 0.7797 | - | < 0.001 | < 0.005 |
| Ta@SiPS (d) | 273.00 ± 27.77 |  | < 0.001 | < 0.01 | < 0.001 | - | 0.845 |
| Ta-SRL@SiPS (e) | 250.00 ± 31.67 |  | < 0.001 | 0.0537 | < 0.005 | 0.845 | - |
| Day 5 [%] | | | | | | | |
| PS (a) | 144.45 ± 21.77 | < 0.001^a)^ | - | < 0.001 | < 0.05 | < 0.001 | < 0.001 |
| SiPS (b) | 260.00 ± 33.26 |  | < 0.001 | - | 0.546 | < 0.001 | < 0.005 |
| SRL@SiPS (c) | 224.98 ± 37.91 |  | < 0.05 | 0.546 | - | < 0.001 | < 0.001 |
| Ta@SiPS (d) | 388.00 ± 35.00 |  | < 0.001 | < 0.001 | < 0.001 | - | 0.712 |
| Ta-SRL@SiPS (e) | 359.25 ± 36.25 |  | < 0.001 | < 0.005 | < 0.001 | 0.712 | - |

^a)^ One-way ANOVA test

**Table S12.** Statistical comparisons of SMC coverage ratios from DAPI/phalloidin fluorescence staining of different surfaces of 3D-printed specimens.

| Groups | Mean ± SD | *p* value | Comparison of two groups (*p* value) | | | | |
| --- | --- | --- | --- | --- | --- | --- | --- |
|  |  |  | a | b | c | d | e |
| SMC coverage ratio [%] | | | | | | | |
| PS (a) | 15.65 ± 3.21 | < 0.001^a)^ | - | 0.238 | < 0.005 | 0.080 | < 0.005 |
| SiPS (b) | 21.38 ± 3.6 |  | 0.238 | - | < 0.001 | 0.942 | < 0.001 |
| SRL@SiPS (c) | 2.29 ± 1.15 |  | < 0.005 | < 0.001 | - | < 0.001 | 0.999 |
| Ta@SiPS (d) | 23.26 ± 4.70 |  | 0.080 | 0.942 | < 0.001 | - | < 0.001 |
| Ta-SRL@SiPS (e) | 2.97 ± 1.40 |  | < 0.005 | < 0.001 | 0.999 | < 0.001 | - |

^a)^ One-way ANOVA test

**Table S13.** Statistical comparisons of CCK assay results evaluating the cell viability of VSMCs cultured on different 3D-printed specimens for three and five days.

| Groups | Mean ± SD | *p* value | Comparison of two groups (*p* value) | | | | |
| --- | --- | --- | --- | --- | --- | --- | --- |
|  |  |  | a | b | c | d | e |
| Day 3 [%] | | | | | | | |
| PS (a) | 100.00 ± 9.61 | < 0.001^a)^ | -  < 0.005 | < 0.005  < 0.005 | < 0.05 | < 0.001 | 0.1015 |
| SiPS (b) | 176.00 ± 24.00 |  | < 0.005 | - | < 0.001 | 0.979 | < 0.001 |
| SRL@SiPS (c) | 37.25 ± 9.98 |  | < 0.05 | < 0.001 | - | < 0.001 | 0.902 |
| Ta@SiPS (d) | 186.20 ± 21.00 |  | < 0.001 | 0.979 | < 0.001 | - | < 0.001 |
| Ta-SRL@SiPS (e) | 52.86 ± 12.09 |  | 0.1015 | < 0.001 | 0.902 | < 0.001 | - |
| Day 5 [%] | | | | | | | |
| PS (a) | 181.18 ± 4.81 | < 0.001^a)^ | - | 0.143 | < 0.001 | < 0.005 | < 0.001 |
| SiPS (b) | 224.90 ± 33.31 |  | 0.143 | - | < 0.001 | 0.468 | < 0.001 |
| SRL@SiPS (c) | 49.02 ± 18.18 |  | < 0.001 | < 0.001 | - | < 0.001 | 0.992 |
| Ta@SiPS (d) | 254.90 ± 43.31 |  | < 0.005 | 0.468 | < 0.001 | - | < 0.001 |
| Ta-SRL@SiPS (e) | 56.86 ± 12.09 |  | < 0.001 | < 0.001 | 0.992 | < 0.001 | - |

^a)^ One-way ANOVA test

**Table S14.** Statistical comparisons of quantitative results from angiographic and optical coherence tomographic observation after implantation of different 3D-printed stents for four weeks.

| Groups | Mean ± SD | *p* value | Comparison of two groups (*p* value) | | |
| --- | --- | --- | --- | --- | --- |
|  |  |  | a | b | c |
| Luminal diameter [mm] | | | | | |
| SiPS (a) | 1.78 ± 0.28 | < 0.001^b)^ | - | < 0.001 | < 0.001 |
| SRL@SiPS (b) | 2.57 ± 0.18 |  | < 0.001 | - | < 0.005 |
| Janus@SiPS (c) | 2.85 ± 0.05 |  | < 0.001 | < 0.005 | - |
| Neointima thickness [mm] | | | | | |
| SiPS group (a) | 0.33 ± 0.03 | < 0.001^b)^ | - | < 0.001 | < 0.001 |
| SRL@SiPS (b) | 0.27 ± 0.01 |  | < 0.001 | - | < 0.005 |
| Janus@SiPS (c) | 0.23 ± 0.01 |  | < 0.001 | < 0.005 | - |
| Stenotic area [%] | | | | | |
| SiPS group (a) | 50.36 ± 3.62 | < 0.001^b)^ | - | < 0.001 | < 0.001 |
| SRL@SiPS (b) | 31.68 ± 2.69 |  | < 0.001 | - | < 0.001 |
| Janus@SiPS (c) | 18.28 ± 1.47 |  | < 0.001 | < 0.001 | - |

^b)^ Kruskal–Wallis test

**Table S15.** Statistical comparisons of quantitative results from immunofluorescence staining and histological analysis after implantation of different 3D-printed stents for four weeks.

| Groups | Mean ± SD | *p* value | Comparison of two groups (*p* value^C)^) | | |  |
| --- | --- | --- | --- | --- | --- | --- |
|  |  |  | a | b | c |  |
| Inflammatory cell filtration [degree] | | | | | |  |
| SiPS (a) | 4.31 ± 0.60 | < 0.001^b)^ | - | < 0.001 | < 0.001 |  |
| SRL@SiPS (b) | 2.25 ± 0.68 |  | < 0.001 | - | < 0.05 |  |
| Janus@SiPS (c) | 1.68 ± 0.47 |  | < 0.001 | < 0.05 | - |  |
| Elastic lamina change [degree] | | | | | |  |
| SiPS (a) | 2.87 ± 0.61 | < 0.001^b)^ | - | < 0.001 | < 0.001 |  |
| SRL@SiPS (b) | 1.56 ± 0.51 |  | < 0.001 | - | 0.309 |  |
| Janus@SiPS (c) | 1.25 ± 0.44 |  | < 0.001 | 0.309 | - |  |
| TUNEL-positive coverage [degree] | | | | | |  |
| SiPS (a) | 2.87 ± 0.50 | < 0.001^b)^ | - | < 0.05 | < 0.005 |  |
| SRL@SiPS (b) | 3.43 ± 0.62 |  | < 0.05 | - | < 0.001 |  |
| Janus@SiPS (c) | 2.18 ± 0.54 |  | < 0.005 | < 0.001 | - |  |
| CD31-positive coverage [degree] | | | | | |  |
| SiPS (a) | 4.31 ± 0.60 | < 0.001^b)^ | - | < 0.001 | < 0.001 |  |
| SRL@SiPS (b) | 2.93 ± 0.44 |  | < 0.001 | - | < 0.001 |  |
| Janus@SiPS (c) | 1.62 ± 0.50 |  | < 0.001 | < 0.001 | - |  |
| VCAM-1-positive coverage [degree] | | | | | | |
| SiPS (a) | 4.06 ± 0.57 | < 0.001^b)^ | - | < 0.001 | < 0.001 |  |
| SRL@SiPS (b) | 2.43 ± 0.72 |  | < 0.001 | - | < 0.001 |  |
| Janus@SiPS (c) | 1.56 ± 0.51 |  | < 0.001 | < 0.001 | - |  |
| α-SMA-positive coverage [degree] | | | | | |  |
| SiPS (a) | 3.68 ± 0.60 | < 0.001^b)^ | - | < 0.001 | < 0.001 |  |
| SRL@SiPS (b) | 1.87 ± 0.50 |  | < 0.001 | - | < 0.05 |  |
| Janus@SiPS (c) | 1.31 ± 0.47 |  | < 0.001 | < 0.05 | - |  |

^b)^ Kruskal–Wallis test
